# Bokeelamides: Lipopeptides from Bacteria Associated with Marine Egg Masses

**DOI:** 10.1021/acs.orglett.4c03470

**Published:** 2024-11-01

**Authors:** Rose Campbell, Lois Kyei, Karla Piedl, Zheye Zhang, Ming Chen, Emily Mevers

**Affiliations:** Department of Chemistry, Virginia Tech, Blacksburg, Virginia 24061, United States

## Abstract

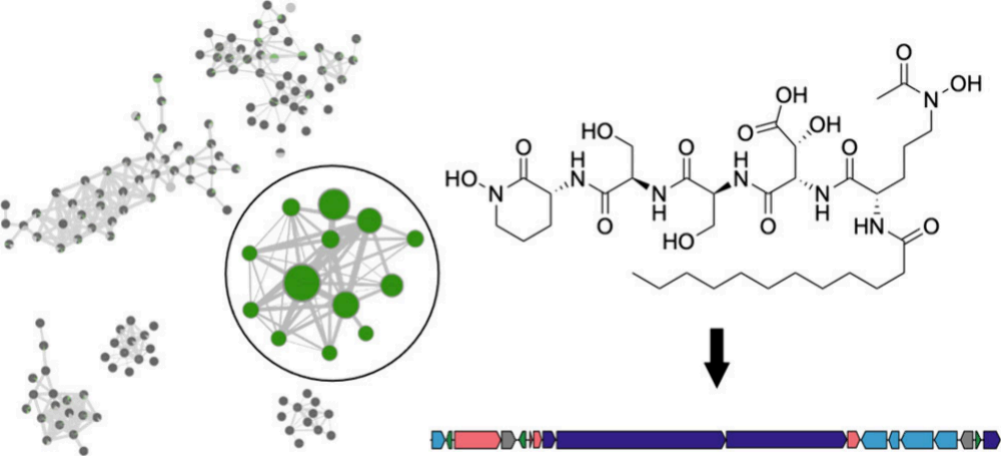

Moon snails (family: Naticidae) lay egg masses that are rich in bacterial species distinct from the surrounding environment. We hypothesized that this microbiome chemically defends the moon snail eggs from predation and pathogens. Herein, we report the discovery of bokeelamides, new lipopeptides from the egg mass-associated bacterium, *Ectopseudomonas khazarica*, which were discovered using mass spectrometry (MS)-based metabolomics. The structures of the bokeelamides were elucidated using two-dimensional (2D) nuclear magnetic resonance (NMR), tandem MS, Marfey’s, and genomic analyses.

Natural products are commonly deployed as defensive agents and have fascinated chemists for generations due to their structural complexity and pharmaceutical potential.^1−5^ Many animals are known to form symbiotic relationships with chemically rich microorganisms, where the host provides essential nutrients in exchange for defensive agents.^4,6−8^ Recently, our group showed that egg masses laid by *Neverita delessertiana*, a carnivorous moon snail, contain a chemically rich core bacterial microbiome that is distinct from the surrounding environment.^9^ We hypothesize that this microbiome produces natural products to protect the egg masses from biofouling and predation.^10^ Herein, we used a metabolomic approach to identify a new family of lipopeptides, bokeelamides.

As part of our continued effort to probe the natural product potential of moon snail egg mass-associated bacteria, we reanalyzed high-resolution tandem mass spectrometry (HR-MSMS) data acquired on semicrude fractions representing 66 distinct bacterial strains using Global Natural Product Social (GNPS) molecular networking (Figure S1 of the Supporting Information). One subnetwork contained 13 nodes with precursor masses ranging from 762 to 846 Da (Figure 1) with no known library matches within the subnetwork. Traditional dereplication efforts, searching the molecular formula and/or the parent mass in AntiBase,^11^ NPAtlas,^12^ SIRIUS,^13^ and SciFinder, failed to yield any hits to known chemistry. Although dereplication by SIRIUS yielded no exact matches, SIRIUS uses patterns in the tandem mass spectral data to derive structural information, and this analysis did suggest that these metabolites are peptidic. The SIRIUS analysis revealed the presence of distinguishable neutral losses corresponding to two serine residues (Figures S2–S4 of the Supporting Information).^13^ Many of the nodes within the subnetwork differ from one another by 2 or 14 Da, suggesting that the structural modification involved a change in either the degree of unsaturation (DoU) or methylene incorporation, respectively. The nodes are produced by five bacterial strains in our library, including two *Ectopseudomonas* strains (EM133 and EM143), a *Bacillus* sp. (EM91), a *Pseudoalteromonas* sp. (EM135), and a *Vibrio* sp. (EM103). Only EM133, *Ectopseudomonas khazarica*, produces all 13 masses (Table S1 of the Supporting Information). Reverse-phase high-performance liquid chromatography led to the purification of the four major compounds (Figure S5 of the Supporting Information), named bokeelamides A–D (**1**–**4**).

**Figure 1:**
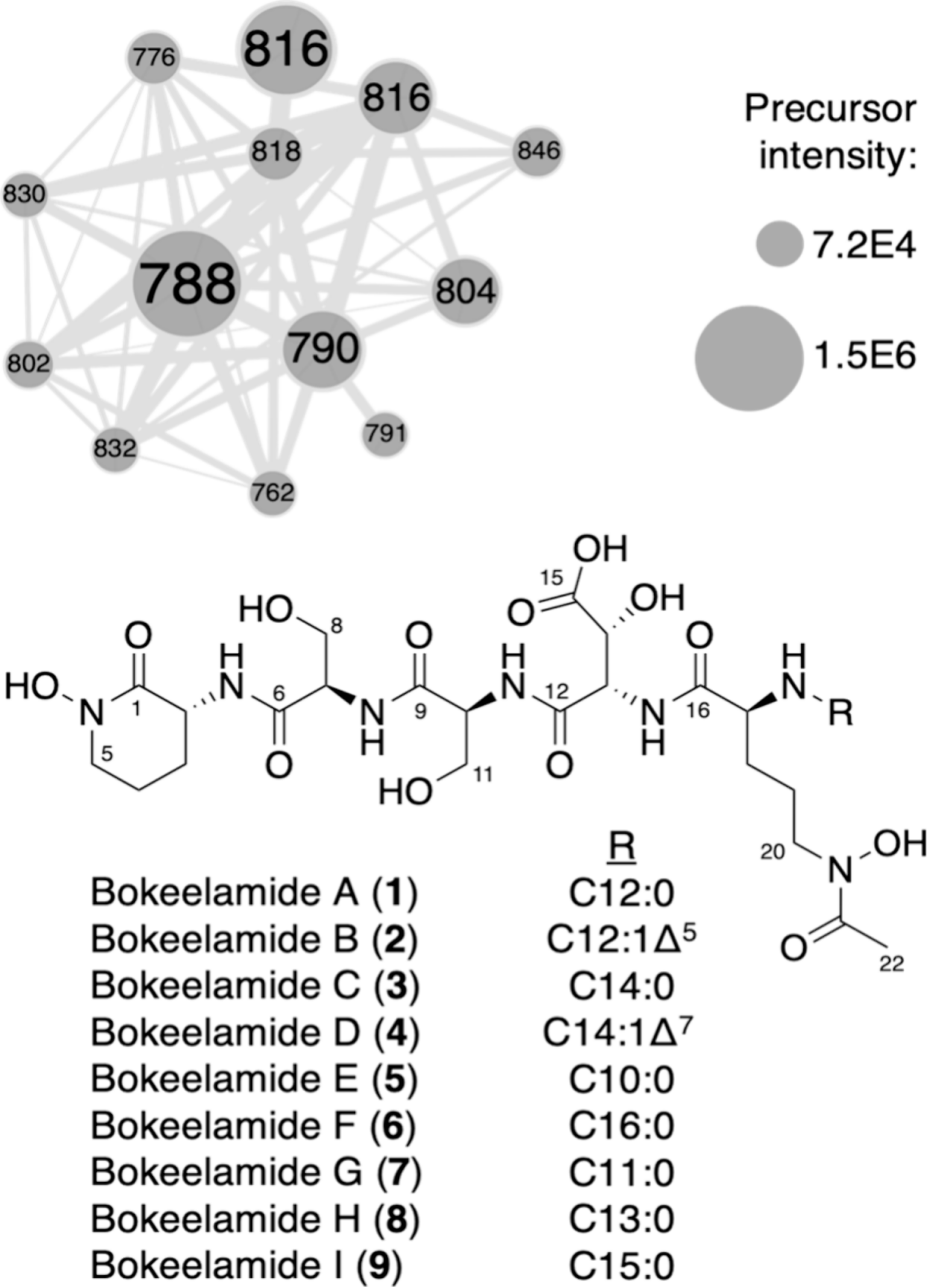
Subnetwork containing precursor masses (node labels) for bokeelamides.

We began our structure elucidation efforts by analyzing two-dimensional (2D) nuclear magnetic resonance (NMR) [^1^H, ^13^C, gradient-selected heteronuclear single quantum coherence (gHSQC),double-quantum-filtered correlation spectroscopy (dqfCOSY), total correlation spectroscopy (TOCSY), heteronuclear two-bond correlation (H2BC), and heteronuclear multiple-bond correlation (HMBC)] spectra of compound **1** as it was the most abundant and appeared to be the least complicated. Analysis of the high-resolution mass spectrometry (HRMS) data indicated that compound **1** had a molecular formula (MF) of C_34_H_59_N_7_O_14_. Partial structures were assembled using key 2D NMR correlations, revealing the presence of two modified ornithines (Orn), two serines (Ser), a β-hydroxy aspartic acid (βOH-Asp), and a saturated lipid (C12:0) (Figures S6–S12 and Table S2 of the Supporting Information). A key HMBC between H-5 (δ_H_ 3.46) and C-1 (δ_C_ 165.0) indicated that the terminal Orn_1_ residue has been cyclized. Reanalysis of the heteronuclear single-quantum correlation (HSQC) spectrum confirmed the presence of eight exchangeable protons, five of which (δ_H_ 7.76, 7.80, 8.07, 8.26, and 8.27) are part of the peptide backbone. HMBCs between these exchangeable protons and the neighboring amino acids established connectivity between the individual residues (Figure 2A). However, C_2_H_5_O_3_ was still left unassigned, and this included an obvious singlet methyl, C-22 (δ_H_ 1.99; δ_C_ 20.4). Analysis of the HMBCs from H_3_-22 showed a correlation to an unassigned carbonyl, C-21 (δ_C_ 170.4), matching the expected chemical shifts for an acetate moiety. In addition, an exchangeable proton at δ_H_ 5.31 had a TOCSY correlation to H_3_-22; however, there were no correlations between these protons and the established peptide core. Therefore, we used tandem mass spectrometry (MSMS) to locate the positions of the remaining unassigned acetate moiety and hydroxyl groups. A key b_1_ fragment at *m*/*z* 355.2584 represents Orn_2_-C12:0 but contains an additional C_2_H_4_O_2_, suggesting that Orn_2_ is both acetylated and hydroxylated (Figure 2B). Another key fragment, y_1_, was *m*/*z* 131.0813 indicating that cyclic Orn_1_ is also hydroxylated, forming N^5^OH-Orn_1_ and establishing the final planar structure of compound **1** as (cyclic-N^5^OH-Orn_1_)-Ser_1_-Ser_2_-(βOH-Asp)-(AcN^5^OH-Orn_2_)-C12:0.

**Figure 2:**
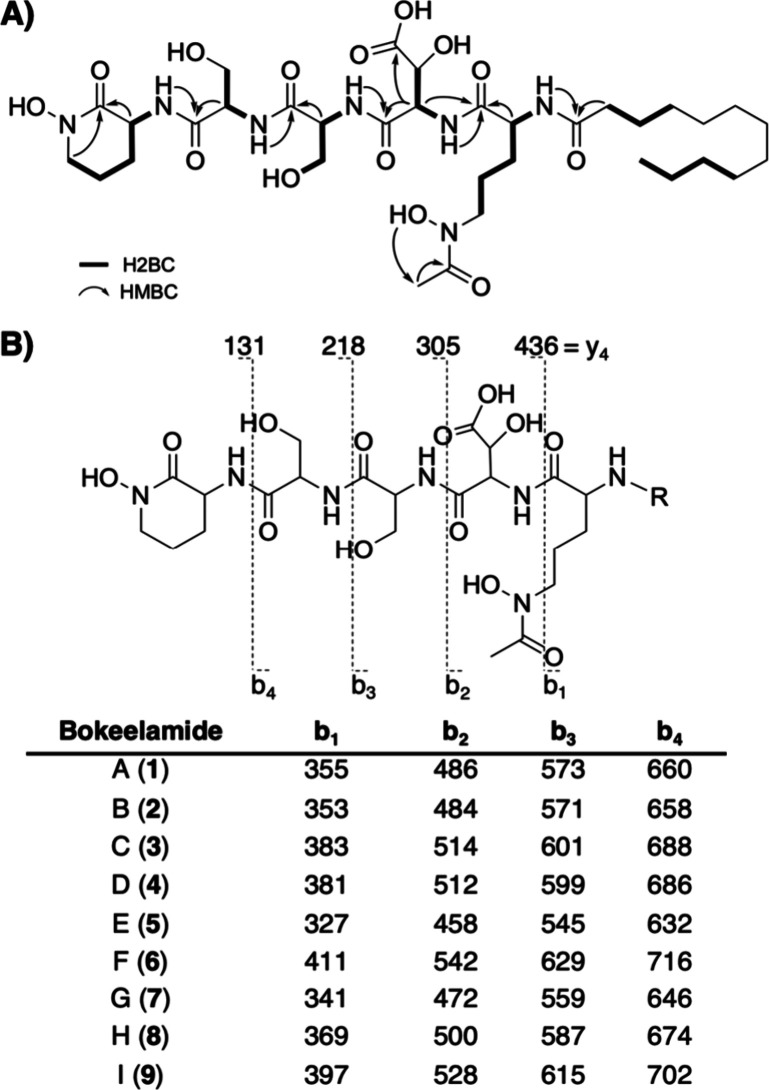
(A) Key 2D NMR correlations for bokeelamide A (**1**) and (B) observed fragmentation of bokeelamides, where all analogues had the same y_1–4_ and varying b_1–4_.

The MFs and fragmentation patterns of compounds **2**–**4** are highly similar to those of compound **1**. All y fragments were present in compounds **1**–**4**, but the b fragments, representing lipid-containing portions, were shifted by the observed difference in the precursor mass (Figure 2B). Thus, the modifications within these analogues are restricted to the b_1_ fragment (Orn_2_ and the lipid). As AcN^5^OH-Orn_2_-lipid did not fragment independently, we used NMR experiments to identify the structural modifications. Compound **2** is 2 Da less than compound **1**, which corresponds to an increase in DoU. The HSQC spectrum revealed the presence of two sp^2^-hybridized carbons that are part of an olefin (δ_H_ 5.30 and 5.33; δ_C_ 129.1 and 130.2). H2BCs confirmed that compound **2** incorporated a C12:1Δ^5^ lipid (Figures S13–S19 and Table S3 of the Supporting Information). By HRMS, compounds **3** and **4** both incorporate an additional C_2_H_4_ unit (28 Da) compared to compounds **1** and **2**, respectively, which was hypothesized to represent an extension of the lipid. The NMR data for compound **3** was similar to those for compound **1**, with the exception that the CH_2_ envelope integrated to 18 versus 16. Similarly, the NMR data for compound **4** was similar to those for compound **2**, but analysis of H2BC spectra confirmed that compound **4** incorporates a C14:1Δ^7^ lipid rather than a C12:1Δ^5^ lipid (Figures S20–S31 and Tables S4 and S5 of the Supporting Information). To determine the stereoconfiguration of the olefin in compounds **2** and **4**, we compared the experimental ^13^C shifts of the allylic methylenes to predicted chemical shifts of the *trans*/*cis* analogues using ChemDraw. All allylic methylenes were between 26.3 and 26.7 ppm, which matched closely to the *cis* configuration (26.7–27.7 ppm) over the *trans* configuration (32.7–33.7 ppm).

The GNPS cluster containing compounds **1**–**4** consisted of 13 nodes, with 7 nodes representing minor analogues with isolated yields too low for 2D NMR analysis. All nodes contained the same y_1–4_ ions, indicating that the modifications of the minor analogues were confined to either Orn_2_ or the lipid. The MFs of compounds **5** and **6** indicated a loss of two methylenes from compound **1** and a gain in two methylenes from compound **3**, suggesting incorporation of C10:0 and C16:0 lipids, respectively. The MFs for compounds **7**–**9** differ from compounds **5**, **1**, and **3**, respectively, by the addition of one methylene, which was located using pseudo-MS^3^ experiments, where the deacetylated b_1_ ion (OH-Orn_2_-lipid fragment) observed in the precursor spectrum was selected for further fragmentation. This generated fragments at *m*/*z* 131 (N^5^OH-Orn_2_), 114 (deaminated N^5^OH-Orn_2_), or 113 (Orn_2_) (Figures S32–S34 of the Supporting Information) indicating that all modifications were restricted to the lipids, which were now identified as C11:0 (**7**), C13:0 (**8**), and C15:0 (**9**). The fragmentation patterns for the final two nodes, 802 and 830, indicated incorporation of C13:1 and C15:1, respectively, but the position of the unsaturation could not be identified.

Absolute stereochemistry of compounds **1**–**4** was determined by Marfey’s analysis. An aliquot of each compound was hydrolyzed and derivatized with 1-fluoro-2,4-dinitrophenyl-5-l-alanine amide (l-FDAA). A liquid chromatography–mass spectrometry (LCMS) comparison of the derivatized hydrolysate products to authentic standards revealed the presence of l-*erythro*-βOH-Asp (51.8 min), both l- and d-Ser (51.5 and 52.5 min), and both l- and d-Orn (83.6 and 78.4 min) (Figures S35–S38 of the Supporting Information). To unequivocally assign the stereoconfiguration of the two Ser and Orn residues, we turned to genomic analysis. *E. khazarica* EM133 genomic DNA was sequenced using the Oxford Nanopore platform, assembled by SeqCenter, and identified by the Type Genome Server (Figure S39 and Table S6 of the Supporting Information). AntiSMASH 7.1.0^14^ analysis of the assembled genome identified eight biosynthetic gene clusters (BGCs), with one BGC annotated as a hybrid polyketide synthase (PKS)/nonribosomal peptide synthetase (NRPS) metallophore cluster (Figure 3A). This BGC, named *bka*, contains five NRPS modules with adenylation domains predicted to load the amino acids observed in bokeelamides (Figure 3B). Modules 3 and 4 are predicted to install the Ser residues, while modules 1 and 5 install the modified Orn residues. Excitedly, of these, only modules 4 (Ser_1_) and 5 (Orn_1_) contained epimerization domains, allowing us to assign the absolute stereoconfiguration of compounds **1**–**9** as d-(cyclic-N^5^OH-Orn_1_)-d-Ser_1_-l-Ser_2_-l-(*erythro*-βOH-Asp)-l-(AcN^5^OH-Orn_2_)-lipid.

**Figure 3:**
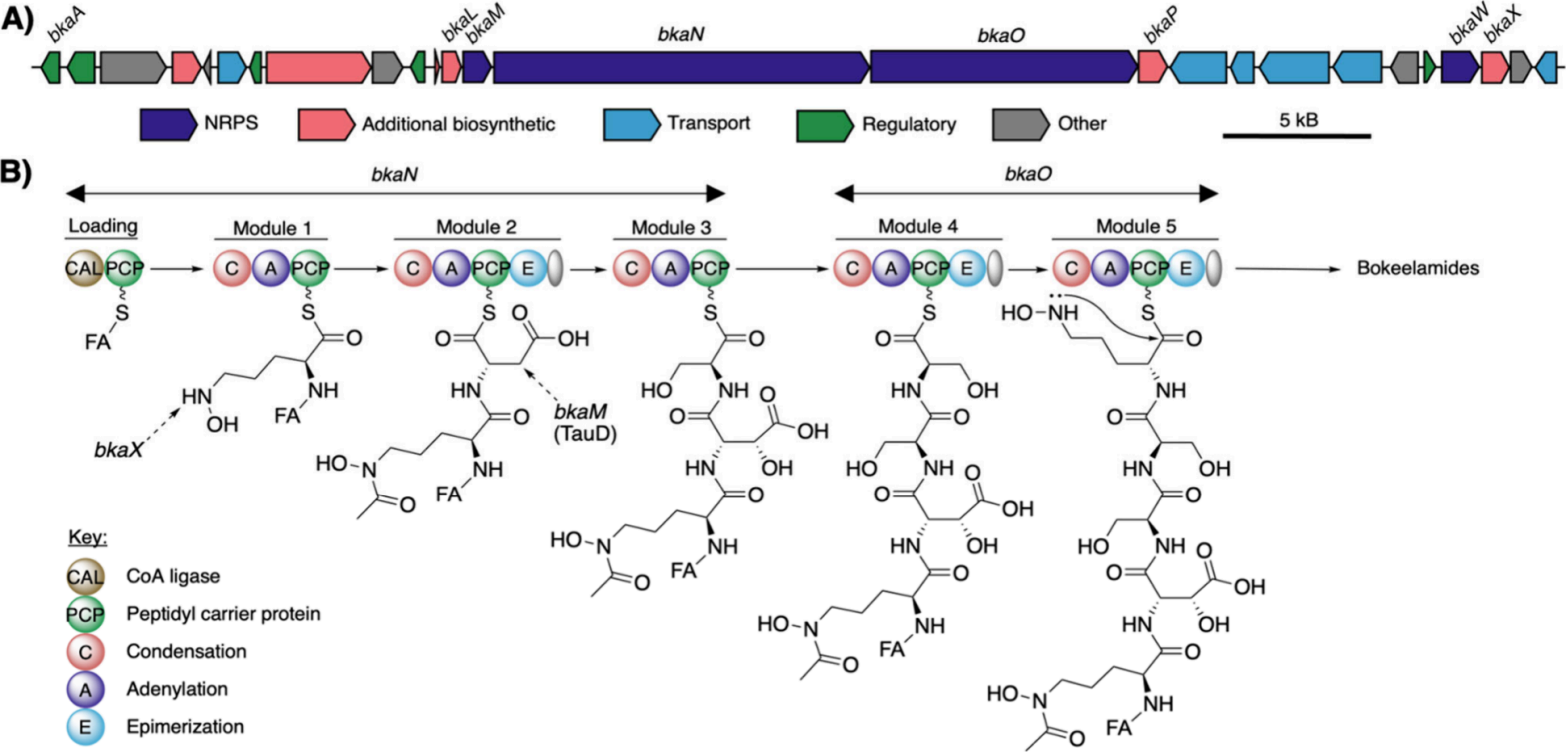
(A) Putative BGC responsible for producing bokeelamides and (B) putative enzymatic route to the production of bokeelamides.

Detailed analysis of the *bka* BGC provided key biochemical and structural information in support of the structure of bokeelamides. The lipid tail is loaded onto the peptidyl carrier protein (PCP) by a CoA ligase (CAL), which is then followed by five NRPS domains that load N^5^OH-Orn_2_, Asp, Ser_2_, Ser_1_, and N^5^OH-Orn_1_. Ornithine 5-monooxygenase, *bkaW*, is predicted to hydroxylate both Orn residues, producing N^5^OH-Orn prior to loading on the adenylation domains.^15^ The acylation of Orn_2_ is believed to be installed by *bkaX*, a *N*-acetyltransferase, after Orn_2_ has been incorporated into the linear peptide (Table S7 of the Supporting Information). Furthermore, TauD family TβH_Asp_ aspartyl β-hydroxylase (*bkaM*) has precedent to hydroxylate the β position of Asp, resulting in the observed *R* configuration.^16^ The role of the epimerization domain within module 2 is unknown as Marfey’s analysis unequivocally assigns βOH-Asp as 2*S*,3*R*-Asp (*erythro*). Previous literature precedence suggests that BGCs containing both TauD and an epimerization domain within the Asp module results in d-*threo*-βOH-Asp (2*R*,3*R*-Asp);^16^ however, there are exceptions to this finding.^17^ Finally, the biosynthesis of bokeelamides is hypothesized to be terminated through intramolecular cyclization of terminal N^5^OH-Orn_1_. However, it is unclear whether this is catalyzed by *bkaL* (NRPS thioesterase) or *bkaP* (an esterase).

Structurally, bokeelamides contain several distinct characteristics of siderophores, including incorporation of both hydroxamates and α-hydroxycarboxylates, thus indicating that compounds **1**–**9** are likely functioning as siderophores.^18^ The hydroxymate and βOH-Asp moieties are known to bind Fe^3+^ and are commonly found in peptidic siderophores, including amphibactins,^19^ loihichelins,^20^ amychelins,^18^ and potashchelins.^21^ Many of these siderophores also belong to the large class of amphiphilic lipopeptides, which attach a variety of lipids to a peptidic portion that contains multiple iron-binding ligands.^22,23^ The long lipid tail anchors the siderophore to the membrane, keeping it close to the cell membrane. The *bka* BGC further supports that bokeelamides are functioning as siderophores as several genes encode for ferric hydroxamate ABC transporters and TonB-dependent siderophore receptors (*bkaQ*-*T*,*Z*; Table S7 of the Supporting Information).^24^ Reevaluation of the LCMS chromatograms revealed the presence of both apo- and Fe^3+^-bokeelamides (Figures S40–S43 of the Supporting Information). To further confirm that bokeelamides function as siderophores, Fe^3+^-binding affinities and metal chelation selectivity studies were conducted on both compounds **1** and **2**. First, pFe^III^ values for compounds **1** and **2** were quantified using an ethylenediaminetetraacetic acid (EDTA) titration assay,^25^ which were determined to be 25.9 ± 0.2 and 25.7 ± 0.2, respectively (Figures S44 and S45 of the Supporting Information). These values are in the range of other amphiphilic lipopeptide siderophores.^18^ In addition, both compounds **1** and **2** are capable of chelating Fe^3+^ and Ga^3+^ but not Zn^2+^, Cu^2+^, or Co^2+^ (Figures S46 and S47 of the Supporting Information). When the metals are present in a mixture, there is a 6-fold selectivity for Fe^3+^-bokeelamide versus Ga^3+^-bokeelamide.

Environmental iron is largely insoluble, thereby limiting its bioavailability.^26^ Bacterial siderophores are secreted to scavenge Fe^3+^; however, only bacterial species with specific importers can uptake Fe^3+^-bound metabolites.^27^ Therefore, siderophores further limit Fe^3+^ availability, and by consequence, siderophores have been implicated in shaping host–microbe interactions by inhibiting growth of competing microorganisms.^25,26^ Given this precedent, we evaluated purified compounds **1**–**4** in a range of assays, including antibacterial (e.g., *Staphylococcus aureus* and *Escherichia coli*), antifungal (e.g., *Fusarium keritoplasticum*, *Aspergillus flavus*, and *Candida albicans*), antibiofilm, and red blood cell lysis assays. No activity was observed for any of the compounds at concentrations up to 50 μM (Figures S48–S53 of the Supporting Information). The lack of toxicity toward the various microbial pathogens suggests that compounds **1**–**4** may not serve a defensive role, but their ability to sequester Fe^3+^ could still shape the overall microbial community within the egg masses. Future work will be needed to fully understand the ecological role of bokeelamides within this system.

## Data Availability

The data underlying this study are available in the published article and its Supporting Information and GNPS at https://gnps.ucsd.edu/ProteoSAFe/status.jsp?task=6d7d6ca7584c41ee800a2b0888d49a52, MassIVE Repository under accession number MSV000094443 (DOI: 10.25345/C55D8NR8T) at https://massive.ucsd.edu/ProteoSAFe/dataset.jsp?accession=MSV000094443, NCBI under accession number JBHEGD010000000 at https://www.ncbi.nlm.nih.gov/nuccore/JBHEGD000000000, and NP-MRD under accession numbers NP0341802 (DOI: 10.57994/3288), NP0341803 (DOI: 10.57994/3289), NP0341804 (DOI: 10.57994/3290), and NP0341805 (DOI: 10.57994/3291) at https://np-mrd.org/.

## Supporting Information Available

NMR and MS data, Marfey’s analysis results, genomics data on *E. khazarica* EM133, and bioassay data (PDF)
