# Supplementary material for: Bokeelamides: Lipopeptides from Bacteria Associated with Marine Egg Masses
Source: Org Lett. 2024 Nov 1;26(45):9693–7. doi: 10.1021/acs.orglett.4c03470 (PMC11574851; doi:10.1021/acs.orglett.4c03470)
Supplement: Supplementary file 1 — ol4c03470_si_001.pdf [file ol4c03470_si_001.pdf]

Supporting Information

The Bokeelamides: Lipopeptides from bacteria associated with marine egg masses

Rose Campbell, Lois Kyei, Karla Piedl, Zheye Zhang, Ming Chen, Emily Mevers[*]

Department of Chemistry, Virginia Tech, Blacksburg, Virginia, USA, 24061

TABLE OF CONTENTS

Experimental Section

General Experimental Procedures....4
General Bacterial Culturing....4
GNPS Molecular Networks of Bacterial Extracts....4
HPLC Purification of Bokeelamides (**1 - 4**)....4
Synthesis of L- and D-erythro-βOH-Aspartic Acids....7
Acid Hydrolysis and Marfey's Analysis of Bokeelamides (**1 - 4**)....7
Sequencing, Assembly and Annotation of *Ectopseudomonas khazarica* EM133 genome....8
AntiSMASH Analysis of the Assembled Genome....8
Determining Iron Affinity (pFe^{3+}) for Bokeelamides A (**1**) and B (**2**)....8
Determining the Metal Selectivity for Bokeelamides A (**1**) and B (**2**)....9
Biofilm Inhibition and Antibacterial Assays with the Bokeelamides (**1 - 4**)....9
Antifungal Evaluation of the Bokeelamides (**1 - 4**)....9
Hemolytic Evaluation of the Bokeelamides (**1 - 4**)....9

Supplemental Figures and Tables

Figure S1: GNPS Network output....11
Figure S2: SIRIUS fragmentation tree of bokeelamide A (**1**)....12
Figure S3: SIRIUS fragmentation tree of bokeelamide B (**2**)....13
Figure S4: SIRIUS fragmentation tree of bokeelamide D (**4**)....14
Table S1: Bacterial strains represented in GNPS bokeelamide cluster....15
Figure S5: HPLC-MS chromatogram of EM133 fraction E....15
Figure S6: ^{1}H NMR spectrum of bokeelamide A (**1**) in d_6-DMSO (600 MHz)....15
Figure S7: ^{13}C NMR spectrum of bokeelamide A (**1**) in d_6-DMSO (125 MHz)....16
Figure S8: gHSQC spectrum of bokeelamide A (**1**) in d6-DMSO (600 MHz)....16
Figure S9: 5Hz HMBC spectrum of bokeelamide A (**1**) in d_6-DMSO (600 MHz)....17
Figure S10: H2BC spectrum of bokeelamide A (**1**) in d_6-DMSO (600 MHz)....17
Figure S11: TOCSY spectrum of bokeelamide A (**1**) in d_6-DMSO (600 MHz)....18
Figure S12: gCOSY spectrum of bokeelamide A (**1**) in d_6-DMSO (600 MHz)....18
Table S2: NMR data table for bokeelamide A (**1**)....19
Figure S13: ^{1}H NMR spectrum of bokeelamide B (**2**) in d_6-DMSO with 0.1% TFA (600 MHz)....20
Figure S14: ^{13}C NMR spectrum of bokeelamide B (**2**) in d_6-DMSO with 0.1% TFA (125 MHz)....20
Figure S15: gHSQC spectrum of bokeelamide B (**2**) in d_6-DMSO with 0.1% TFA (600 MHz)....21
Figure S16: HMBC spectrum of bokeelamide B (**2**) in d_6-DMSO with 0.1% TFA (600 MHz)....21
Figure S17: H2BC spectrum of bokeelamide B (**2**) in d_6-DMSO with 0.1% TFA (600 MHz)....22
Figure S18: TOCSY spectrum of bokeelamide B (**2**) in d_6-DMSO with 0.1% TFA (600 MHz)....22
Figure S19: gCOSY spectrum of bokeelamide B (**2**) in d_6-DMSO with 0.1% TFA (600 MHz)....23
Table S3: NMR data table for bokeelamide B (**2**)....24
Figure S20: ^{1}H NMR spectrum of bokeelamide C (**3**) in d_6-DMSO (600 MHz)....25
Figure S21: gHSQC spectrum of bokeelamide C (**3**) in d_6-DMSO (600 MHz)....25
Figure S22: HMBC spectrum of bokeelamide C (**3**) in d_6-DMSO (600 MHz)....26
Figure S23: H2BC spectrum of bokeelamide C (**3**) in d_6-DMSO (600 MHz)....26
Figure S24: TOCSY spectrum of bokeelamide C (**3**) in d_6-DMSO (600 MHz)....27
Figure S25: dqfCOSY spectrum of bokeelamide C (**3**) in d_6-DMSO (600 MHz)....27
Table S4: NMR data table for bokeelamide C (**3**)....28
Figure S26: ^{1}H NMR spectrum of bokeelamide D (**4**) in d_6-DMSO (600 MHz)....29
Figure S27: gHSQC spectrum of bokeelamide D (**4**) in d_6-DMSO (600 MHz)....29

Figure S28: HMBC spectrum of bokeelamide D (**4**) in d_6-DMSO (600 MHz) 30
Figure S29: H2BC spectrum of bokeelamide D (**4**) in d_6-DMSO (600 MHz) 30
Figure S30: TOCSY spectrum of bokeelamide D (**4**) in d_6-DMSO (600 MHz)................................. 31
Figure S31: dqfCOSY spectrum of bokeelamide D (**4**) in d_6-DMSO (600 MHz).............................31
Table S5: NMR data table for bokeelamide D (**4**) 32
Figure S32: Fragmentation of bokeelamide G (**7**) 33
Figure S33: Fragmentation of bokeelamide H (**8**) 33
Figure S34: Fragmentation of bokeelamide I (**9**)...33
Scheme S1: Synthetic scheme for L-*erythro*-βOH-Aspartic acid 34
Scheme S2: Synthetic scheme for D-*erythro*-βOH-aspartic acid 34
Figure S35. Marfey's analysis of bokeelamide A (**1**) 35
Figure S36. Marfey's analysis of bokeelamide B (**2**) 36
Figure S37. Marfey's analysis of bokeelamide C (**3**) 37
Figure S38. Marfey's analysis of bokeelamide D (**4**) 38
Table S6: Tools used by SeqCenter for genome assembly and annotation 39
Figure S39: Genomic phylogram of *E. khazarica* EM 133 39
Table S7: Genes within the *bka* biosynthetic gene cluster and their predicted product 40
Table S8: Media Recipes 41
Figure S40: EIC of apo- and Fe-bokeelamide A (**1**)...41
Figure S41: EIC of apo- and Fe-bokeelamide B (**2**)...41
Figure S42: EIC of apo- and Fe-bokeelamide C (**3**)...42
Figure S43: EIC of apo- and Fe-bokeelamide D (**4**)...42
Figure S44: Competition titration of EDTA against bokeelamide A (**1**)..42
Figure S45: Competition titration of EDTA against bokeelamide B (**2**)..42
Figure S46: Metal chelation selectivity of bokeelamide A (**1**)..43
Figure S47: Metal chelation selectivity of bokeelamide B (**2**) 43
Figure S48: Antibacterial and biofilm inhibition assays against *S. aureus* 44
Figure S49: Antibacterial and biofilm inhibition assays against *P. aeruginosa*.................................44
Figure S50: Bokeelamides A - D (**1 - 4**) against *Candida albicans* 44
Figure S51: Bokeelamides A - D (**1 - 4**) against *Aspergillus flavus*...45
Figure S52: Bokeelamides A - D (**1 - 4**) against *Fusarium keritoplasticum*....................................... 45
Figure S53: Relative percent hemolysis of bokeelamides A - D (**1 - 4**)...45
References.. 46

Experimental Section

General Experimental Procedures: Optical rotation data was recorded on a JASCO P-2000 polarimeter. CD and UV/Vis spectra were recorded on a JASCO J-815 CD Spectrometer. NMR spectra were recorded in *d*$_6$-DMSO with the residual solvent peak as an internal standard (δ_C 39.5, δ_H 2.50) on a Bruker Avance III 600 MHz instrument equipped with a triple resonance inverse (CP-TCI) Prodigy N_2 cooled CryoProbe (^{1}H 600 MHz; ^{13}C 150 MHz) and a Bruker Avance II 500 MHz instrument equipped with a CPBBO Prodigy N_2 cooled CryoProbe (^{1}H 500 MHz; ^{13}C 125 MHz). LR-LCMS data was obtained using an Agilent 1200 series HPLC system equipped with a photo-diode array detector and a Thermo LTQ mass spectrometer. HR-ESIMS and HR-MSMS were carried out using an Agilent 6530 Q-TOF equipped with a 1290 Infinity II UPLC system or a Thermo Orbitrap Exploris 120 Mass Spectrometer under direct injection. HPLC purifications were carried out using Agilent 1200 series or 1260 Infinity II HPLC systems equipped with a photodiode array detector. All solvents were of HPLC quality. All bacterial work was carried out in a SterilGardIII Advance sterile hood. Microplate readings were taken on a BioTek Cytation 3 Cell Imaging Multi-Mode Reader. Samples were centrifuged in an Eppendorf Centrifuge 5810R or 5430R. Structural assignments were made with additional information from gHSQC, gH2BC, TOCSY, and gHMBC experiments.

General Bacterial Culturing: All bacteria extracted for GNPS molecular networking were grown in Yeast Extract Malt Extract (YEME) broth, R2A broth, and A Media broth in artificial seawater (**Table S8**) and extracted as described in Kyei et al.[1] Unless otherwise specified, *Ectopseudomonas khazarica* EM133 was cultured in YEME with seawater or on ISP2 with seawater (YEME media +18 g/L agar) at 30ºC. All fungi used in antifungal assays were cultured in Yeast Peptone Dextrose (YPD) medium with DI water (*Candida albicans* and *Aspergillus flavus*) or with artificial seawater (*Fusarium keritoplasticum*), and bacterial pathogens (*Staphylococcus aureus* and *Pseudomonas aeruginosa*) were cultured in Luria-Bertani (LB) medium. Recipes for all media can be found in **Table S8**.

GNPS Molecular Networks of Bacterial Extracts: All moon snail egg mass-associated HR-MSMS data (123 bacterial extract fraction files, three egg mass extract files) were converted to mzML format using MSConvert and uploaded to the GNPS platform (http://gnps.ucsd.edu) for Classical Molecular Networking using workflow METABOLOMICS-SNETS-V2 (version release_30).[2] The data was filtered by removing all MS/MS fragment ions within ±17 Da of the precursor *m/z*. MS/MS spectra were window filtered by choosing only the top 6 fragment ions in the ± 50Da window throughout the spectrum. The precursor ion mass tolerance was set to 0.02 Da and MS/MS fragment ion tolerance to 0.02 Da. A network was then created where edges were filtered to have a cosine score above 0.7 and more than 6 matched peaks. Further, edges between two nodes were kept in the network if and only if each of the nodes appeared in each other's respective top 10 most similar nodes. Finally, the maximum size of a molecular family was set to 100, and the lowest scoring edges were removed from molecular families until the molecular family size was below this threshold. The spectra in the network were then searched against GNPS' spectral libraries. The library spectra were filtered in the same manner as the input data. All matches kept between network spectra and library spectra were required to have a score above 0.7 and at least 6 matched peaks. The resulting molecular network was imported to Cytoscape (**Fig. S1**).

*HPLC Purification of Bokeelamides (**1** - **4**):* Compounds **1** - **4** were isolated from the semi-crude fraction (fraction E; 60% MeOH/H_2O) produced by *E. khazarica* EM133 by reversed-phase column synergi Hydro-RP (Phenomenex, 4 µm, 250 × 10 mm) with DAD detector using the solvent system (phase A, H_2O+0.1% FA; phase B, CH_3CN+0.1% FA): 0-5 min 30% phase B; 5.01-35 min 30%-55% phase B; flow rate at 3 mL/min, tR = 22.3 min **(2),** 26.0 min **(1),** 33.5 min (**3**), and 29.0 min (**4**). Compound **4** was re-purified by reversed-phase column synergi Hydro-RP (Phenomenex, 4 µm, 250 x 10 mm) with DAD detector using the solvent system (phase A, H_2O+0.1% FA; phase B, CH_3CN+0.1% FA): 0-5 min 38% phase B; 5.01-36.1 min 38%-42% phase B; flow rate at 3 mL/min, tR = 32.5 min (**4**).

Bokeelamide A (1): pale yellow amorphous solid; $[\alpha]^{23}_D$ +16.6 (*c* 7.7, 50% CH_3CN/H_2O); UV (50% CH_3CN/H_2O) λ_{max} (logε) 205 (4.28) nm; ^{1}H NMR (d_6-DMSO, 600 MHz): δ 8.28 (m, 1H), 8.27 (m, 1H), 8.07 (d, 1H, J = 8.4 Hz), 7.81 (d, 1H, J = 7.8 Hz), 7.77 (d, 1H, J = 6.3 Hz), 5.31 (brs, 1H), 5.14, (brs, 1H), 5.14 (brs, 1H), 4.62 (m, 1H), 4.47 (brs, 1H), 4.32 (m, 1H), 4.28 (m, 1H), 4.25 (m, 1H), 4.21 (m, 1H), 3.64 (m, 2H), 3.57 (m, 2H), 3.54 (m, 1H), 3.46 (m, 2H), 3.41 (brs, 1H), 2.11 (tt, 2H, J = 7.4, 6.7 Hz), 1.99 (s, 3H), 1.90 (m, 1H), 1.89 (m, 1H), 1.85 (m, 1H), 1.67 (q, 1H, J = 10.6 Hz), 1.50 (m, 2H), 1.49 (m, 2H), 1.44 (m, 2H), 1.25 (m, 2H), 1.22 (m, 14H), 0.84 (t, 3H, J = 7.1 Hz). ^{13}C{1H} NMR (d_6-DMSO, 125 MHz) δ 173.8, 173.2, 172.3, 170.4, 169.8, 169.7, 169.6, 165.1, 70.4, 62.0, 61.6, 56.1, 55.9, 55.2, 53.4, 51.3, 49.8, 46.5, 34.9, 31.4, 29.2, 29.1, 29.0, 28.9, 28.8, 28.7, 28.3, 27.3, 25.2, 22.7, 22.2, 20.5, 20.4, 14.1; ESI MS/MS (Orbitrap) *m/z* 772.4077 ($C_{34}H_{58}N_7O_{13}$), 754.4005 ($C_{34}H_{56}N_7O_{12}$), 736.3858 ($C_{34}H_{54}N_7O_{11}$), 660.3449 ($C_{29}H_{50}N_5O_{12}$), 642.3332 ($C_{29}H_{48}N_5O_{11}$), 632.3480 ($C_{28}H_{50}N_5O_{11}$), 624.3223 ($C_{29}H_{46}N_5O_{10}$), 614.3410 ($C_{28}H_{48}N_5O_{10}$), 596.3272 ($C_{28}H_{46}N_5O_9$), 573.3121 ($C_{26}H_{45}N_4O_{10}$), 555.3011 ($C_{26}H_{43}N_4O_9$), 545.3175 ($C_{25}H_{45}N_4O_9$), 537.2898 ($C_{26}H_{41}N_4O_8$), 527.3066 ($C_{25}H_{43}N_4O_8$), 486.2798 ($C_{23}H_{40}N_3O_8$), 468.2696 ($C_{23}H_{38}N_3O_7$), 436.1685 ($C_{15}H_{26}N_5O_{10}$), 418.1571 ($C_{15}H_{24}N_5O_9$), 400.1462 ($C_{15}H_{22}N_5O_8$), 355.2584 ($C_{19}H_{35}N_2O_4$), 313.2483 ($C_{17}H_{33}N_2O_3$), 306.0924 ($C_{10}H_{16}N_3O_8$), 305.1449 ($C_{11}H_{21}N_4O_6$), 295.2383 ($C_{17}H_{31}N_2O_2$), 288.0814 ($C_{10}H_{14}N_3O_7$), 218.1138 ($C_8H_{16}N_3O_4$), 200.1027 ($C_8H_{14}N_3O_3$), 131.0813 ($C_5H_{11}N_2O_2$), 114.0547 ($C_5H_8NO_2$), 86.0599 (C_4H_8NO); HRMS (ESI) m/z: $[M + H]^+$ Calcd for $C_{34}H_{60}N_7O_{14}$ 790.4198; Found 790.4198.

Bokeelamide B (2): pale yellow amorphous solid; $[\alpha]^{23}_D$ +19.4 (*c* 6.8, 50% CH_3CN/H_2O); UV (50% CH_3CN/H_2O) λ_{max} (logε) 209 (4.30) nm; ^{1}H NMR (d_6-DMSO, 600 MHz): δ 8.33 (m, 1H), 8.32 (m, 1H), 8.09 (d, 1H, J = 8.4 Hz), 7.80 (d, 1H, J = 8.6 Hz), 7.79 (d, 1H, J = 8.3 Hz), 5.33 (m, 1H), 5.30 (m, 1H), 4.65 (dd, 1H, J = 9.1, 2.3 Hz), 4.55 (d, 1H, J = 2.3 Hz), 4.33 (m, 1H), 4.27 (m, 1H), 4.25 (m, 1H), 4.18 (m, 1H), 3.63 (m, 1H), 3.58 (d, 1H, J = 5.6 Hz), 3.48 (m, 1H), 3.46 (m, 2H), 3.43 (m, 1H), 2.20 (t, 1H, J = 7.0 Hz), 2.12 (m, 1H), 1.96 (m, 2H), 1.98 (m, 5H), 1.90 (m, 1H), 1.89 (m, 2H), 1.68 (m, 1H), 1.63 (m, 1H), 1.53 (m, 2H), 1.50 (m, 2H), 1.49 (m, 1H), 1.27 (m, 2H), 1.25 (m, 2H), 1.23 (m, 4H). ^{13}C{1H} NMR (d_6-DMSO, 150 MHz) δ 173.6, 172.3, 172.0, 170.4, 170.0, 169.8, 169.5, 164.9, 130.2, 129.1, 71.1, 61.9, 61.6, 56.1, 55.4, 55.2, 53.5, 51.2, 49.8, 46.2, 34.6, 29.2 (x2), 28.8, 28.4, 27.2, 26.7, 26.3, 25.3, 22.9, 22.2, 20.4, 20.4, 14.1; ESI MS/MS (Orbitrap) *m/z* 770.3953 ($C_{34}H_{56}N_7O_{13}$), 752.3832 ($C_{34}H_{54}N_7O_{12}$), 734.3690 ($C_{34}H_{52}N_7O_{11}$), 658.3273 ($C_{29}H_{48}N_5O_{12}$), 640.3198 ($C_{29}H_{46}N_5O_{11}$), 630.3342 ($C_{28}H_{48}N_5O_{11}$), 622.3090 ($C_{29}H_{44}N_5O_{10}$), 612.3224 ($C_{28}H_{46}N_5O_{10}$), 594.3133 ($C_{28}H_{44}N_5O_9$), 571.2987 ($C_{26}H_{43}N_4O_{10}$), 553.2871 ($C_{26}H_{41}N_4O_9$), 543.3012 ($C_{25}H_{43}N_4O_9$), 535.2770 ($C_{26}H_{39}N_4O_8$), 525.2920 ($C_{25}H_{41}N_4O_8$), 484.2651 ($C_{23}H_{38}N_3O_8$), 466.2539 ($C_{23}H_{36}N_3O_7$), 436.1658 ($C_{15}H_{26}N_5O_{10}$), 418.1572 ($C_{15}H_{24}N_5O_9$), 400.1461 ($C_{15}H_{22}N_5O_8$), 353.2435 ($C_{19}H_{33}N_2O_4$), 311.2332 ($C_{17}H_{31}N_2O_3$), 306.0925 ($C_{10}H_{16}N_3O_8$), 305.1449 ($C_{11}H_{21}N_4O_6$), 293.2224 ($C_{17}H_{29}N_2O_2$), 288.0822 ($C_{10}H_{14}N_3O_7$), 218.1138 ($C_8H_{16}N_3O_4$), 200.1027 ($C_8H_{14}N_3O_3$), 131.0813 ($C_5H_{11}N_2O_2$), 113.0708 ($C_5H_9N_2O$); HRMS (ESI) m/z: $[M + H]^+$ Calcd for $C_{34}H_{58}N_7O_{14}$ 788.4042; Found 788.4038.

Bokeelamide C (3): pale yellow amorphous solid; $[\alpha]^{24}_D$ +26.3 (*c* 1.2, 50% CH_3CN/H_2O); UV (50% CH_3CN/H_2O) λ_{max} (logε) 208 (4.20) nm; ^{1}H NMR (d_6-DMSO, 600 MHz): δ 8.12 (d, 1H, J = 8.3 Hz), 8.06 (m, 1H), 7.97 (d, 1H, J = 8.0 Hz), 7.81 (d, 1H, J = 7.8 Hz), 7.63 (d, 1H, J = 6.7 Hz), 5.10 (brs, 1H), 4.53 (dd, 1H, J = 8.8, 1.8 Hz), 4.32 (m, 1H), 4.29 (m, 1H), 4.23 (m, 1H), 4.21 (m, 1H), 4.09 (s, 1H), 3.72 (m, 1H), 3.65 (m, 1H), 3.59 (m, 1H), 3.55 (m, 1H), 3.45 (m, 1H), 3.06 (m, 1H), 2.15 (t, 1H, J = 7.4 Hz), 2.09 (t, 1H, J = 7.2 Hz), 2.01 (s, 3H), 1.91 (m, 1H), 1.89 (m, 1H), 1.85 (m, 1H), 1.66 (dq, 1H, J = 10.9, 1.9 Hz), 1.59 (m, 1H), 1.45 (m, 1H), 1.44 (m, 1H), 1.43 (m, 1H), 1.36 (m, 1H), 1.25 (m, 2H), 1.23 (m, 16H), 1.22 (m, 2H), 0.85 (t, 3H, J = 6.9 Hz). ^{13}C NMR (extrapolated from HSQC and HMBC; d_6-DMSO) δ 172.9, 171.5, 170.9, 170.0, 169.8, 169.7, 164.9, 71.5, 61.4, 61.3, 56.3, 55.5, 55.2, 52.4, 51.0, 49.5, 46.1, 34.6, 31.1, 28.3-28.8 (x8), 28.7, 27.0, 25.0, 21.9, 21.7, 20.4, 20.0, 13.7; ESI MS/MS (Q-TOF) *m/z* 800.4257 ($C_{36}H_{62}N_7O_{13}$), 782.4089 ($C_{36}H_{60}N_7O_{12}$), 688.3931 ($C_{31}H_{54}N_5O_{12}$), 670.3505 ($C_{31}H_{52}N_5O_{11}$), 658.3467 ($C_{30}H_{54}N_5O_{11}$), 601.3381 ($C_{28}H_{49}N_4O_{10}$), 583.3246 ($C_{28}H_{47}N_4O_9$), 565.2921 ($C_{28}H_{45}N_4O_8$), 514.3032

($C_{25}H_{44}N_3O_8$), 496.2985 ($C_{25}H_{42}N_3O_7$), 436.1570 ($C_{15}H_{26}N_5O_{10}$), 418.1524 ($C_{15}H_{24}N_5O_9$), 383.2819 ($C_{21}H_{39}N_2O_4$), 341.2752 ($C_{19}H_{37}N_2O_3$), 323.2673 ($C_{19}H_{35}N_2O_2$), 306.0996 ($C_{10}H_{16}N_3O_8$), 305.1339 ($C_{11}H_{21}N_4O_6$), 288.0804 ($C_{10}H_{14}N_3O_7$), 218.1080 ($C_8H_{16}N_3O_4$), 131.1090 ($C_5H_{11}N_2O_2$), 114.0517 ($C_5H_8NO_2$); HRMS (ESI) m/z: $[M + H]^+$ Calcd for $C_{36}H_{64}N_7O_{14}$ 818.4511; Found 818.4526.

*Bokeelamide D (**4**):* pale yellow amorphous solid; $[\alpha]^{24}_D$ +25.1 (*c* 1.4, 50% CH_3CN/H_2O); UV (50% CH_3CN/H_2O) λ_{max} (logε) 210 (4.29) nm; ^1H NMR (d_6-DMSO, 600 MHz): δ 8.22 (d, 1H, J = 8.3 Hz), 8.18 (s, 1H), 8.04 (d, 1H, J = 8.2 Hz), 7.81 (d, 1H, J = 8.0 Hz), 7.72 (d, 1H, J = 6.5 Hz), 5.33 (m, 1H), 5.31 (m, 1H), 5.11 (brs, 1H), 4.59 (dd, 1H, J = 8.8, 1.1 Hz), 4.32 (m, 1H), 4.27 (m, 1H), 4.26 (m, 1H), 4.24 (m, 1H), 3.65 (m, 2H), 3.61 (m, 1H), 3.59 (d, 2H, J = 5.6 Hz), 3.46 (m, 2H), 3.29 (m, 1H), 2.14 (q, 1H, J = 7.3 Hz), 2.11 (q, 1H, J = 7.3 Hz), 2.00 (s, 3H), 1.97 (m, 2H), 1.96 (m, 1H)), 1.91 (m, 2H), 1.90 (m, 2H), 1.67 (m, 1H), 1.54 (m, 1H), 1.49 (m, 1H), 1.47 (m, 1H), 1.46 (m, 1H), 1.43 (m, 1H), 1.28 (m, 2H), 1.25 (m, 2H), 1.24 (m, 8H), 0.85 (t, 3H, J = 6.8 Hz). ^{13}C NMR (extrapolated from HSQC and HMBC; d_6-DMSO) δ 173.3, 172.0, 171.9, 170.3, 170.1, 169.8, 169.7, 165.0, 129.5, 129.3, 70.9, 61.6, 61.3, 55.8, 55.6, 55.0, 52.8, 50.9, 49.5, 46.1, 34.5, 28.7, 28.3, 28.0, 28.0-30.9 (x3), 27.0, 26.4, 26.3, 24.8, 22.1, 21.9, 20.2, 20.0, 13.8; ESI MS/MS (Orbitrap) *m/z* 798.4232 ($C_{36}H_{60}N_7O_{13}$), 780.4120 ($C_{36}H_{58}N_7O_{12}$), 762.4045 ($C_{36}H_{56}N_7O_{11}$), 686.3629 ($C_{31}H_{52}N_5O_{12}$), 668.3506 ($C_{31}H_{50}N_5O_{11}$), 658.3631 ($C_{30}H_{52}N_5O_{11}$), 650.3414 ($C_{31}H_{48}N_5O_{10}$), 640.3541 ($C_{30}H_{50}N_5O_{10}$), 622.3421 ($C_{30}H_{48}N_5O_9$), 599.3276 ($C_{28}H_{47}N_4O_{10}$), 581.3168 ($C_{28}H_{45}N_4O_9$), 571.3355 ($C_{27}H_{47}N_4O_9$), 563.3070 ($C_{28}H_{43}N_4O_8$), 553.3227 ($C_{27}H_{45}N_4O_8$), 512.2966 ($C_{25}H_{42}N_3O_8$), 494.2874 ($C_{25}H_{40}N_3O_7$), 436.1684 ($C_{15}H_{26}N_5O_{10}$), 418.1572 ($C_{15}H_{24}N_5O_9$), 400.1462 ($C_{15}H_{22}N_5O_8$), 381.2728 ($C_{21}H_{37}N_2O_4$), 339.2635 ($C_{19}H_{35}N_2O_3$), 321.2533 ($C_{19}H_{33}N_2O_2$), 306.0926 ($C_{10}H_{16}N_3O_8$), 305.1449 ($C_{11}H_{21}N_4O_6$), 288.0816 ($C_{10}H_{14}N_3O_7$), 218.1138 ($C_8H_{16}N_3O_4$), 200.1027 ($C_8H_{14}N_3O_3$), 131.0814 ($C_5H_{11}N_2O_2$), 114.0548 ($C_5H_8NO_2$), 113.0709 ($C_5H_9N_2O$), 86.0599 (C_4H_8NO); HRMS (ESI) m/z: $[M + H]^+$ Calcd for $C_{36}H_{62}N_7O_{14}$ 816.4355; Found 816.4348.

*Bokeelamide E (**5**):* ESI MS/MS (Q-TOF) *m/z* 744.7589 ($C_{32}H_{54}N_7O_{13}$), 726.3596 ($C_{32}H_{52}N_7O_{12}$), 708.3435 ($C_{32}H_{50}N_7O_{11}$), 632.3046 ($C_{27}H_{46}N_5O_{12}$), 614.3044 ($C_{27}H_{44}N_5O_{11}$), 604.3050 ($C_{26}H_{46}N_5O_{11}$), 596.2550 ($C_{27}H_{42}N_5O_{10}$), 586.2927 ($C_{26}H_{44}N_5O_{10}$), 545.2790 ($C_{24}H_{41}N_4O_{10}$), 527.2770 ($C_{24}H_{39}N_4O_9$), 517.2828 ($C_{23}H_{41}N_4O_9$), 509.2784 ($C_{24}H_{37}N_4O_8$), 458.2412 ($C_{21}H_{36}N_3O_8$), 436.1690 ($C_{15}H_{26}N_5O_{10}$), 327.2211 ($C_{17}H_{31}N_2O_4$), 306.0896 ($C_{10}H_{16}N_3O_8$), 305.1440 ($C_{11}H_{21}N_4O_6$), 288.0706 ($C_{10}H_{14}N_3O_7$), 285.2064 ($C_{15}H_{29}N_2O_3$), 267.1919 ($C_{15}H_{27}N_2O_2$), 218.1165 ($C_8H_{16}N_3O_4$), 200.1057 ($C_8H_{14}N_3O_3$), 131.0761 ($C_5H_{11}N_2O_2$), 114.0456 ($C_5H_8NO_2$); HRMS (ESI) m/z: $[M + H]^+$ Calcd for $C_{32}H_{56}N_7O_{14}$ 762.3880; Found 762.3878.

*Bokeelamide F (**6**):* ESI MS/MS (Q-TOF) *m/z* 828.4565 ($C_{38}H_{66}N_7O_{13}$), 810.4527 ($C_{38}H_{64}N_7O_{12}$), 792.4361 ($C_{38}H_{62}N_7O_{11}$), 716.4066 ($C_{33}H_{58}N_5O_{12}$), 698.3783 ($C_{33}H_{56}N_5O_{11}$), 688.4082 ($C_{32}H_{58}N_5O_{11}$), 680.3696 ($C_{33}H_{54}N_5O_{10}$), 670.3803 ($C_{32}H_{56}N_5O_{10}$), 629.3852 ($C_{30}H_{53}N_4O_{10}$), 611.3557 ($C_{30}H_{51}N_4O_9$), 601.2253 ($C_{29}H_{53}N_4O_9$), 593.3360 ($C_{30}H_{49}N_4O_8$), 542.3403 ($C_{27}H_{48}N_3O_8$), 524.3477 ($C_{27}H_{46}N_3O_7$), 436.1690 ($C_{15}H_{26}N_5O_{10}$), 418.1407 ($C_{15}H_{24}N_5O_9$), 411.3206 ($C_{23}H_{43}N_2O_4$), 400.1360 ($C_{15}H_{22}N_5O_8$), 369.3184 ($C_{21}H_{41}N_2O_3$), 351.2868 ($C_{21}H_{39}N_2O_2$), 306.0795 ($C_{10}H_{16}N_3O_8$), 305.1440 ($C_{11}H_{21}N_4O_6$), 288.0804 ($C_{10}H_{14}N_3O_7$), 218.1080 ($C_8H_{16}N_3O_4$), 131.0761 ($C_5H_{11}N_2O_2$), 114.0517 ($C_5H_8NO_2$), 113.0645 ($C_5H_9N_2O$); HRMS (ESI) m/z: $[M + H]^+$ Calcd for $C_{38}H_{68}N_7O_{14}$ 846.4819; Found 846.4859.

*Bokeelamide G (**7**):* ESI MS/MS (Q-TOF) *m/z* 758.3726 ($C_{33}H_{56}N_7O_{13}$), 740.3684 ($C_{33}H_{54}N_7O_{12}$), 722.3639 ($C_{33}H_{52}N_7O_{11}$), 646.3300 ($C_{28}H_{48}N_5O_{12}$), 628.3174 ($C_{28}H_{46}N_5O_{11}$), 618.3329 ($C_{27}H_{48}N_5O_{11}$), 610.3033 ($C_{28}H_{44}N_5O_{10}$), 600.3226 ($C_{27}H_{46}N_5O_{10}$), 559.3020 ($C_{25}H_{43}N_4O_{10}$), 541.2822 ($C_{25}H_{41}N_4O_9$), 523.2678 ($C_{25}H_{39}N_4O_8$), 513.2858 ($C_{24}H_{41}N_4O_8$), 472.2660 ($C_{22}H_{38}N_3O_8$), 454.2466 ($C_{22}H_{36}N_3O_7$), 436.1570 ($C_{15}H_{26}N_5O_{10}$), 418.1642 ($C_{15}H_{24}N_5O_9$), 400.1360 ($C_{15}H_{22}N_5O_8$), 341.2327 ($C_{18}H_{33}N_2O_4$), 306.0896 ($C_{10}H_{16}N_3O_8$), 305.1440 ($C_{11}H_{21}N_4O_6$), 299.2323 ($C_{16}H_{31}N_2O_3$), 288.0804 ($C_{10}H_{14}N_3O_7$), 281.2168 ($C_{16}H_{29}N_2O_2$), 218.1080 ($C_8H_{16}N_3O_4$), 200.0975 ($C_8H_{14}N_3O_3$), 131.0761 ($C_5H_{11}N_2O_2$),

114.0517 ($C_5H_8NO_2$), 113.0706 ($C_5H_9N_2O$); HRMS (ESI) m/z: $[M + H]^+$ Calcd for $C_{33}H_{58}N_7O_{14}$ 776.4036; Found 776.4042.

*Bokeelamide H (**8**):* ESI MS/MS (Orbitrap) *m/z* 786.4037 ($C_{35}H_{60}N_7O_{13}$), 768.4126 ($C_{35}H_{58}N_7O_{12}$), 750.3901 ($C_{35}H_{56}N_7O_{11}$), 674.3607 ($C_{30}H_{52}N_5O_{12}$), 656.3443 ($C_{30}H_{50}N_5O_{11}$), 646.3606 ($C_{29}H_{52}N_5O_{11}$), 638.3340 ($C_{30}H_{48}N_5O_{10}$), 628.3477 ($C_{29}H_{50}N_5O_{10}$), 610.3488 ($C_{29}H_{48}N_5O_9$), 587.3210 ($C_{27}H_{47}N_4O_{10}$), 569.3146 ($C_{27}H_{45}N_4O_9$), 551.3095 ($C_{27}H_{43}N_4O_8$), 541.3136 ($C_{26}H_{45}N_4O_8$), 500.2952 ($C_{24}H_{42}N_3O_8$), 482.2851 ($C_{24}H_{40}N_3O_7$), 436.1688 ($C_{15}H_{26}N_5O_{10}$), 418.1573 ($C_{15}H_{24}N_5O_9$), 400.1425 ($C_{15}H_{22}N_5O_8$), 369.2759 ($C_{20}H_{37}N_2O_4$), 327.2648 ($C_{18}H_{35}N_2O_3$), 309.2535 ($C_{18}H_{33}N_2O_2$), 306.0921 ($C_{10}H_{16}N_3O_8$), 305.1438 ($C_{11}H_{21}N_4O_6$), 288.0799 ($C_{10}H_{14}N_3O_7$), 218.1131 ($C_8H_{16}N_3O_4$), 200.1017 ($C_8H_{14}N_3O_3$), 131.0819 ($C_5H_{11}N_2O_2$), 114.0542 ($C_5H_8NO_2$), 113.0716 ($C_5H_9N_2O$), 86.0602 (C_4H_8NO); HRMS (ESI) m/z: $[M + H]^+$ Calcd for $C_{35}H_{62}N_7O_{14}$ 804.4349; Found 804.4350.

*Bokeelamide I (**9**):* ESI MS/MS (Q-TOF) *m/z* 814.5039 ($C_{37}H_{64}N_7O_{13}$), 796.4746 ($C_{37}H_{62}N_7O_{12}$), 778.4062 ($C_{37}H_{60}N_7O_{11}$), 702.3834 ($C_{32}H_{56}N_5O_{12}$), 684.3679 ($C_{32}H_{54}N_5O_{11}$), 674.3791 ($C_{31}H_{56}N_5O_{11}$), 666.3934 ($C_{32}H_{52}N_5O_{10}$), 656.3750 ($C_{31}H_{54}N_5O_{10}$), 638.3527 ($C_{31}H_{52}N_5O_9$), 615.3602 ($C_{29}H_{51}N_4O_{10}$), 597.3514 ($C_{29}H_{49}N_4O_9$), 587.3660 ($C_{28}H_{51}N_4O_9$), 579.3331 ($C_{29}H_{47}N_4O_8$), 569.3490 ($C_{28}H_{49}N_4O_8$), 528.3213 ($C_{26}H_{46}N_3O_8$), 510.3047 ($C_{23}H_{38}N_3O_7$), 436.1690 ($C_{15}H_{26}N_5O_{10}$), 418.1407 ($C_{15}H_{24}N_5O_9$), 400.1360 ($C_{15}H_{22}N_5O_8$), 397.2979 ($C_{22}H_{41}N_2O_4$), 355.2886 ($C_{20}H_{39}N_2O_3$), 337.2794 ($C_{20}H_{37}N_2O_2$), 306.0896 ($C_{10}H_{16}N_3O_8$), 305.1440 ($C_{11}H_{21}N_4O_6$), 288.0804 ($C_{10}H_{14}N_3O_7$), 218.1080 ($C_8H_{16}N_3O_4$), 200.1057 ($C_8H_{14}N_3O_3$), 131.0761 ($C_5H_{11}N_2O_2$), 114.0517 ($C_5H_8NO_2$); HRMS (ESI) m/z: $[M + H]^+$ Calcd for $C_{37}H_{66}N_7O_{14}$ 832.4662; Found 832.4671.

Syntheses of L- and D-*erythro-βOH-Aspartic Acids:* Parallel preparations of both D- and L-*erythro*-βOH-aspartic acids followed Liu et al (**Scheme S1** and **S2**).[3] Briefly, thionyl chloride (0.73 mL, 10 mmol, 1.2 equiv.) was added dropwise to L- or D-diethyl tartrate (**10a** or **10b**, respectively; 1.77 g, 8.6 mmol, 1.0 equiv.) followed by slow addition of DMF (4 drops). The reactions were heated to 50 °C with an oil bath and stirred for 1 h, at which point they were cooled to rt and dried under reduced pressure. The mixtures were redissolved in DMF (5 mL), and sodium azide (1.68 g, 25.8 mmol, 3.0 equiv.) was added slowly. The resulting mixtures were stirred at rt for 16 h. Upon completion, the reactions were diluted with EtOAc (25 mL) and washed with water (3 x 30 mL) and brine (20 mL). The organic layer was dried over anhydrous sodium sulfate, filtered, and concentrated under reduced pressure to afford **11a** or **11b**, respectively. Crude products **11a - 11b** were used without further purification. Compounds **11a** or **11b** (1.16 g, 5 mmol, 1 equiv.) were resuspended in MeOH (15 mL), and Pd/C (116 mg, 10% on carbon) was added. The reaction mixtures were stirred under H_2 (g) at rt for 16 h. The resulting mixtures were filtered through Celite®, and the filtrates were concentrated under reduced pressure. The yields of **12a** and **12b** were 60% (616 mg) and 65% (667 mg), respectively. Product **12a** (43 mg, 0.19 mmol) or **12b** (104 mg, 0.45 mmol) was dissolved in MeOH (2.0 or 4.0 mL, respectively), then NaOH (3.125 M, 3.5 equiv.) was added and the reactions were stirred at rt for 1 h. Subsequently, the reactions were quenched to pH = ~8.5 using aqueous HCl (1 M) and dried under vacuum. Crude **13a** and **13b** products were used in derivatization with Marfey's reagent without further purification.

*Acid Hydrolysis and Marfey's Analysis of Bokeelamides (**1 - 4**):* The stereoconfigureation of amino acids present in bokeelamides A-D was determined using Marfey's analysis.[4] For **1 - 4**, 0.1 mg was treated with aqueous HCl (300 µL, 6 N) at 110 ºC in an oil bath for 16 h. The hydrolysate was then dried under air, dissolved in a solution of L-FDAA (1-fluoro-2-4-dinitrophenyl-5-L-alanine amide) in acetone (1.1 mL; 1 mg/mL), followed by the addition of aqueous $NaHCO_3$ (300 µL, 1 M). The mixture was heated to 40 ºC with a water bath and stirred for 1 h. The reaction was quenched by the addition of aqueous HCl (300 µL, 1 M) and dried under vacuum. The reaction product was resuspended in 100 µL 50% CH_3CN/H_2O for LR-LCMS analysis. Authentic standards were prepared similarly to the crude hydrolysate products but at

a scale of 1-2 mg starting material for commercial standards and approximately 5 mg for the crude *erythro*-*β*OH-Asp synthetic products. Derivatized standards and hydrolysate products were analyzed by reversed-phase column synergi Hydro-RP (Phenomenex, 4 µm, 4.6 × 250 mm) with DAD and MS (LTQ) detectors using the solvent system (phase A, H_2O+0.1% FA; phase B, CH_3CN+0.1% FA): 0-5 min 10% phase B; 5.01-90 min 10%-50% phase B; flow rate at 0.4 mL/min. The retention times of the authentic acid-L-FDAA derivatives were: L-Ser (51.5 min), D-Ser (52.5 min), tR = 83.6 min (L-Orn), 78.4 min (D-Orn), 51.8 min (L-*erythro*-*β*OH-Asp), 49.5 min (D-*erythro*-*β*OH-Asp), 42.1 min (L-*threo*-*β*OH-Asp), 40.6 min (D-*threo*-*β*OH-Asp). Extracted ion chromatograms (EIC) were generated using smoothing with Gaussian 11 and plotted in GraphPad Prism version 10.2.0.

Sequencing, Assembly and Annotation of Ectopseudomonas khazarica EM133 genome: A single colony of *E. khazarica* EM133 was grown in 5 mL liquid culture to an OD_{600} of 2 (~10^9 cells). The dense liquid culture was centrifuged (4000 rpm; 10 min), the supernatant decanted, and the cell pellet frozen at -20ºC. DNA extraction, sequencing, and assembly took place at SeqCenter (Pittsburgh, PA, USA). DNA was sequenced using 600 Mbp Nanopore technology on an Oxford Nanopore sequencer. Porechop[5] was used to trim the residual adapter sequence from the Oxford Nanopore Technology (ONT) reads that may have been missed during base-calling and demultiplexing. *De novo* genome assemblies were generated from the ONT read data with Flye[6] under the nano-hq (ONT high-quality reads) model. Additional Flye options initiate the assembly by first using reads longer than an estimated N50 based on a genome size of 6Mbp. Assembled contigs were evaluated for circularization via Circulator.[7] Assembly annotation was then performed with Bakta[8] using the Bakta v5 database. Finally, assembly statistics were recorded with QUAST (**Table S6**).[9] Species-level identification was accomplished by submission of the assembled genome to the Type (Strain) Genome Server (TYGS).[10]

AntiSMASH Analysis of the Assembled Genome: The assembled genome was submitted for analysis by AntiSMASH 7.1.0,[11] and the output was searched for non-ribosomal peptide synthetase (NRPS) gene clusters. The biosynthetic gene cluster (BGC) containing five NRPS modules that most closely matched that for azotobactin D (66% genes showed similarity) was investigated further. Greater insight was gained on each protein within this BGC by annotating the genome in Rapid Annotation using Subsystem Technology[12] and performing BLASTp alignment searches. The BGC was manually trimmed based on function related to bokeelamides, and all proteins were summarized in **Table S7.** Specificity of each adenylation domain was verified in the PKS/NRPS Analysis Website (https://nrps.igs.umaryland.edu/).[13]

*Determining Iron Affinity (pFe^{3+}) for Bokeelamides A (**1**) and B (**2**):* Ethylenediaminetetraacetic acid (EDTA) competition assays were performed in triplicate as previously described.[14] In brief, **1** and **2** (10 µmol) each were incubated with $FeCl_3$ (10 eq., 100 µmol, 16.2 mg) in 25% CH_3CN/H_2O at rt for 2 h, then purified with a C18 SPE (1 g) column by washing with H_2O and eluting with MeOH. Purified Fe-bokeelamide was dried down, weighed, then resuspended to 0.4 mM in 50% CH_3CN/H_2O. Stock solutions of EDTA (0.2-200 mM) were prepared in H_2O, then 25 µL of each stock was combined with 25 µL Fe^{3+}-bokeelamide stock and 50 µL HEPES buffer for final concentrations of 0.1 mM Fe^{3+}-**1** or Fe^{3+}-**2**, 0.05-50 mM EDTA, 10 mM HEPES, 0.1 M KCl, pH = 7.4. Samples were incubated at rt for 24 h, then analyzed using LR-LCMS with a reversed-phase C8 column (Kinetex, 5 µm, 3.0 × 100 mm, 100 Å) with DAD and MS (LTQ) detectors using the solvent system (phase A, H_2O+0.1% FA; phase B, CH_3CN+0.1% FA): 0-3 min 10% phase B; 3.01-14 min 10%-90% phase B; flow rate at 0.3 mL/min. Concentrations of apo- and Fe^{3+}-bokeelamide and Fe^{3+}-EDTA were calculated using the area under the curve (AUC) of apo- and Fe^{3+}-bokeelamide traces and log-ratios of the apo- vs Fe- forms were plotted. The ΔpFe^{3+} of **1** and **2** relative to EDTA (23.42) were determined from the x-intercept of the best fit line,[15] and pFe^{3+} was determined by averaging each replicate's pFe^{3+}.

*Determining the Metal Selectivity for Bokeelamides A (**1**) and B (**2**):* The apo- forms of **1** and **2** were freshly purified using HPLC methods described above and resuspended to 2 mM in 50% CH_3CN/H_2O. Stock solutions of 20 mM $CoSO_4$, $CuSO_4$, $FeCl_3$, $GaCl_3$, and $ZnCl_2$ were prepared in H_2O. Apo-**1** or -**2** (100 µL) was combined with either 100 µL one metal solution (single-metal samples) or 20 µL each metal solution (mix sample) for final concentrations of 1 mM apo-bokeelamide and 10 mM metal in each sample. After incubation at rt for 24 h, samples were analyzed using LR-LCMS with a reversed-phase C8 column (Kinetex, 5 µm, 3.0 × 100 mm) with DAD and MS (LTQ) detectors using the solvent system (phase A, H_2O+0.1% FA; phase B, CH_3CN+0.1% FA): 0-3 min 10% phase B; 3.01-14 min 10%-90% phase B; flow rate at 0.3 mL/min. Extracted ion chromatogram (EICs) of the apo- and metal-bokeelamides were generated for each spectrum, smoothed with Gaussian 7, and plotted in GraphPad Prism version 10.2.0. In the mixed-metal samples, the AUC was calculated for each EIC to determine fold-change in chelation of Fe^{3+} over Ga^{3+}.

*Biofilm Inhibition and Antibacterial Assays with the Bokeelamides (**1** - **4**):* Analysis of antibiofilm and antibacterial activity was performed as previously described by Kyei et al.[1] Liquid cultures of *S. aureus* (ATCC BAA-2313) and *P. aeruginosa* ΔMexAB-OprM were grown overnight at 37 °C. Compounds **1-4** were prepared in DMSO at 2.5 and 0.625 mM stock concentrations. An aliquot (2 µL) of each stock was added to fresh 96-well plates in quadruplicate. The overnight cultures were diluted to a starting OD_{600} of 0.01 in M63 liquid broth (**Table S8**) and 98 µL was transferred to each well, including solvent control wells. Inoculated plates were incubated in a static incubator for 16 to 20 h at 37 °C. Bacterial growth was quantified by measuring absorbance at 600 nm to ascertain whether the compounds exhibited antibacterial properties. The plates were washed with water and dried (30–60 min) at rt to remove the planktonic cells. Adhered biofilms were stained with 115 µL of 0.1% (wt/vol) crystal violet (*aq*) and placed on an orbital shaker at low speed for 30 min, then the crystal violet-bound biofilms were solubilized into 130 µL of 30% acetic acid (*aq*). The solubilized material was transferred to new 96-well plates and absorbances (595 nm) measured. Raw absorbance readings were normalized to a vehicle control (negative control).

*Antifungal Evaluation of the Bokeelamides (**1** - **4**):* Compounds **1** - **4** were screened for antifungal activity against the human pathogens *C. albicans* and *A. flavus* (NRRL 1957) and against the bobtail squid egg mass pathogen *F. keritoplasticum* in technical triplicates.[16] Stock solutions of each compound were prepared in DMSO at 2.5 and 0.63 mM. Cultures of *C. albicans* were prepared in liquid and diluted to an initial OD_{600} of 0.0024; cultures of the spore-formers were prepared by diluting 6 µL of frozen spore stock in 6 mL sterile YPD media. An aliquot (4 µL) of each compound was transferred to a 96-well plate, followed by 196 µL prepared fungal culture for final compound concentrations of 50 and 12.5 µM. Both human pathogens were incubated statically at 37 ºC for 24 h, then settled cells were resuspended by pipetting and absorbance was read at 600 nm. *F. keritoplasticum* was incubated statically at 30ºC for 48 h, then observed for growth inhibition and photographed.

*Hemolytic Evaluation of the Bokeelamides (**1** - **4**):* Compounds **1** - **4** were screened for hemolytic activity at 12.5 and 50 µM in technical quadruplicates using defibrinated sheep blood (Lampire Biological Laboratories, Pipersville, PA) and following previously reported methods.[17] Briefly, the defibrinated sheep blood was centrifuged (4000 rpm, 10 min), then the plasma layer was aspirated and replaced with an equal volume of 150 mM NaCl solution in which the cells were then resuspended; this wash step was repeated three more times, once with NaCl solution and twice with phosphate buffer (PBS, pH = 7.4). Next, 1 mL of the erythrocytes was transferred to 49 mL of PBS and mixed gently. Cells were allowed to settle to ensure no cell lysis had occurred, then resuspended gently before use in remaining steps. Compounds **1** - **4** were resuspended to 1.25 and 5 mM in DMSO. To each sample well of a 96-well V-bottom plate were added 2 µL of the appropriate bokeelamide stock solution and 8 µL PBS. Compounds were then diluted with 190 µL prepared blood cell mixture for final concentrations of 12.5 and 50 µM. Negative controls were prepared using 10 µL PBS, and positive controls were prepared with 10 µL 20%

Triton X-100 solution. The 96-well plate was incubated at 37ºC for an hour, then centrifuged (4000 rpm, 10 min). Finally, 100 µL of the supernatant from each well was transferred to a 96-well flat-bottom plate and absorbance was measured at 415 nm. Data were graphed using GraphPad Prism 10.2.0. Mean values are reported with the standard deviation representing the combination of the four replicates.

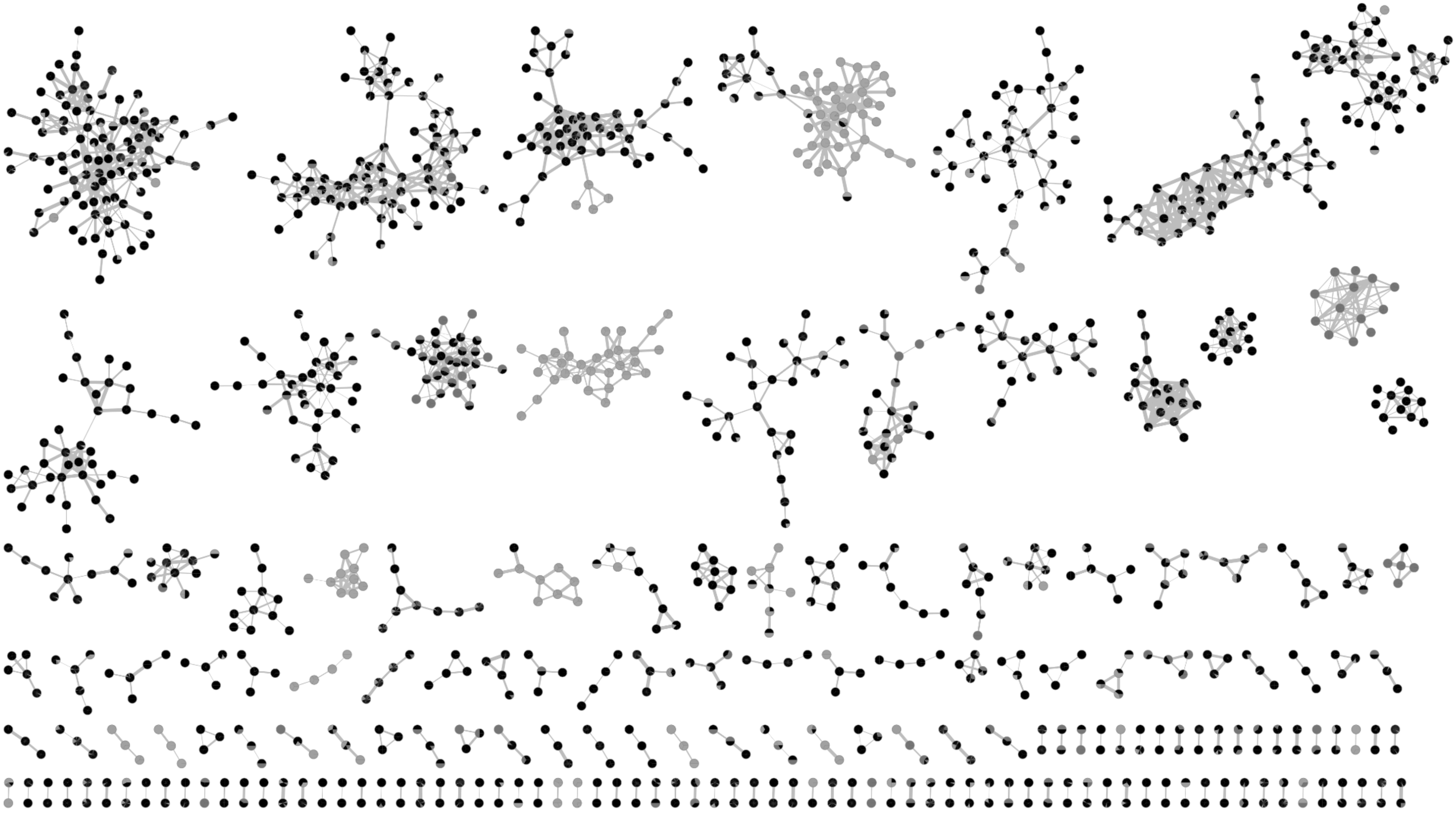

Figure S1: GNPS Network output, processed in Cytoscape, excluding single nodes

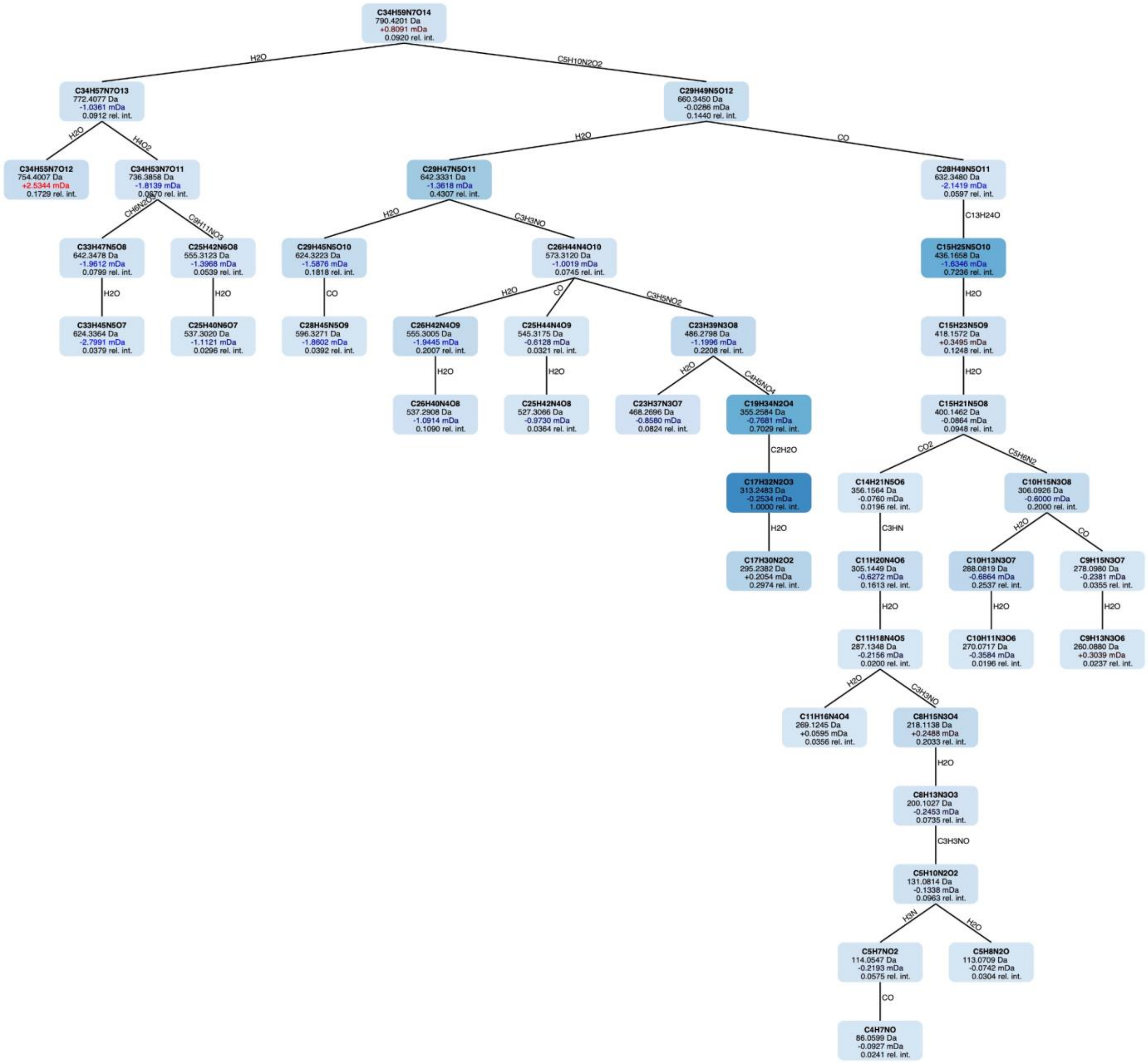

Figure S2: SIRIUS fragmentation tree of bokeelamide A (**1**), showing sequential neutral losses. Shade of blue indicates relative intensity of fragment peak, with darker shades indicating more intense peak.

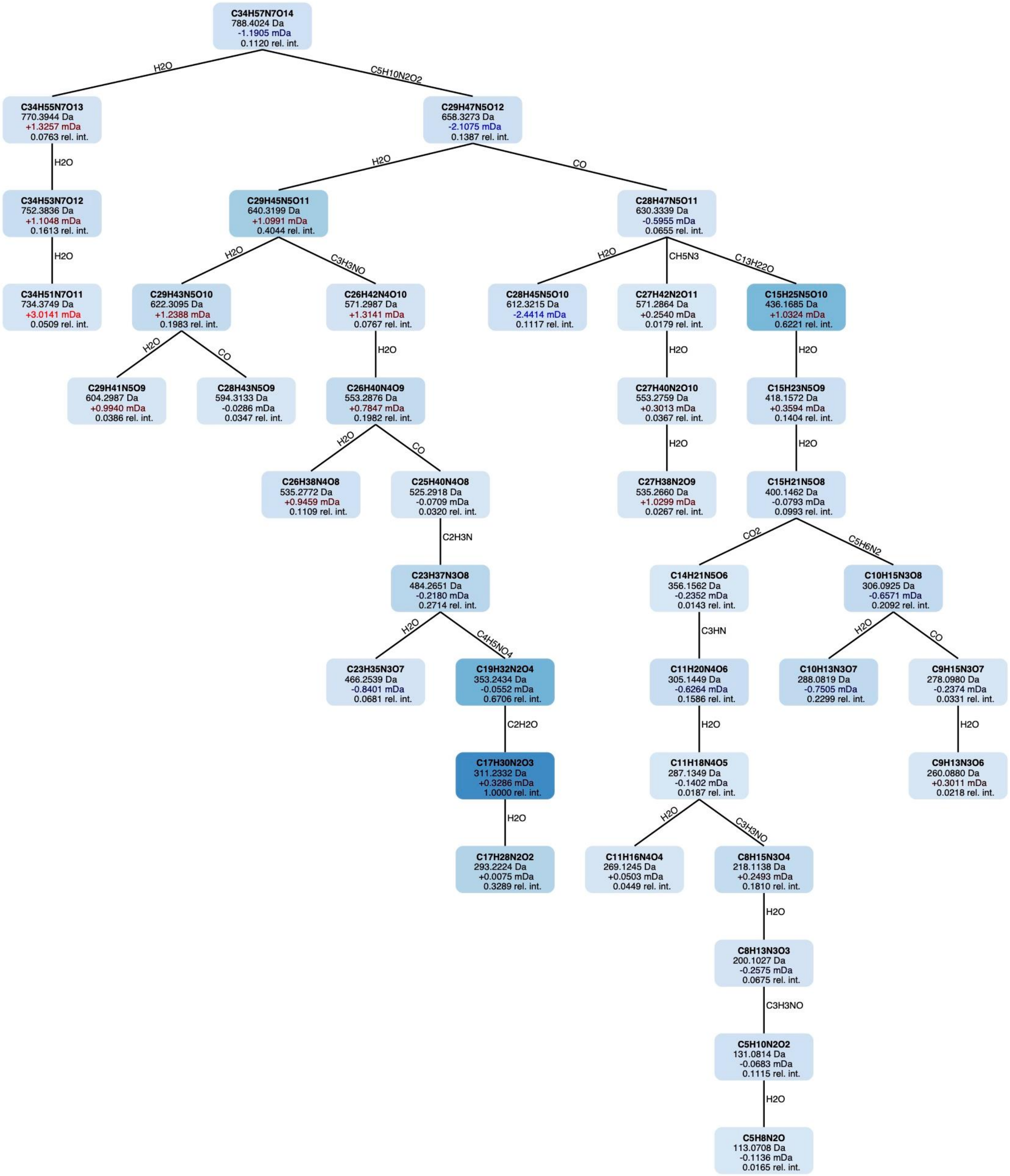

Figure S3: SIRIUS fragmentation tree of bokeelamide B (**2**), showing sequential neutral losses. Shade of blue indicates relative intensity of fragment peak, with darker shades indicating more intense peak.

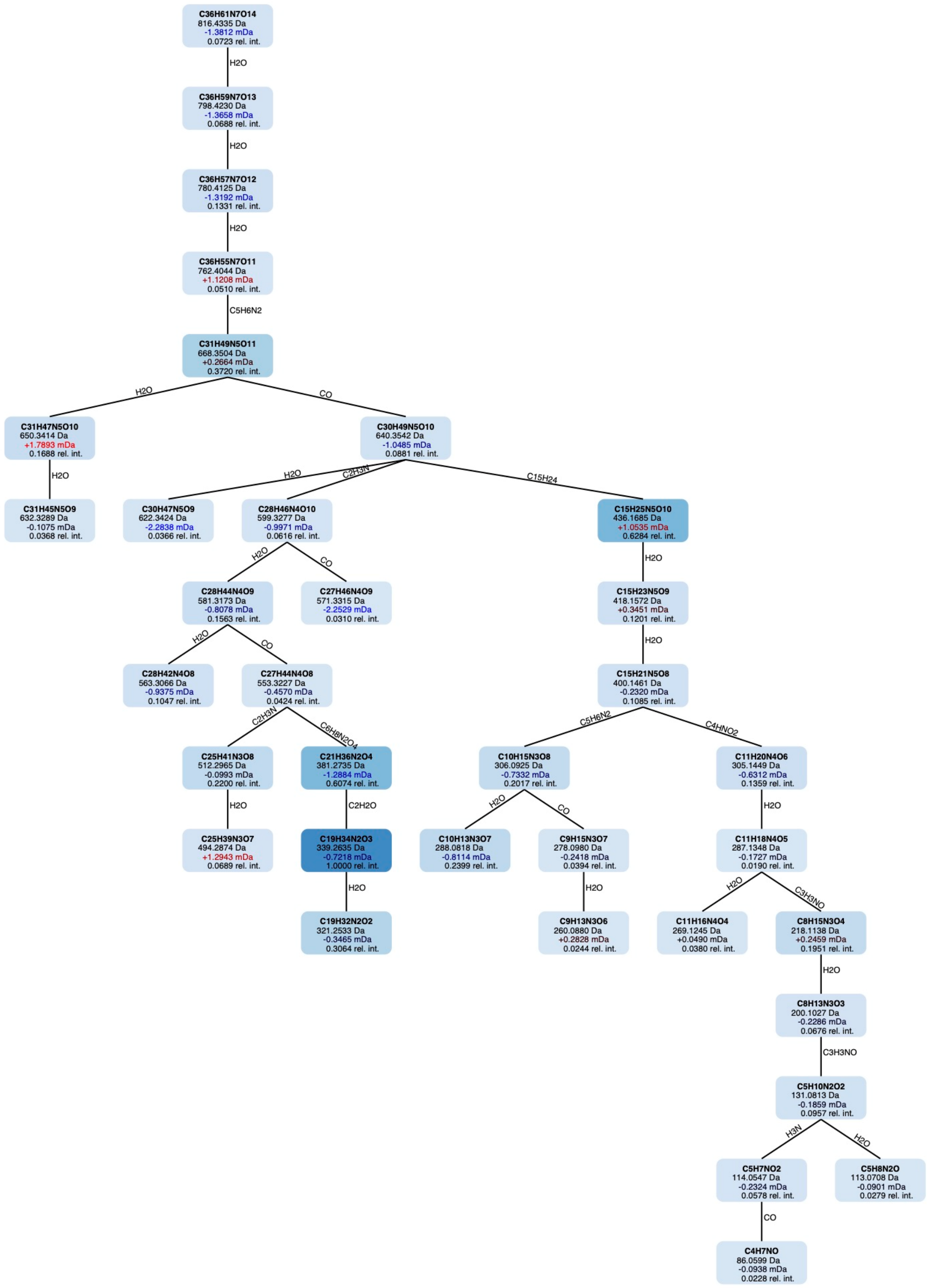

Figure S4: SIRIUS fragmentation tree of bokeelamide D (**4**), showing sequential neutral losses. Shade of blue indicates relative intensity of fragment peak, with darker shades indicating more intense peak.

Table S1: Bacterial strains represented in GNPS bokeelamide cluster, identifying producing strains

Strain ID	Bacteria Taxonomy	Bokeelamides produced
EM 133	*Ectopseudomonas khazarica*	A-I
EM 143	*Ectopseudomonas* sp.	A-D, F-I
EM 91	*Bacillus* sp.	A, B, D, H, I
EM 135	*Pseudoalteromonas* sp.	A, B, D, E, G, H
EM 103	*Vibrio* sp.	A-D

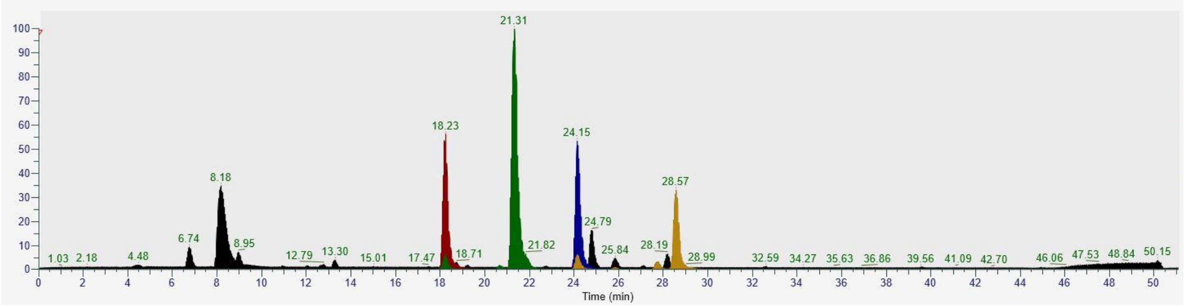

Figure S5: HPLC-MS chromatogram of EM133 fraction E showing major compounds **1** (green, 21.31 min), **2** (red, 18.23 min), **3** (yellow, 28.57 min), and **4** (blue, 24.15 min) that were targeted for isolation.

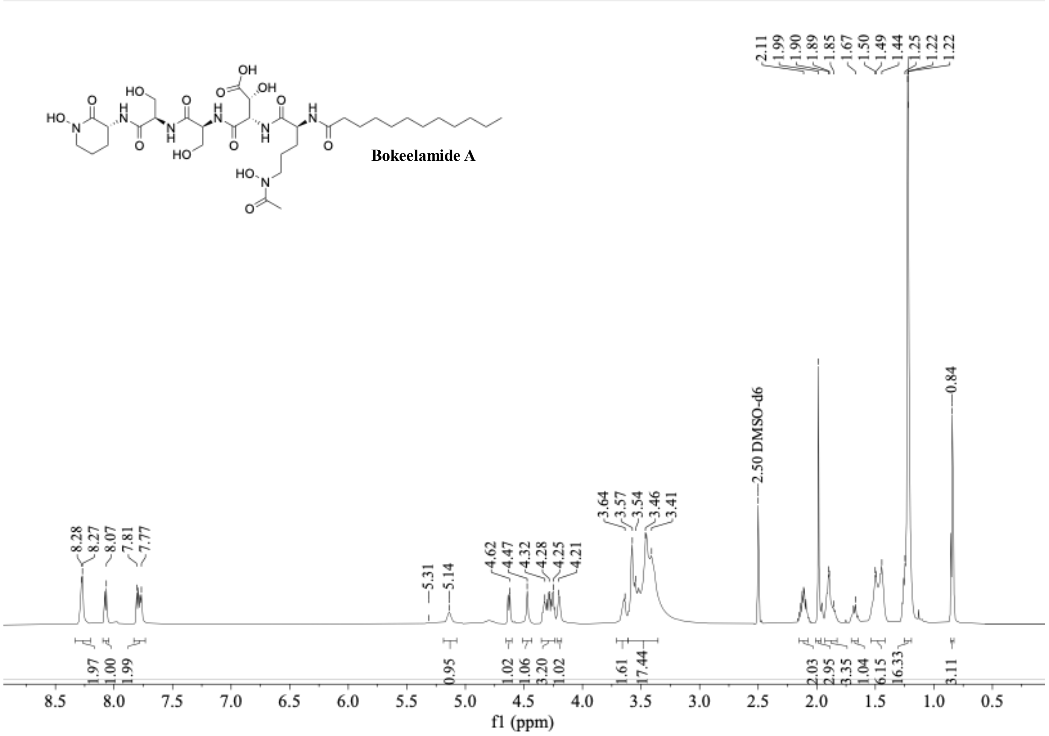

Figure S6: ^{1}H NMR spectrum of bokeelamide A (**1**) in d_6-DMSO (600 MHz)

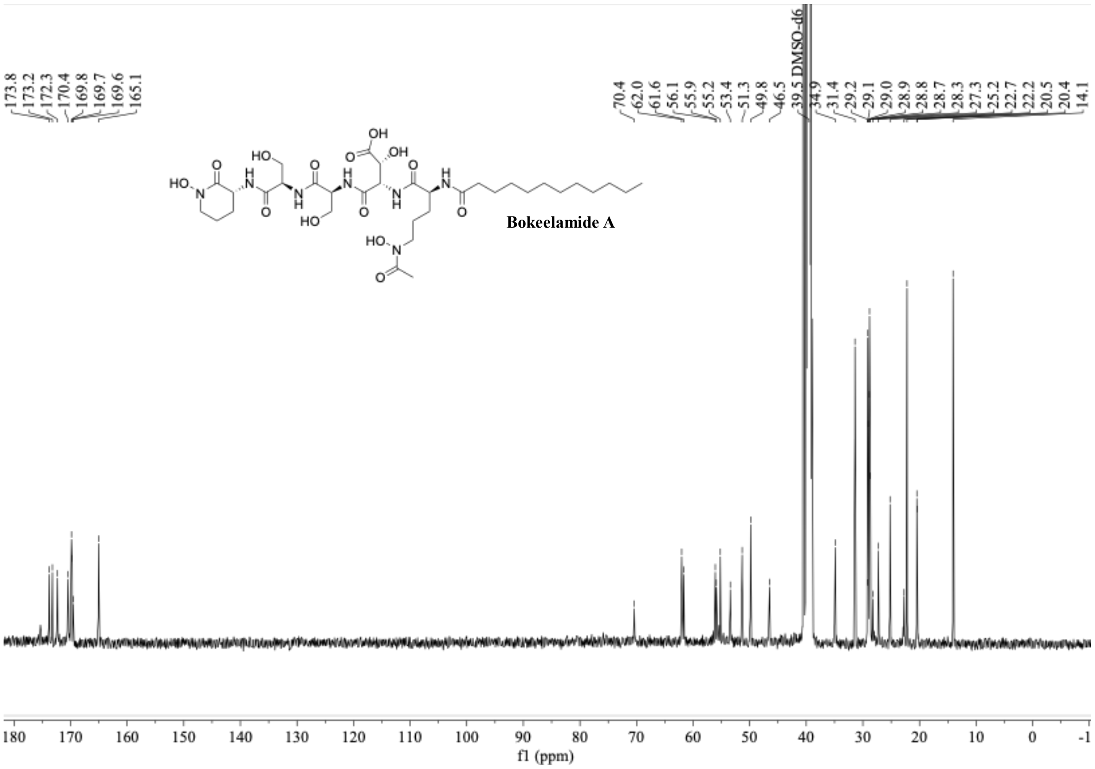

Figure S7: ^{13}C NMR spectrum of bokeelamide A (**1**) in d_6-DMSO (125 MHz)

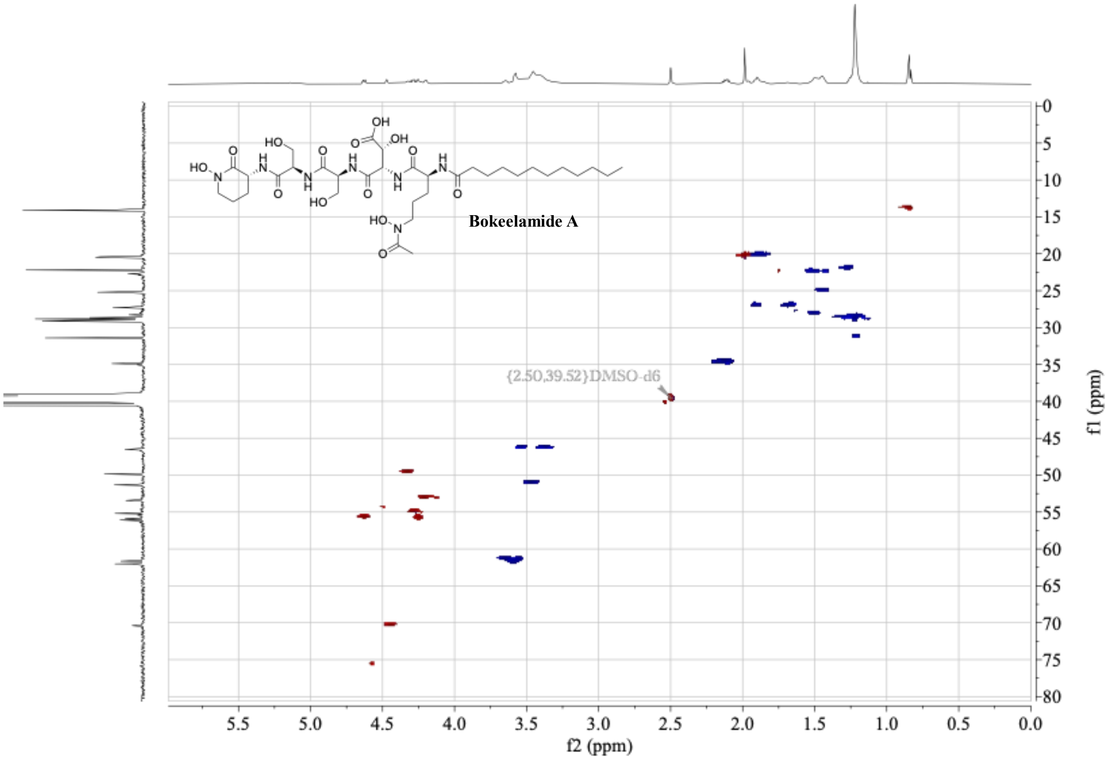

Figure S8: gHSQC spectrum of bokeelamide A (**1**) in d_6-DMSO (^{1}H 600 MHz)

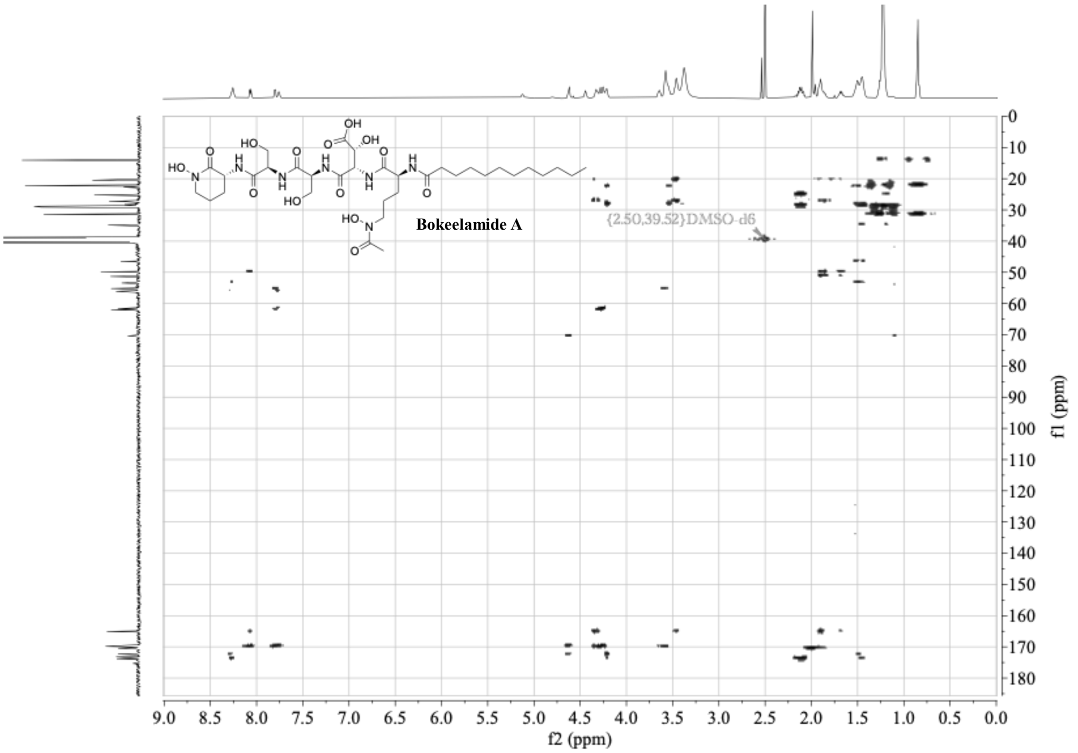

Figure S9: 5Hz HMBC spectrum of bokeelamide A (**1**) in d_6-DMSO (^{1}H 600 MHz)

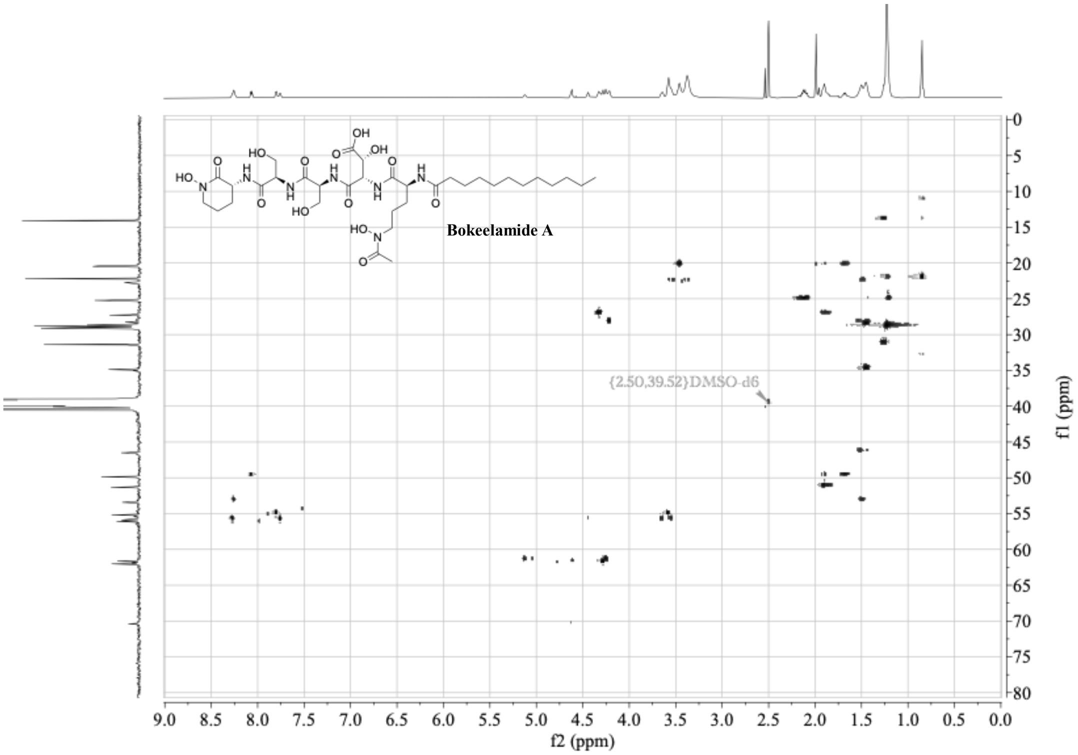

Figure S10: H2BC spectrum of bokeelamide A (**1**) in d_6-DMSO (^{1}H 600 MHz)

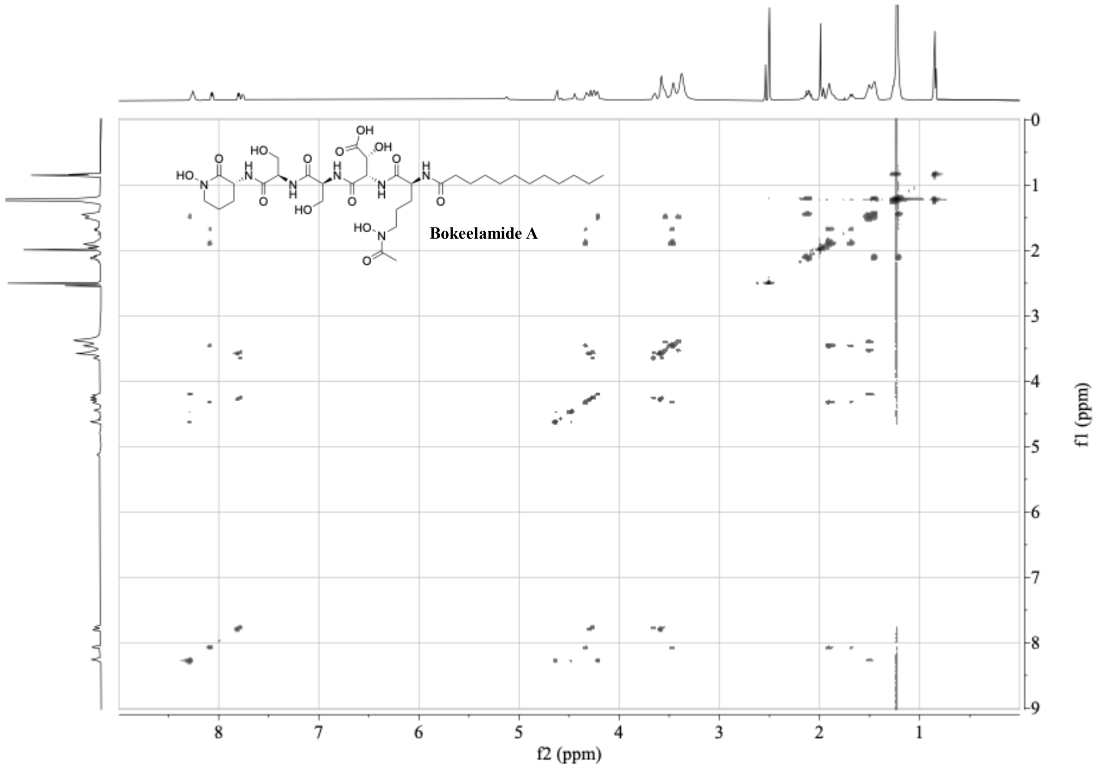

Figure S11: TOCSY spectrum of bokeelamide A (**1**) in *d*$_6$-DMSO (^{1}H 600 MHz)

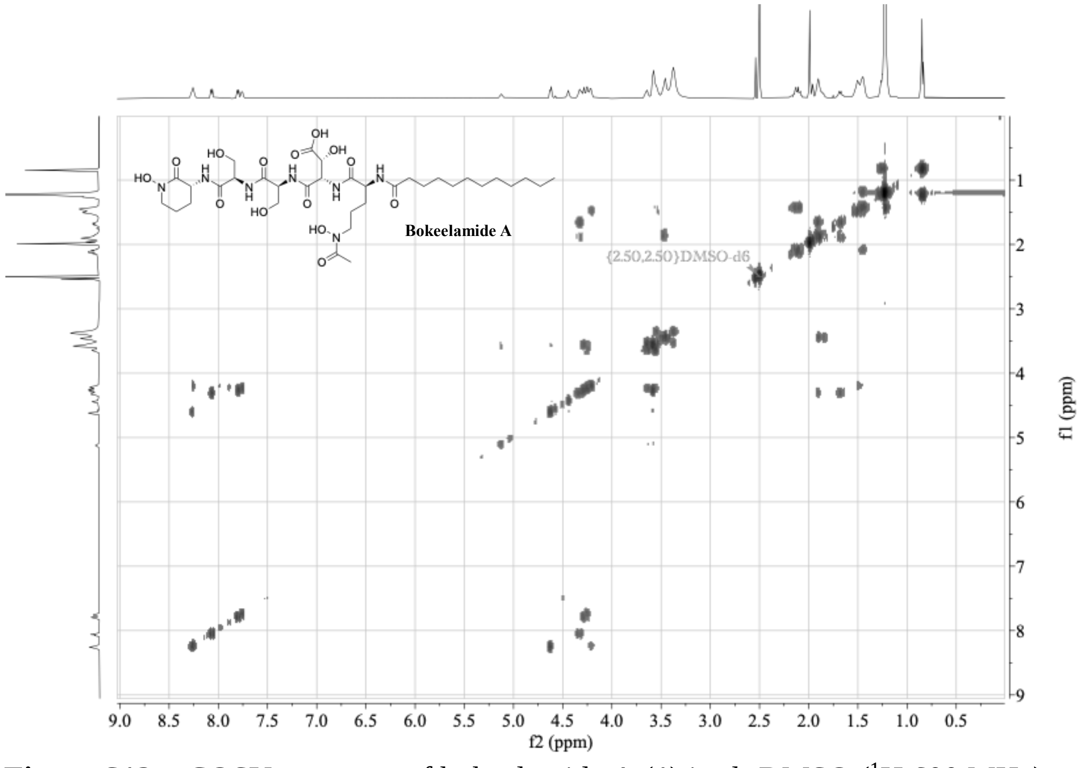

Figure S12: gCOSY spectrum of bokeelamide A (**1**) in *d*$_6$-DMSO (^{1}H 600 MHz)

Table S2. NMR assignments for bokeelamide A (**1**) and observed correlations in d_6-DMSO

**Residue**	**Position**	**δ_C, type[a]**	**δ_H (*J* in Hz)[b]**	**HMBC[b]**	**TOCSY[b]**	**H2BC[b]**
Cyclic *N*5-OH-D-Orn1	1	165.1, C				
	2	49.8, CH	4.32, m	1, 3, 4		3
	3a	27.3, CH_2	1.90, m	1, 2, 4, 5	2, 5, 2-NH	2, 4
	3b		1.67, q (10.6)	1, 2, 4, 5		2, 4
	4a	20.4, CH_2	1.89, m	1, 2, 5		3, 5
	4b		1.85, m	1, 2, 5		5
	5	51.3, CH_2	3.46, m	1, 3, 4	2, 3, 2-NH	4
	2-NH		8.07, d (8.4)	1, 2, 6		2
	5N-OH					
D-Ser1	6	169.8, C				
	7	55.2, CH	4.28, m	6, 8		8
	8	62.0, CH_2	3.57, m	6, 7	7, 7-NH	7
	7-NH		7.81, d (7.8)	7, 8, 9		7
	8-OH		5.14, brs		8	
L-Ser2	9	169.7, C				
	10	55.9, CH	4.25, m	9, 11		11
	11	61.7, CH_2	3.64, m		10, 10-NH	10
	10-NH		7.77, d (6.3)	10, 11, 12		10
	11-OH		5.14, brs		10, 11, 10-NH	11
L-*erythro*-βOH-Asp	12	169.6, C				
	13	55.9, CH	4.62, d (7.3)	12, 14, 15, 16		
	14	70.4, CH	4.47, brs		13-NH	13
	15	173.2, C				
	13-NH		8.28, m	13, 16		13
	14-OH					
	15-OH					
*N*5OH-*N*5-Ac-L-Orn2	16	172.3, C				
	17	53.4, CH	4.21, m	16, 18, 19	19, 20	18
	18	28.3, CH_2	1.49, m	16, 17, 19		17, 19
	19	22.7, CH_2	1.50, m	16, 17, 18, 19	20, 17, 17-NH	18, 20
	20a	46.5, CH_2	3.54, m	18, 19	17, 19, 17-NH	19
	20b		3.41, brs			19
	21	170.4, C				
	22	20.5, CH_3	1.99, s	21		
	17-NH		8.27, m	17, 23		17
	20-N-OH		5.31, brs		22	
C12:0	23	173.8, C				
	24	34.9, CH_2	2.11, tt (7.4, 6.7)	23, 25, 26-31	25, 26-31	25
	25	25.2, CH_2	1.44, m	23, 24, 26-31	26-31, 24	24, 26-31
	26-31[c]	28.7-29.2, CH_2	1.22, m	24, 25, 33	24, 25, 34	25
	32	31.4, CH_2	1.22, m			26-31, 33
	33	22.2, CH_2	1.25, m	26-31, 32, 34		32, 34
	34	14.1, CH_3	0.84, t (7.1)	32, 33		33

[a]125 MHz for ^{13}C NMR; [b]600 MHz for ^{1}H NMR, HMBC, TOCSY, and H2BC; [c]CH_2 envelope

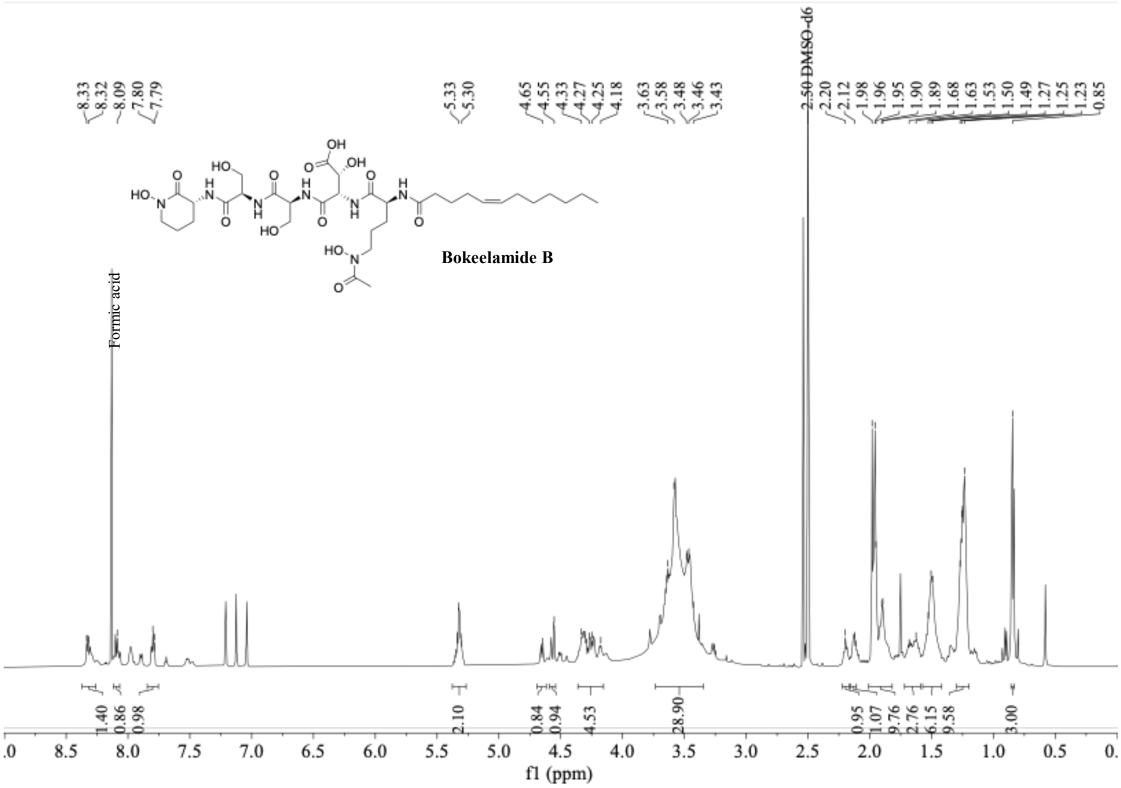

Figure S13: ^{1}H NMR spectrum of bokeelamide B (**2**) in d_6-DMSO with 0.1% TFA (600 MHz)

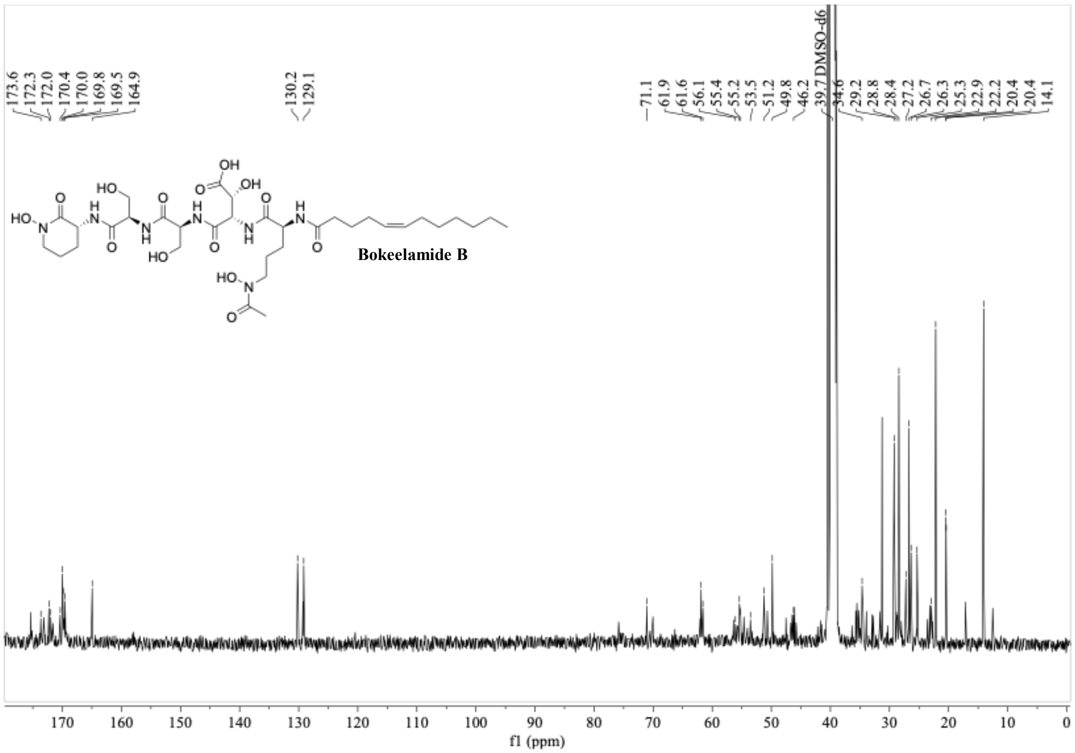

Figure S14: ^{13}C NMR spectrum of bokeelamide B (**2**) in d_6-DMSO with 0.1% TFA (150 MHz)

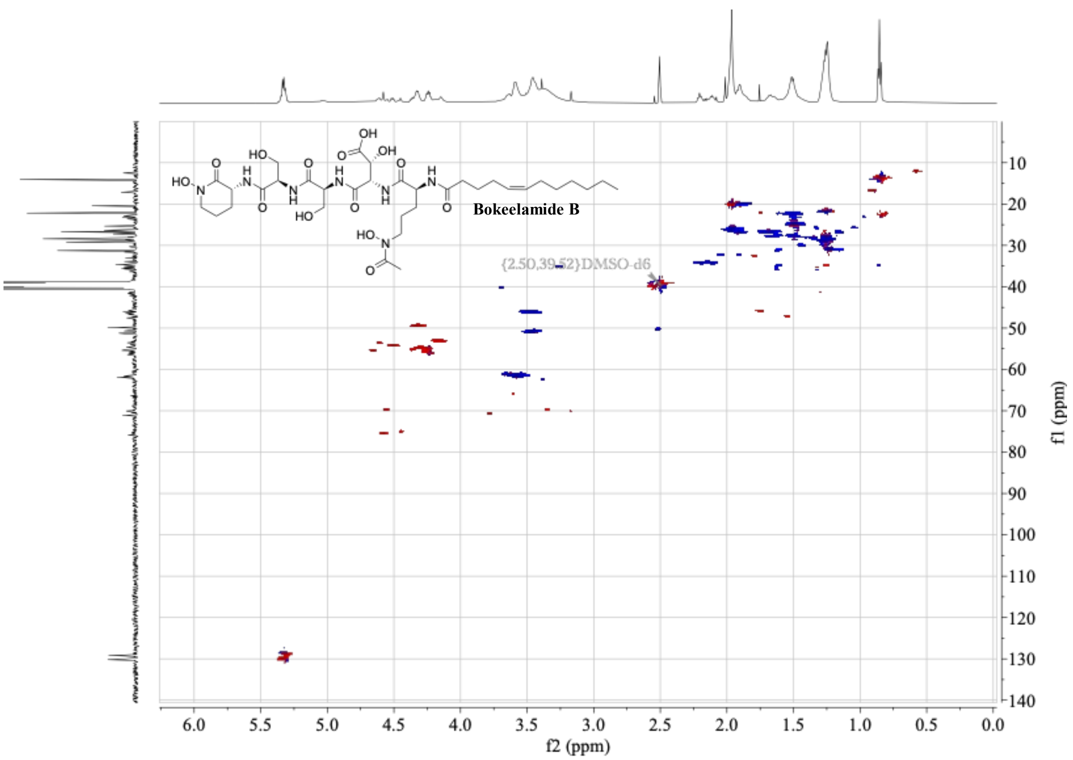

Figure S15: gHSQC spectrum of bokeelamide B (**2**) in d_6-DMSO with 0.1% TFA (^{1}H 600 MHz)

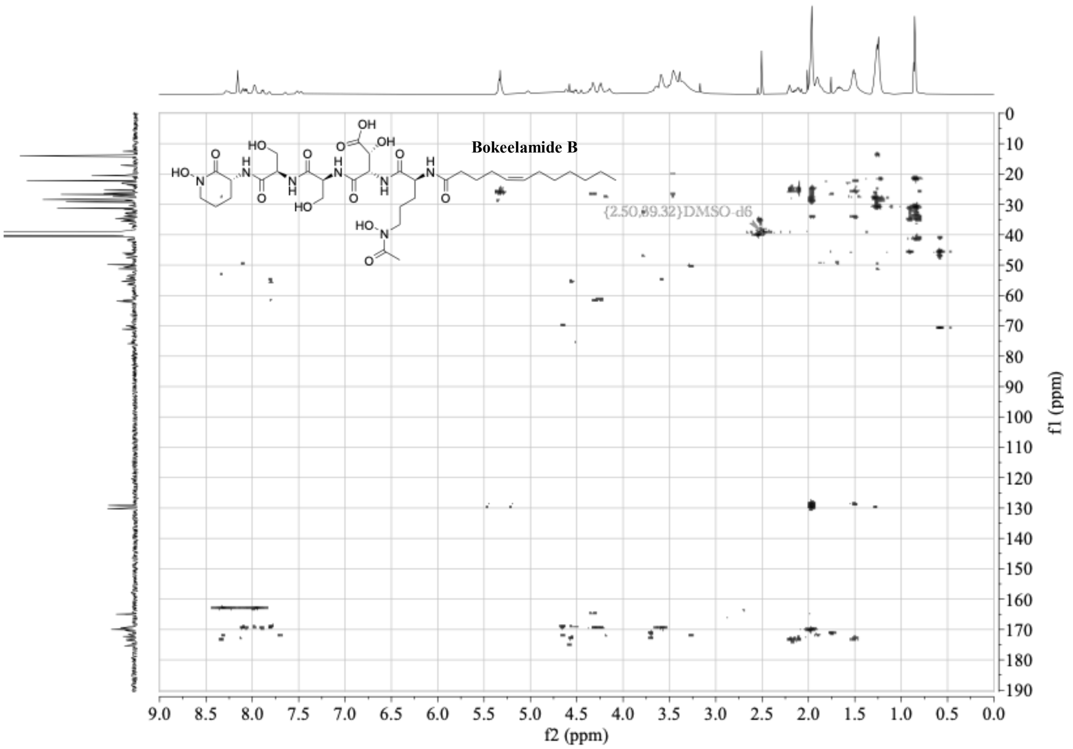

Figure S16: HMBC spectrum of bokeelamide B (**2**) in d_6-DMSO with 0.1% TFA (^{1}H 600 MHz)

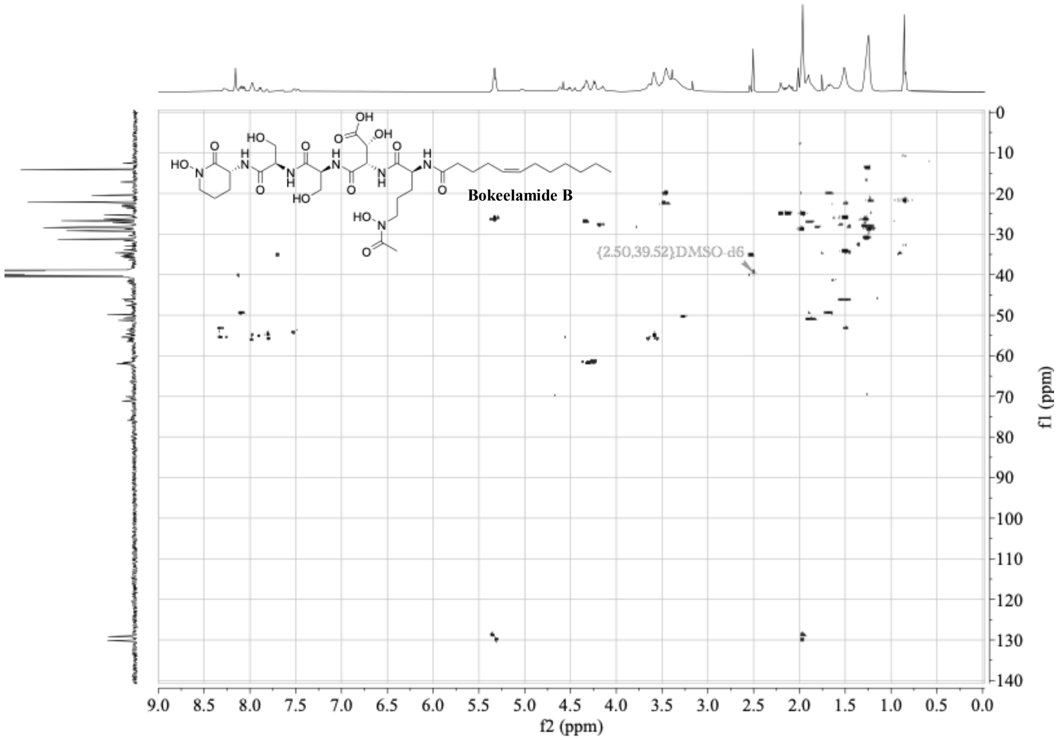

Figure S17: H2BC spectrum of bokeelamide B (**2**) in d_6-DMSO with 0.1% TFA (^{1}H 600 MHz)

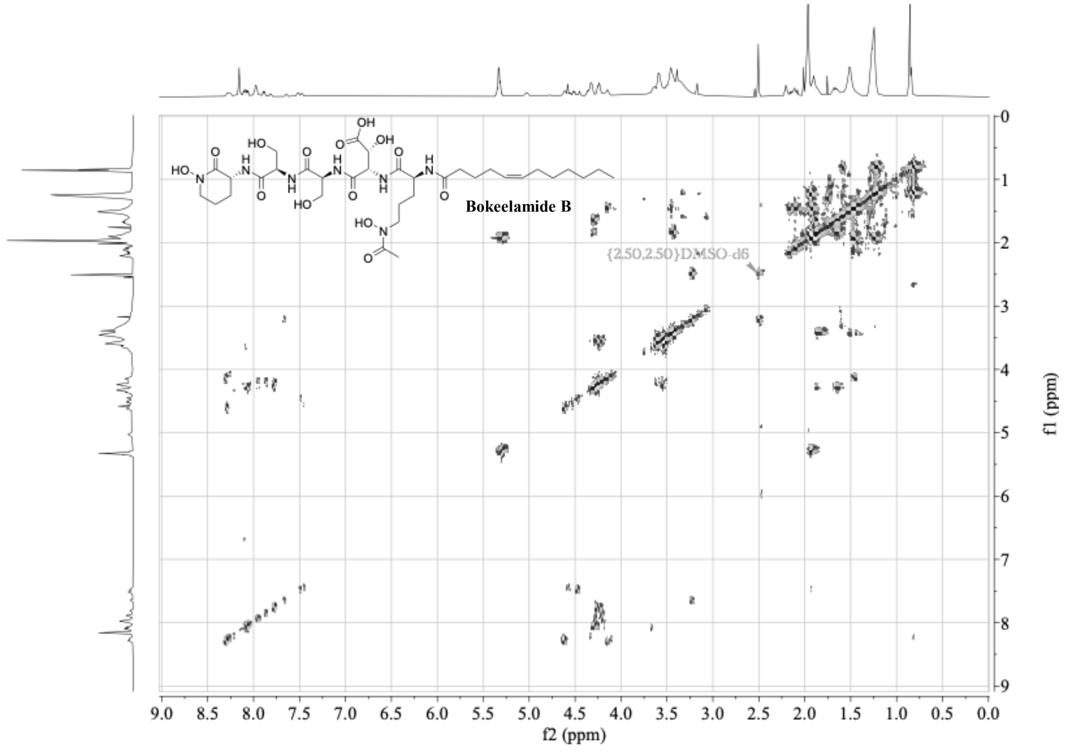

Figure S18: TOCSY spectrum of bokeelamide B (**2**) in d_6-DMSO with 0.1% TFA (^{1}H 600 MHz)

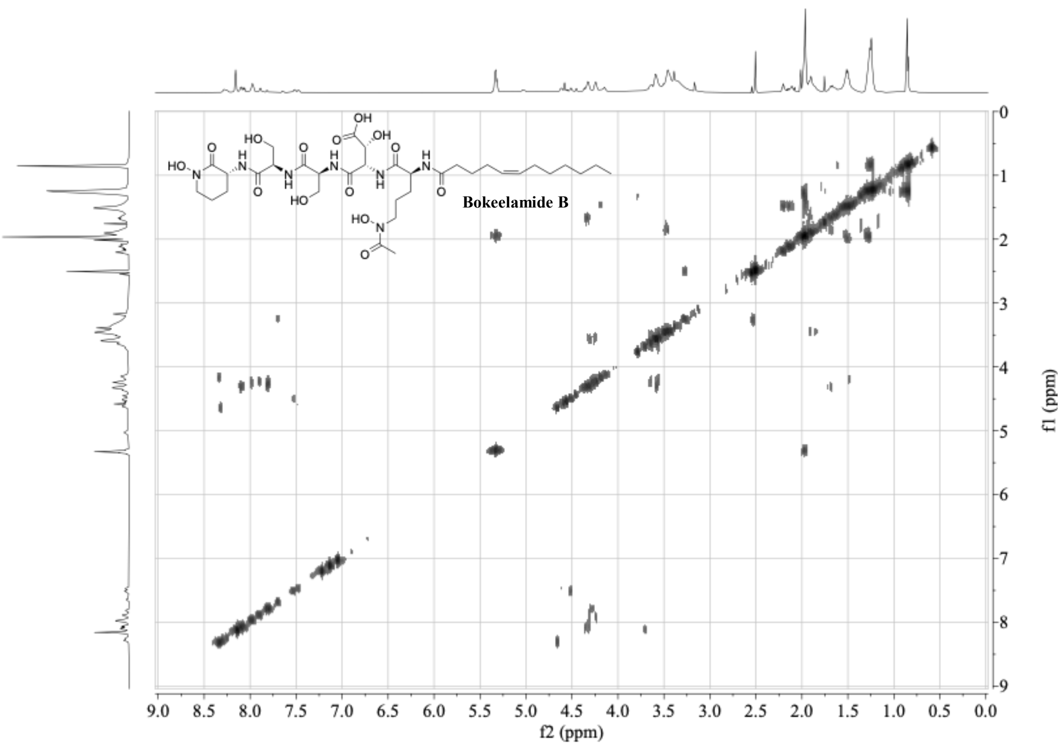

Figure S19: gCOSY spectrum of bokeelamide B (**2**) in d_6-DMSO with 0.1% TFA (^{1}H 600 MHz)

Table S3: NMR assignments for bokeelamide B (**2**) and observed correlations in d_6-DMSO

Residue	Position	δ_C, type[a]	δ_H (*J* in Hz)[b]	HMBC[b]	TOCSY[b]	H2BC[b]
Cyclic *N*^{5}OH-	1	164.9, C				
D-Orn$_1$	2	49.8, CH	4.33, m	1, 3	2-NH, 3	3
	3a	27.2, CH_2	1.68, m	2	2, 4	2, 4
	3b		1.90, m	2	2	2
	4	20.4, CH_2	1.89, m		3, 5	3, 5
	5	51.2, CH_2	3.46, m	3, 4	4	4
	2-NH		8.09, d (8.4)	2, 6	2	2
	5N-OH					
D-Ser$_1$	6	170.0, C				
	7	55.2, CH	4.27, m	6, 8	7-NH, 8	8
	8	61.9, CH_2	3.58, d (5.6)	6, 7	7	7
	7-NH		7.80, d (8.6)	7, 8, 9	7	7
	8-OH					
L-Ser$_2$	9	169.8, C				
	10	56.1, CH	4.25, m	9, 11	10-NH	11
	11	61.6, CH_2	3.63, m	9	10	10
	10-NH		7.79, d (8.3)	10, 12	10	10
	11-OH					
L-*erythro*-	12	169.5, C				
βOH-Asp	13	55.4, CH	4.65, dd (9.1, 2.3)	12, 14, 16	13-NH	14
	14	71.1, CH	4.55, d (2.3)	12, 13, 15		13
	15	172.3, C				
	13-NH		8.32, m	16	13	13
	14-OH					
	15-OH					
N^{5}OH-*N*^{5}-	16	172.0, C				
Ac-L-Orn$_2$	17	53.5, CH	4.18, m	16, 18	18	18
	18a	28.4, CH_2	1.49, m	19	17, 20	17, 19
	18b		1.63, m			19
	19	22.9, CH_2	1.53, m	18, 20	20	18, 20
	20a	46.2, CH_2	3.43, m			19
	20b		3.48, m		18	19
	21	170.4, C				
	22	20.4, CH_3	1.98, m	21		21
	17-NH		8.33, m	17, 18, 23	17	17
	20-N-OH					
C12:1Δ^5	23	173.6, C				
	24a	34.6, CH_2	2.12, m	23, 25, 26	25	25
	24b		2.20, t (7.0)	23, 25, 26	25	25
	25	25.3, CH_2	1.50, m	23, 24, 26, 27	26	24, 26
	26	26.3, CH_2	1.95, m	24, 25, 27, 28	25, 27	25, 27
	27	129.1, CH	5.30, m	25, 29	26, 29	26, 28
	28	130.2, CH	5.33, m	26, 30	26, 29	27, 29
	29	26.7, CH_2	1.96, m	27, 28, 30, 31-32	30, 31-32	28, 30
	30	28.8, CH_2	1.27, m	29, 31-32	29	29
	31-32[c]	29.2, CH_2	1.23, m	33	29, 30	30, 33
	33	22.2, CH_2	1.25, m	31-32, 34	34	34, 31-32
	34	14.1, CH_3	0.85, t (6.3)	31-32, 33	30, 31-32, 33	33

[a]150 MHz for ^{13}C NMR; [b]600 MHz for ^{1}H NMR, HMBC, TOCSY, and H2BC; [c]CH_2 envelope

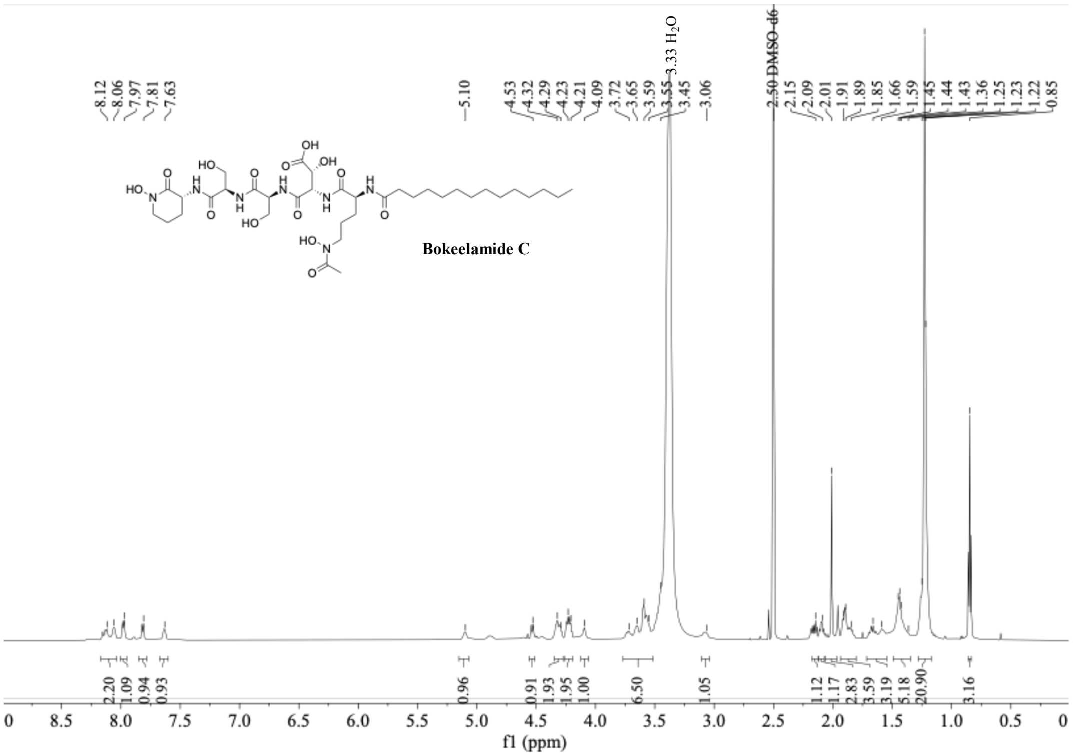

Figure S20: ^{1}H NMR spectrum of bokeelamide C (**3**) in d_6-DMSO (600 MHz)

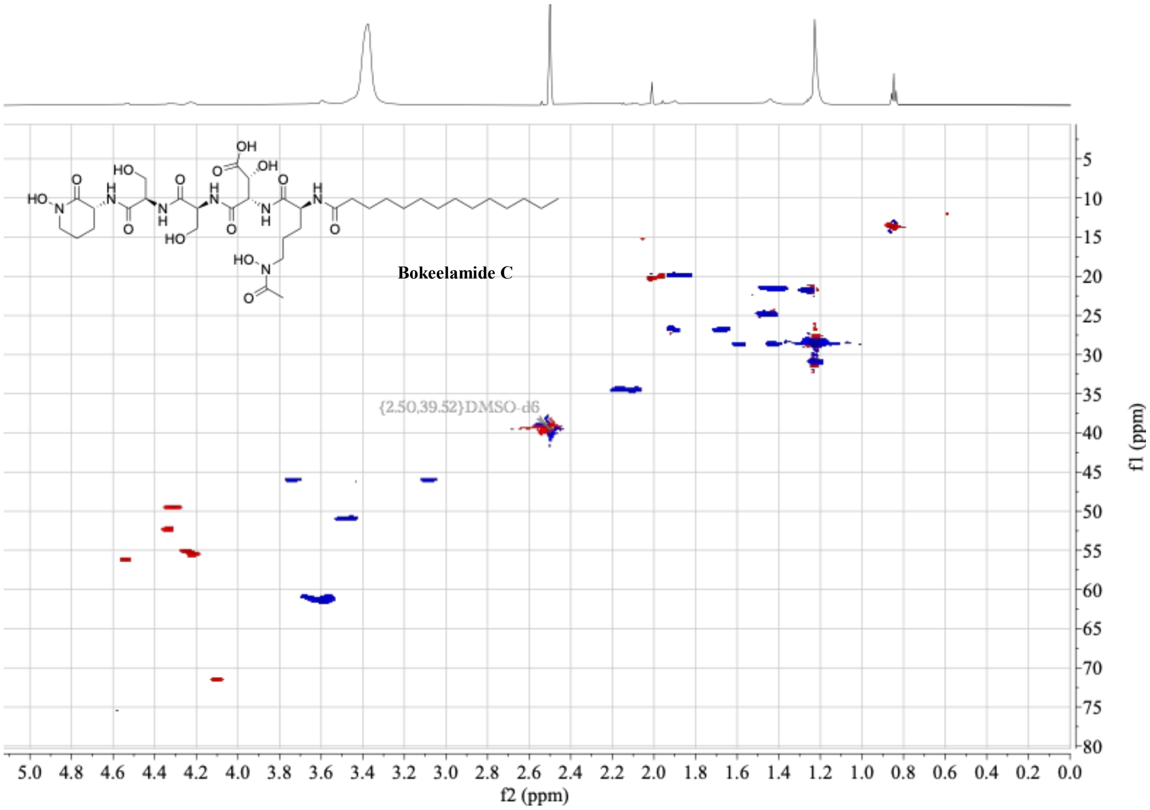

Figure S21: gHSQC spectrum of bokeelamide C (**3**) in d_6-DMSO (^{1}H 600 MHz)

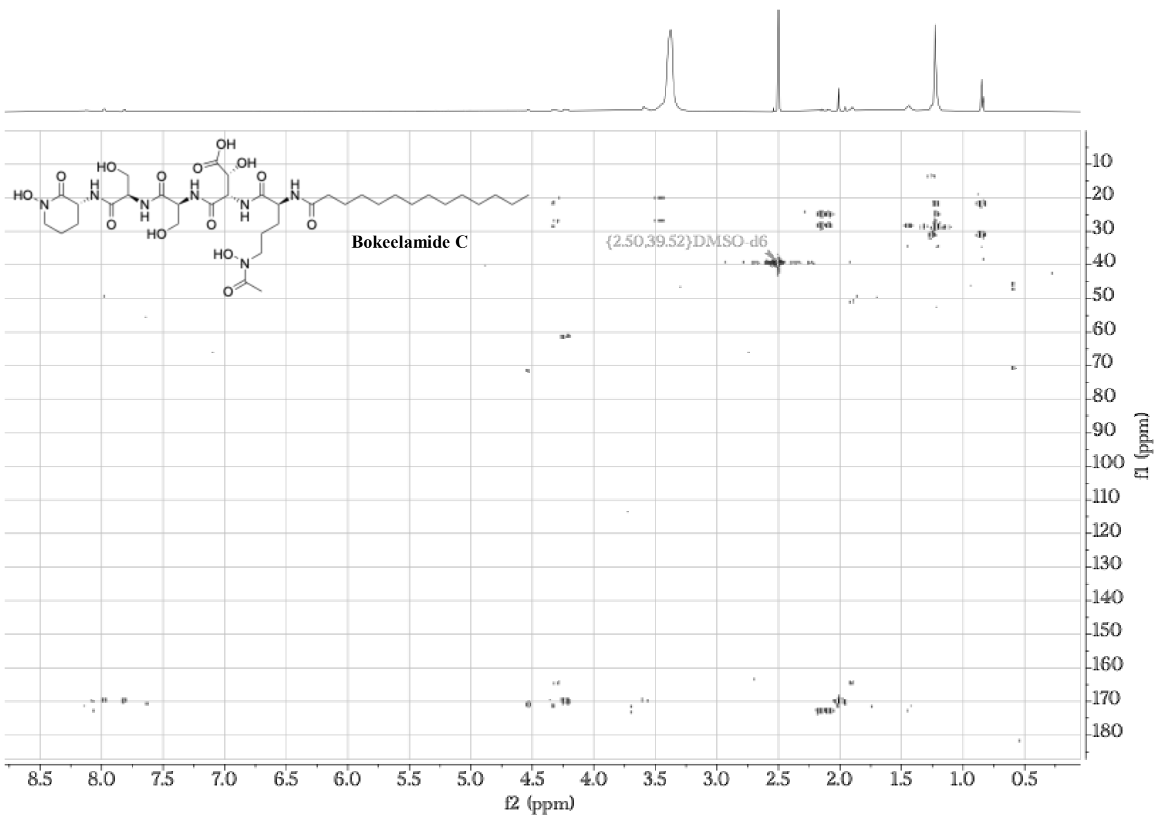

Figure S22: HMBC spectrum of bokeelamide C (**3**) in *d*$_6$-DMSO (^{1}H 600 MHz)

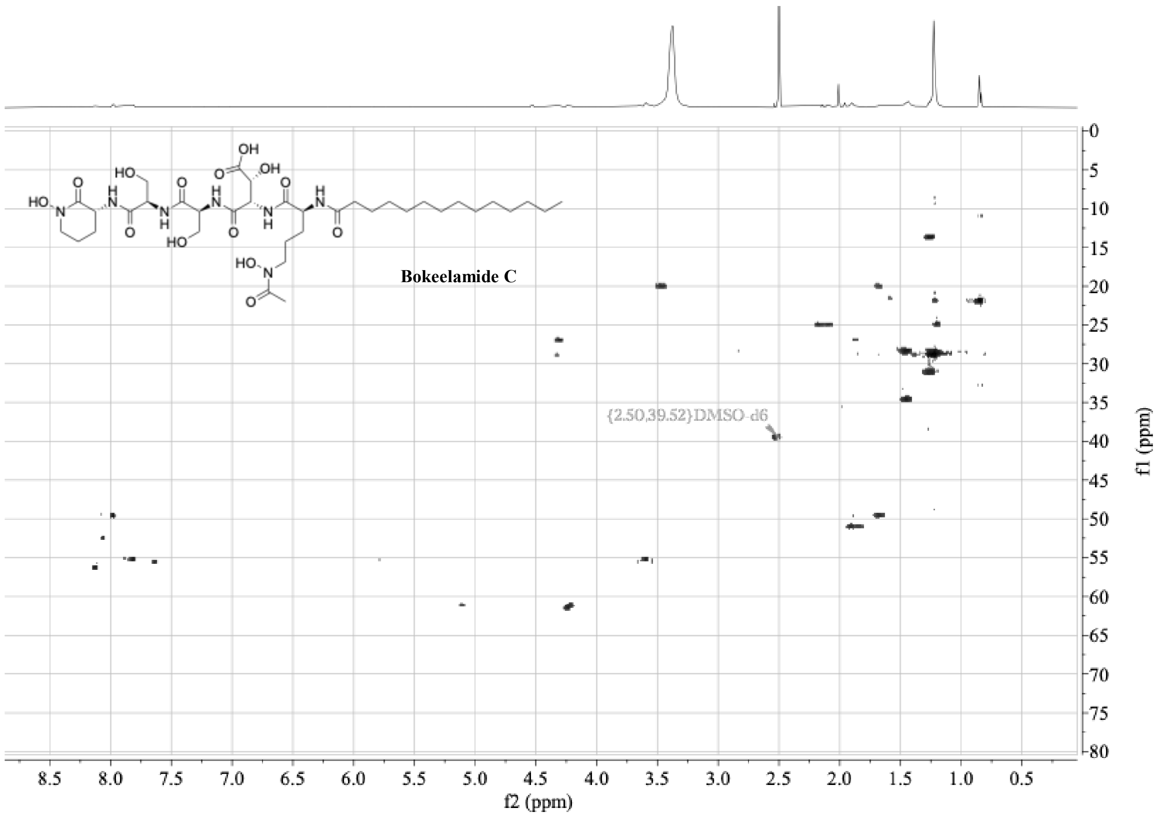

Figure S23: H2BC spectrum of bokeelamide C (**3**) in *d*$_6$-DMSO (^{1}H 600 MHz)

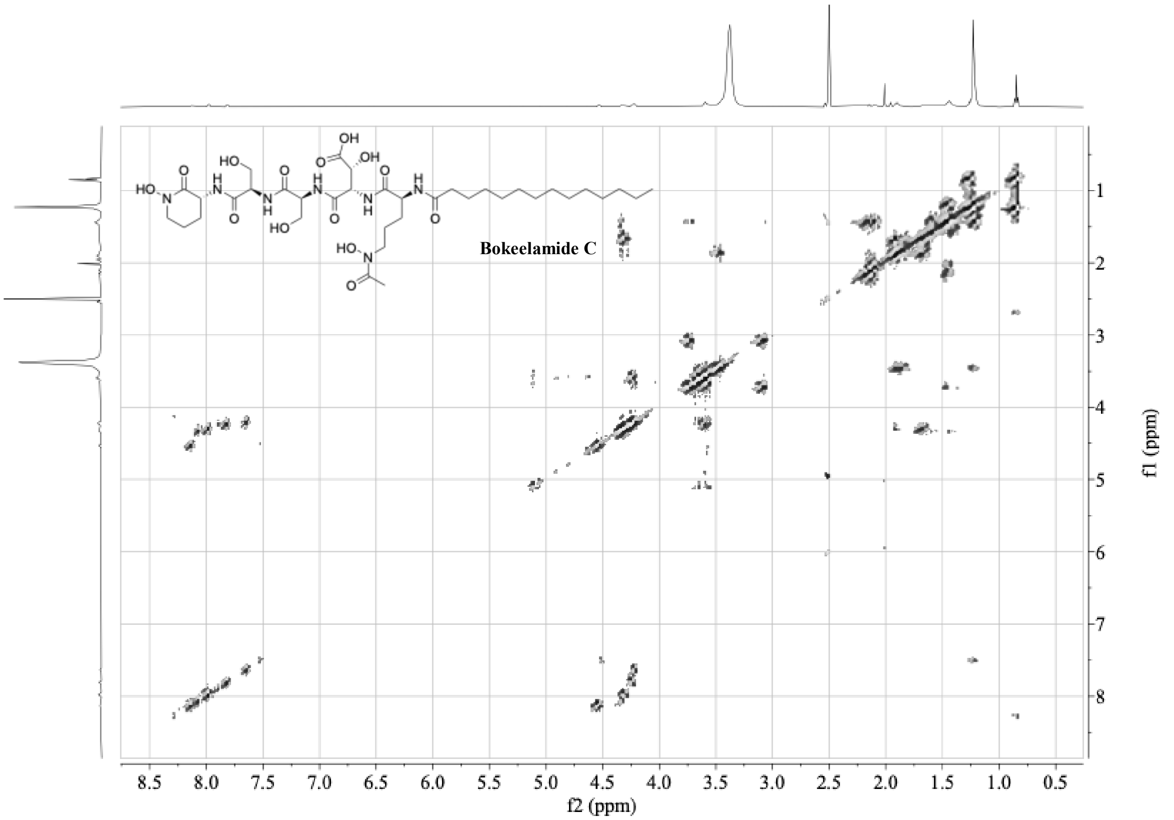

Figure S24: TOCSY spectrum of bokeelamide C (**3**) in *d*6-DMSO (^{1}H 600 MHz)

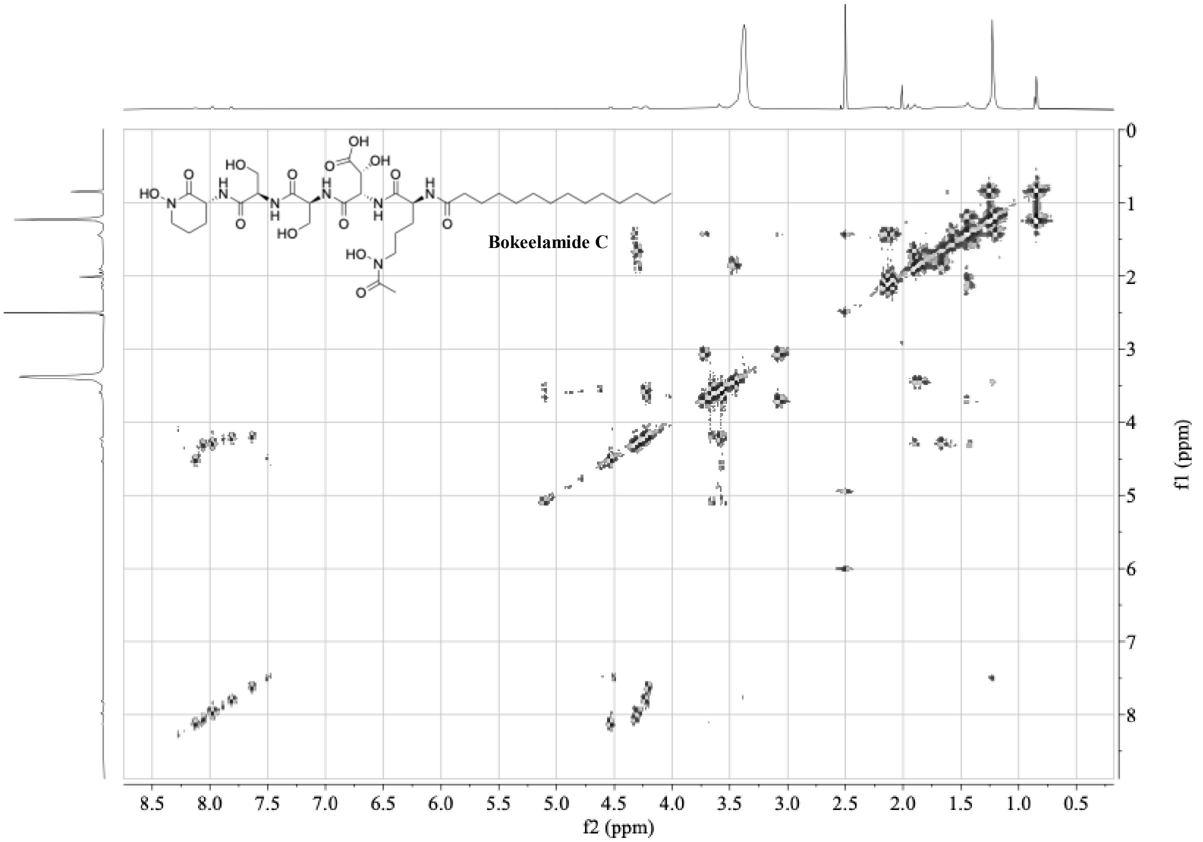

Figure S25: dqfCOSY spectrum of bokeelamide C (**3**) in *d*6-DMSO (^{1}H 600 MHz)

Table S4: NMR assignments for bokeelamide C (**3**) and observed correlations in d_6-DMSO

Residue	Position	δC, type[a]	δH (*J* in Hz)[b]	HMBC[b]	TOCSY[b]	H2BC[b]
Cyclic N^5OH-	1	164.9, C				
D-Orn1	2	49.5, CH	4.29, m	1, 3	2-NH, 3	3
	3a	27.0, CH_2	1.66, dq (1.9, 10.9)	2	2, 3b	2, 4
	3b		1.91, m	2	2, 3a	2
	4a	20.0, CH_2	1.85, m		5	3, 5
	4b		1.89, m	1, 5	5	3, 5
	5	51.0, CH_2	3.45, m	3, 4	4	4
	2-NH		7.97, d (8.0)	6	2	2
	5N-OH					
D-Ser1	6	169.7, C				
	7	55.2, CH	4.23, m	6, 8	7-NH, 8	8
	8	61.4, CH_2	3.59, m		7	7
	7-NH		7.81, d (7.8)	9	7	7
	8-OH					
L-Ser2	9	169.8, C				
	10	55.5, CH	4.21, m	9, 11	10-NH	11
	11a	61.3, CH_2	3.55, m		10, 11-OH	10
	11b		3.65, m		10, 11-OH	10
	10-NH		7.63, d (6.7)	12	10	10
	11-OH		5.10, brs		11	11
L-*erythro*-	12	170.9, C				
βOH-Asp	13	56.3, CH	4.53, dd (1.8, 8.8)	12, 14	13-NH	
	14	71.5, CH	4.09, s			
	15					
	13-NH		8.12, d (8.3)	16	13	13
	14-OH					
	15-OH					
N^5OH-N^5-	16	171.5, C				
Ac-L-Orn2	17	52.4, CH	4.32, m	16, 18, 19	17-NH	18
	18a	28.7, CH_2	1.43, m		17, 18b	
	18b		1.59, m		17, 18a	19
	19a	21.7, CH_2	1.36, m		20	
	19b		1.46, m		20	18, 20
	20a	46.1, CH_2	3.06, m		20b	
	20b		3.72, m		19, 20a	
	21	170.0, C				
	22	20.4, CH_3	2.01, s	21		
	17-NH		8.06, m	23	17	17
	20-N-OH					
C14:0	23	172.9, C				
	24a	34.6, CH_2	2.09, t (7.2)	23, 25, 26-33	24b, 25	25
	24b		2.15, t (7.4)	23, 25, 26-33	24a, 25	25
	25	25.0, CH_2	1.44, m	23, 24, 26-33	24, 26-33	24
	26-33[c]	28.8, CH_2	1.23, m	25, 34	25	25, 34
	34	31.1, CH_2	1.22, m	26-33, 35	35	26-33, 35
	35	21.9, CH_2	1.26, m	34, 36	36	34, 36
	36	13.7, CH_3	0.85, t (6.9)	34, 35	35	35

[a]Carbon shifts and multiplicity interpreted from HSQC and HMBC spectra; [b]600 MHz for ^{1}H NMR, HMBC, TOCSY, and H2BC; [c]CH_2 envelope

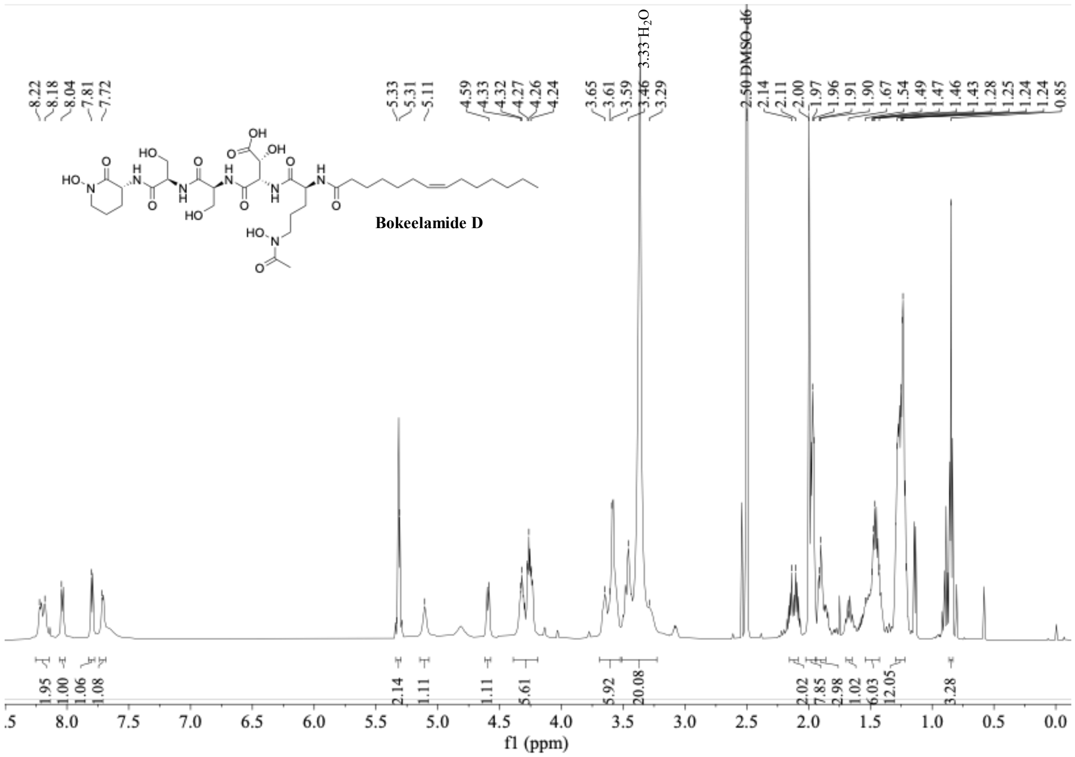

Figure S26: ^{1}H NMR spectrum of bokeelamide D (**4**) in d_6-DMSO (600 MHz)

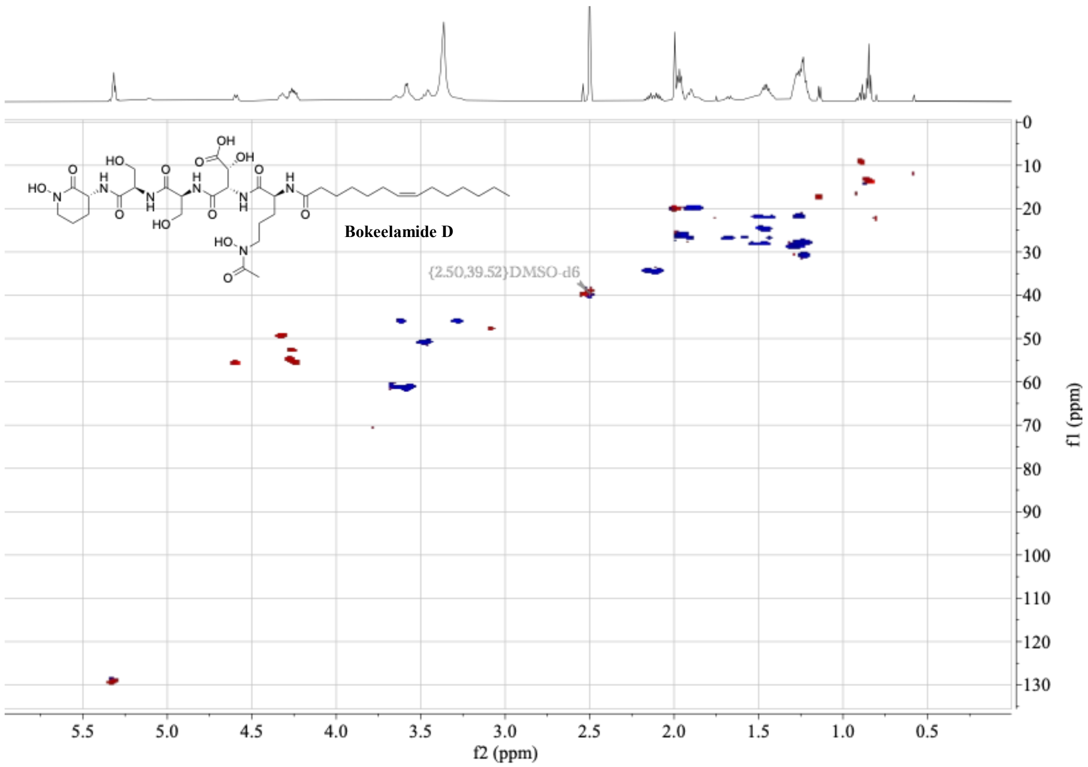

Figure S27: gHSQC spectrum of bokeelamide D (**4**) in d_6-DMSO (^{1}H 600 MHz)

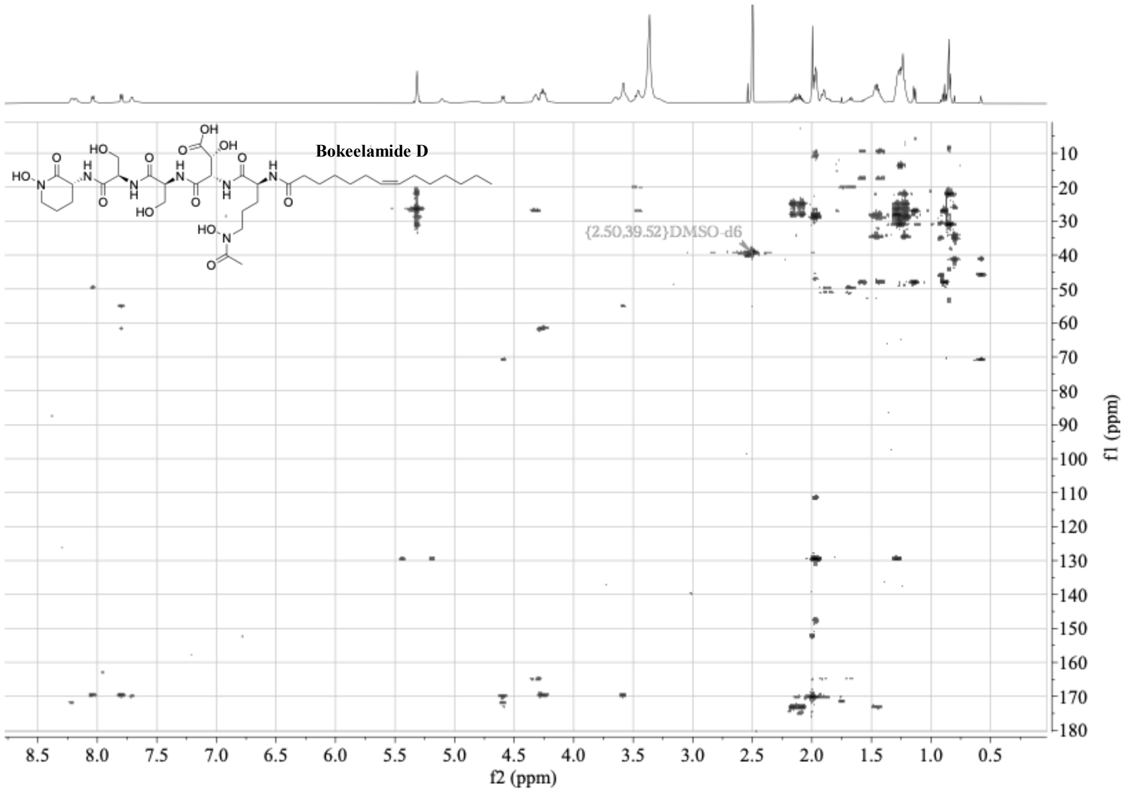

Figure S28: HMBC spectrum of bokeelamide D (**4**) in d_6-DMSO (^{1}H 600 MHz)

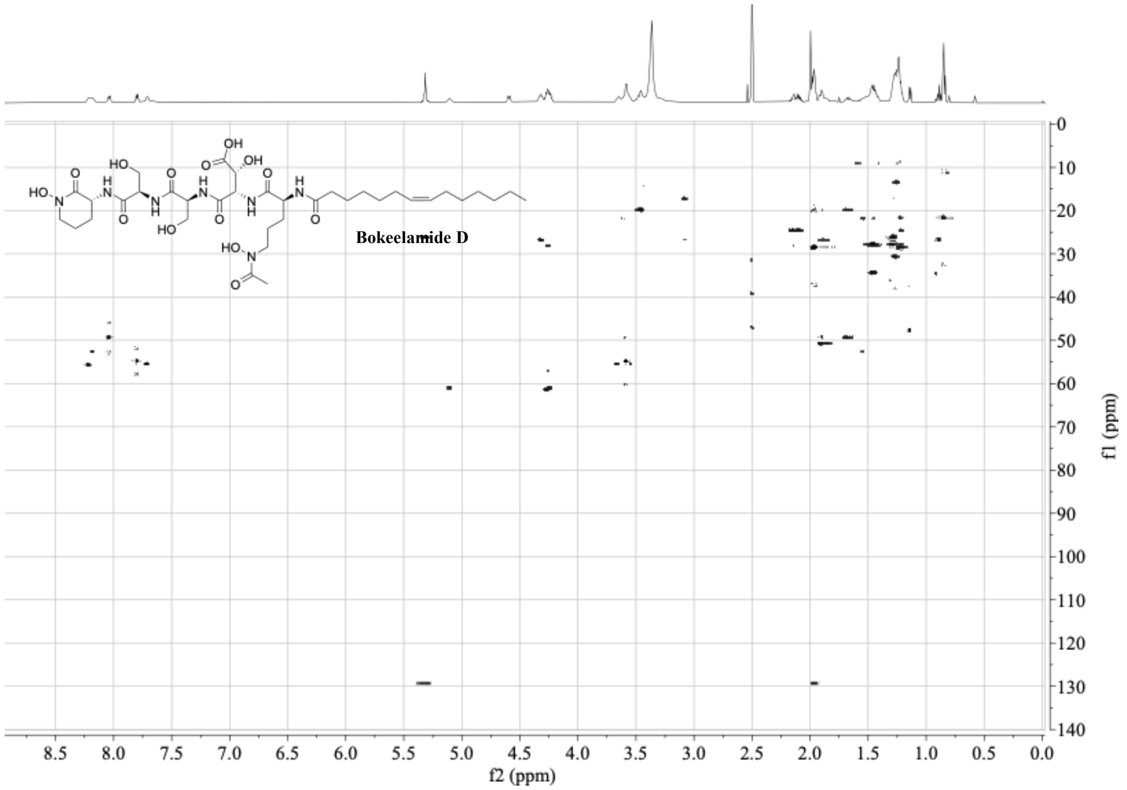

Figure S29: H2BC spectrum of bokeelamide D (**4**) in d_6-DMSO (^{1}H 600 MHz)

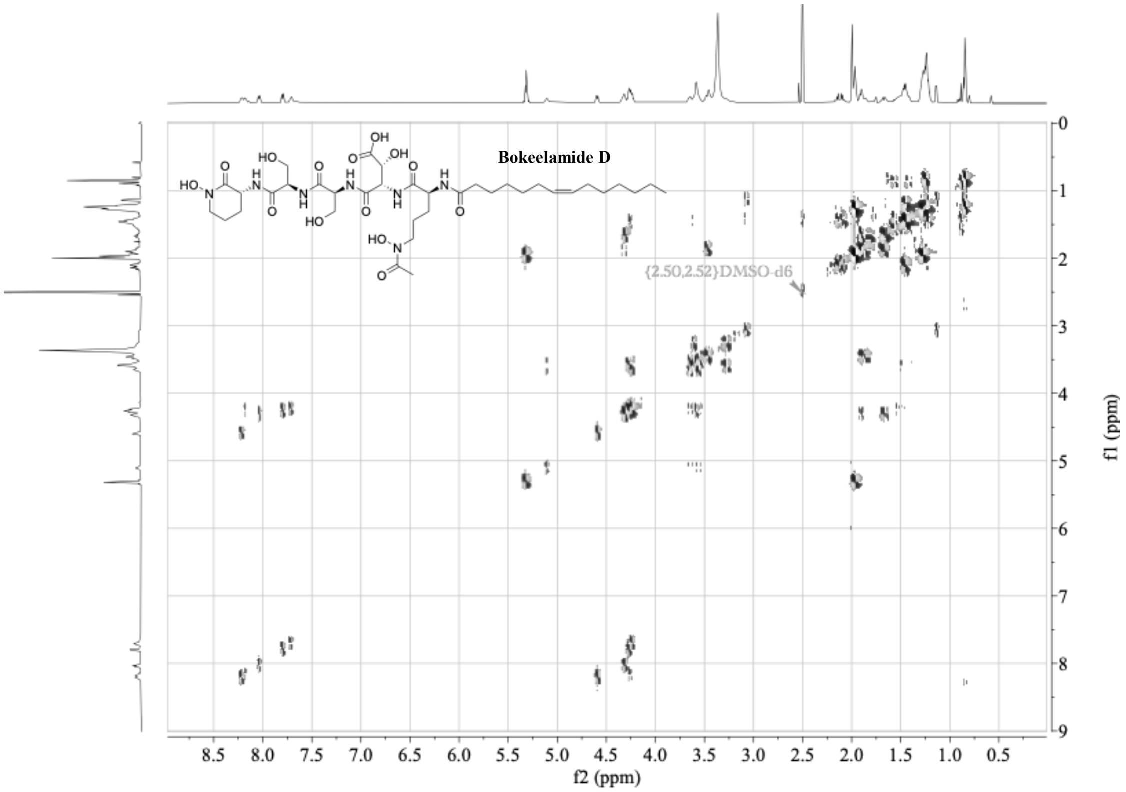

Figure S30: TOCSY spectrum of bokeelamide D (**4**) in d_6-DMSO (^{1}H 600 MHz)

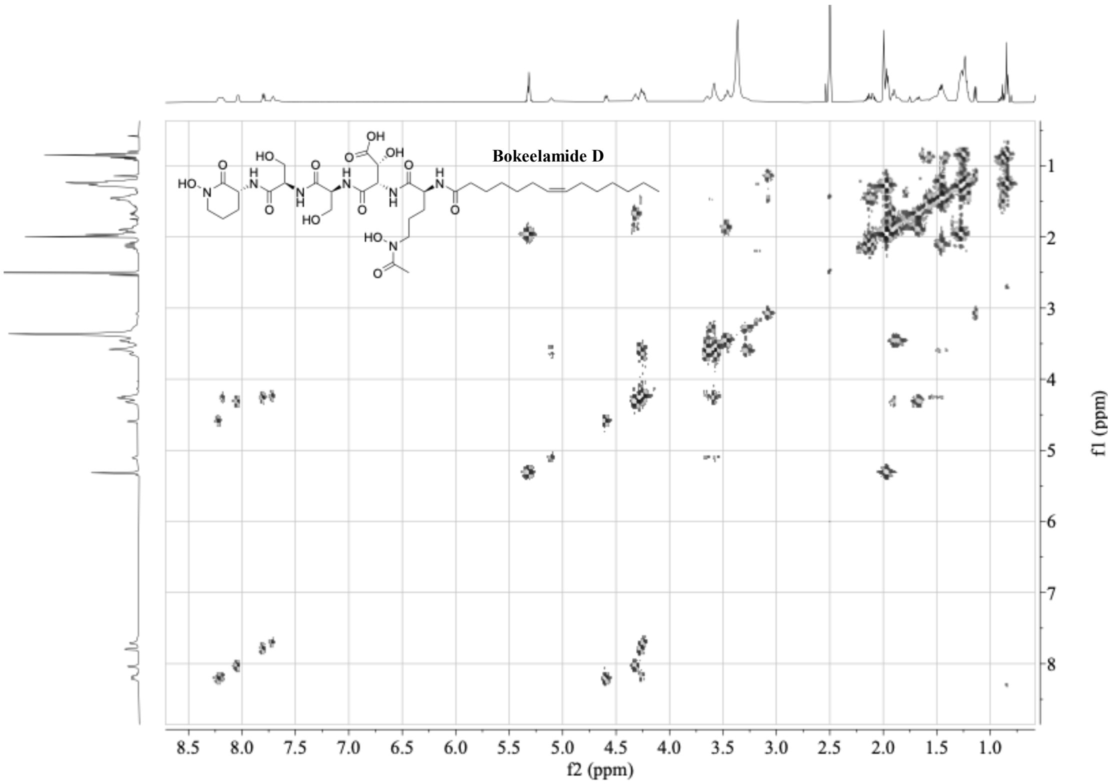

Figure S31: dqfCOSY spectrum of bokeelamide D (**4**) in d_6-DMSO (^{1}H 600 MHz)

Table S5: NMR assignments for bokeelamide D (**4**) and observed correlations in d_6-DMSO

Residue	Position	δ_C, type[a]	δ_H (*J* in Hz)[b]	HMBC[b]	TOCSY[b]	H2BC[b]
Cyclic N^5OH-	1	165.0, C				
D-Orn$_1$	2	49.5, CH	4.32, m	1, 3	2-NH, 3, 4	3
	3a	27.0, CH_2	1.67, m	1, 2, 4	2, 3b	2, 4
	3b		1.91, m	1, 2	2, 3a	2, 4
	4	20.0, CH_2	1.90, m	2, 5	5	3, 5
	5	50.9, CH_2	3.46, m	3, 4	4	4
	2-NH		8.04, d (8.2)	2, 6	2	2
	5N-OH					
D-Ser$_1$	6	169.8, C				
	7	55.0, CH	4.27, m	6, 8	7-NH, 8	8
	8	61.6, CH_2	3.59, d (5.6)	6, 7	77	7
	7-NH		7.81, d (8.0)	7, 8, 9	7	7
	8-OH					
L-Ser$_2$	9	169.7, C				
	10	55.6, CH	4.24, m	9, 11	10-NH, 11	11
	11	61.3, CH_2	3.65, m		10, 11-OH	10
	10-NH		7.72, d (6.5)	12	10	10
	11-OH		5.11, brs		11	11
L-*erythro*-	12	170.1, C				
βOH-Asp	13	55.8, CH	4.59, dd (1.1, 8.8)	12, 14, 15	13-NH	
	14	70.9, CH	4.33, m			
	15	172.0, C				
	13-NH		8.22, d (8.3)	16	13	13
	14-OH					
	15-OH					
N^5OH-N^5-	16	171.9, C				
Ac-L-Orn$_2$	17	52.8, CH	4.26, m		17-NH, 18	18
	18a	28.3, CH_2	1.47, m	17, 19	17	17, 19
	18b		1.54, m	17	17	17, 19
	19a	22.1, CH_2	1.43, m	18	20	18
	19b		1.49, m	18	20	18, 20
	20a	46.1, CH_2	3.29, m		19, 20b	19
	20b		3.61, m		19, 20a	19
	21	170.3, C				
	22	20.2, CH_3	2.00, s	21		
	17-NH		8.18, s		17	17
	20-N-OH					
C14:1Δ^7	23	173.3, C				
	24a	34.6, CH_2	2.11, q (7.3)	23, 25, 26	24b, 25	25
	24b		2.14, q (7.3)	23, 25, 26	24a, 25	25
	25	24.8, CH_2	1.46, m	23, 24,	24	24, 26
	26	28.0, CH_2	1.24, m	24		25, 27
	27	28.7, CH_2	1.28, m	29, 30	28	26, 28
	28	26.4, CH_2	1.97, m	26, 27, 29, 30, 31	29, 30	27, 29
	29	129.5, CH	5.33, m	28, 30, 31	28, 30, 31	28, 30
	30	129.3, CH	5.31, m	28, 29, 31	28, 29, 31	29, 31
	31	26.3, CH_2	1.96, m	28, 29, 30	29, 30, 32-34	30
	32-34[c]	28.0-30.9, CH_2	1.24, m	31, 35	31, 35	31, 35
	35	21.9, CH_2	1.25, m	36	36	32-34, 36
	36	13.8, CH_2	0.85, t (6.8)	32-34, 35	35	35

[a]Carbon shifts and multiplicity interpreted from HSQC and HMBC spectra; [b]600 MHz for ^{1}H NMR, HMBC, TOCSY, and H2BC; [c]CH_2 envelope

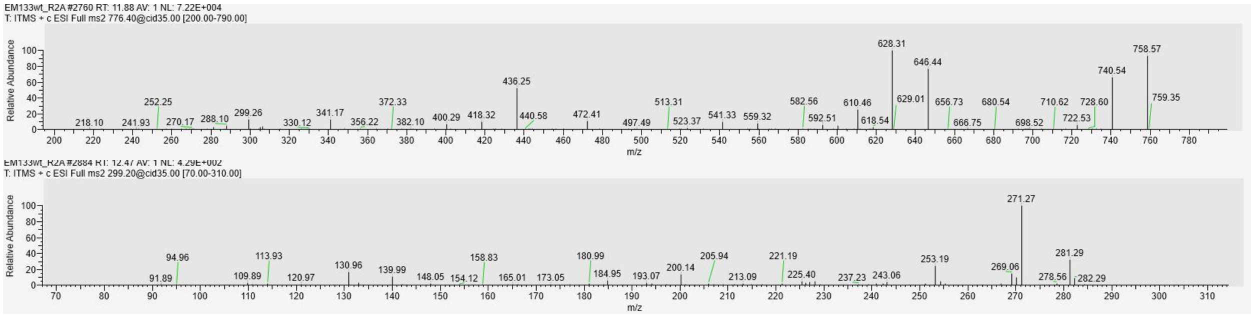

Figure S32: Fragmentation of bokeelamide G (**7**); MS^2 of the parent 776.4 *m/z* (top) and pseudo-MS^3 of the precursor de-acetylated b_1 fragment, 299.2 *m/z* (bottom).

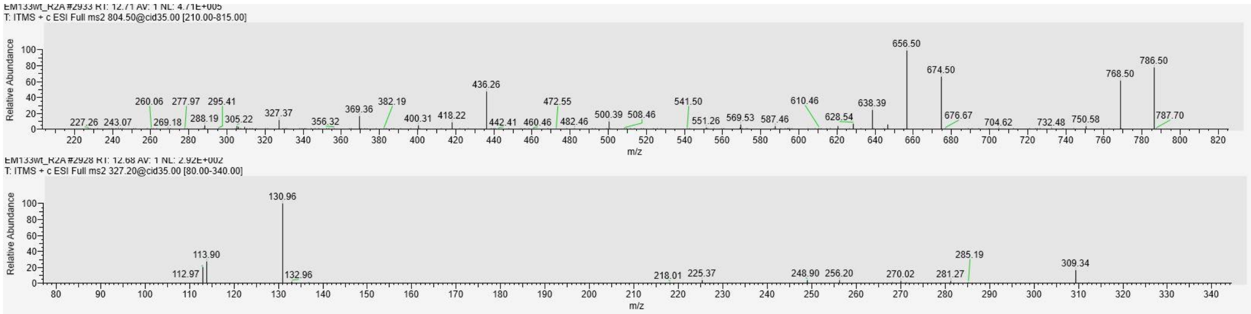

Figure S33: Fragmentation of bokeelamide H (**8**); MS^2 of the parent 804.5 *m/z* (top) and pseudo-MS^3 of the precursor de-acetylated b_1 fragment, 327.2 *m/z* (bottom).

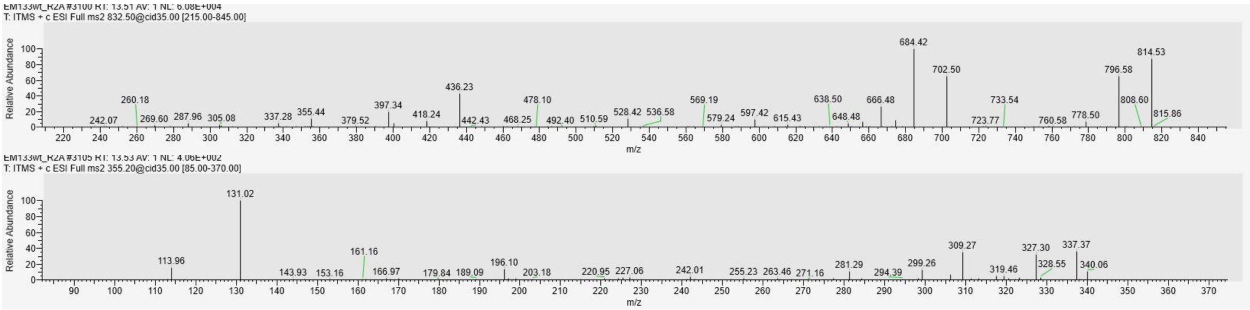

Figure S34: Fragmentation of bokeelamide I (**9**); MS^2 of the parent 832.5 *m/z* (top) and pseudo-MS^3 of the precursor de-acetylated b_1 fragment, 355.2 *m/z* (bottom).

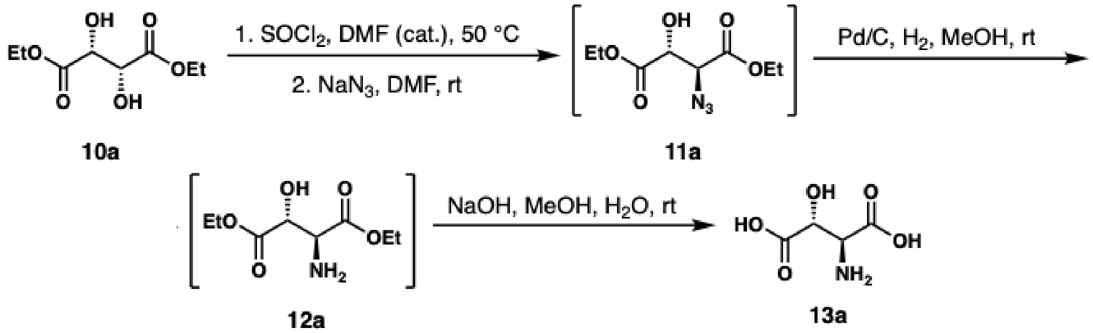

Scheme S1: Synthetic scheme for L-*erythro*-βOH-Aspartic acid (**13a**) from L-diethyl tartrate (**10a**)

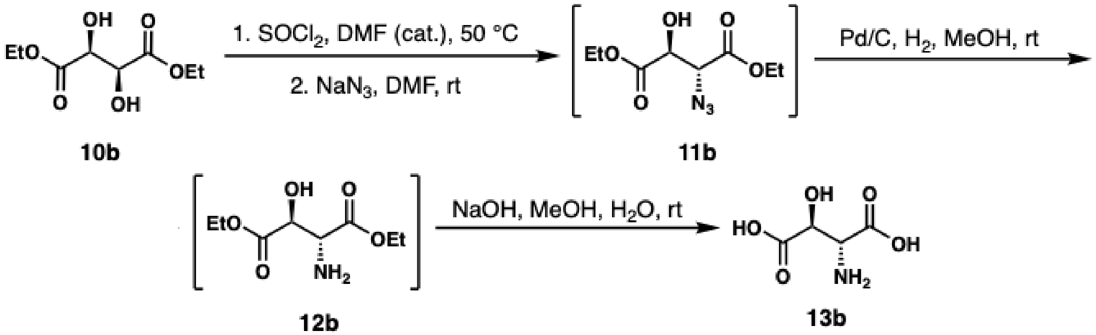

Scheme S2: Synthetic scheme for D-*erythro*-βOH-aspartic acid (**13b**) from D-diethyl tartrate (**10b**)

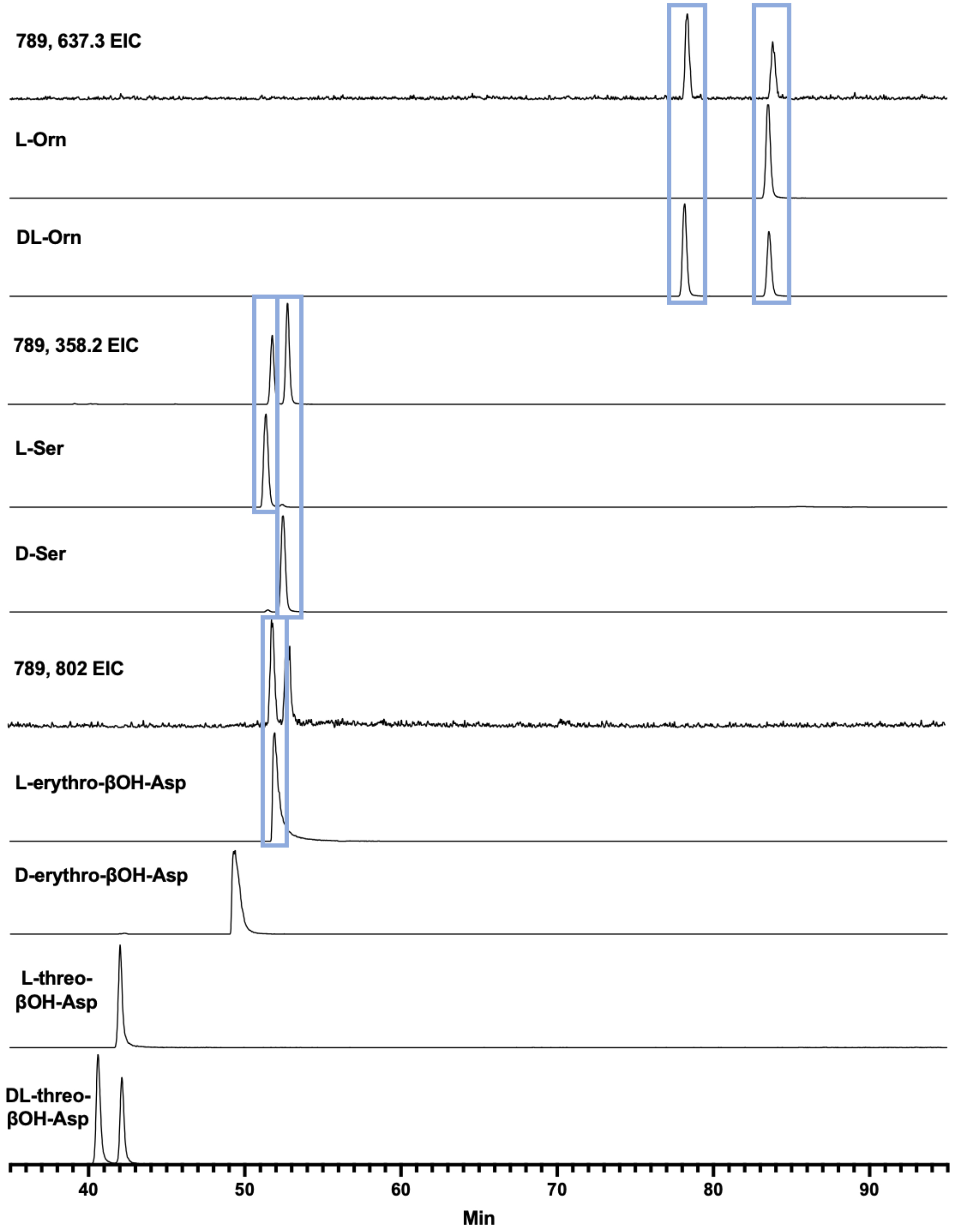

Figure S35. Marfey's analysis of bokeelamide A (**1**) indicating the presence of both L- and D-Ornithine, both L- and D-Serine, and L-*erythro*-βOH-aspartic acid

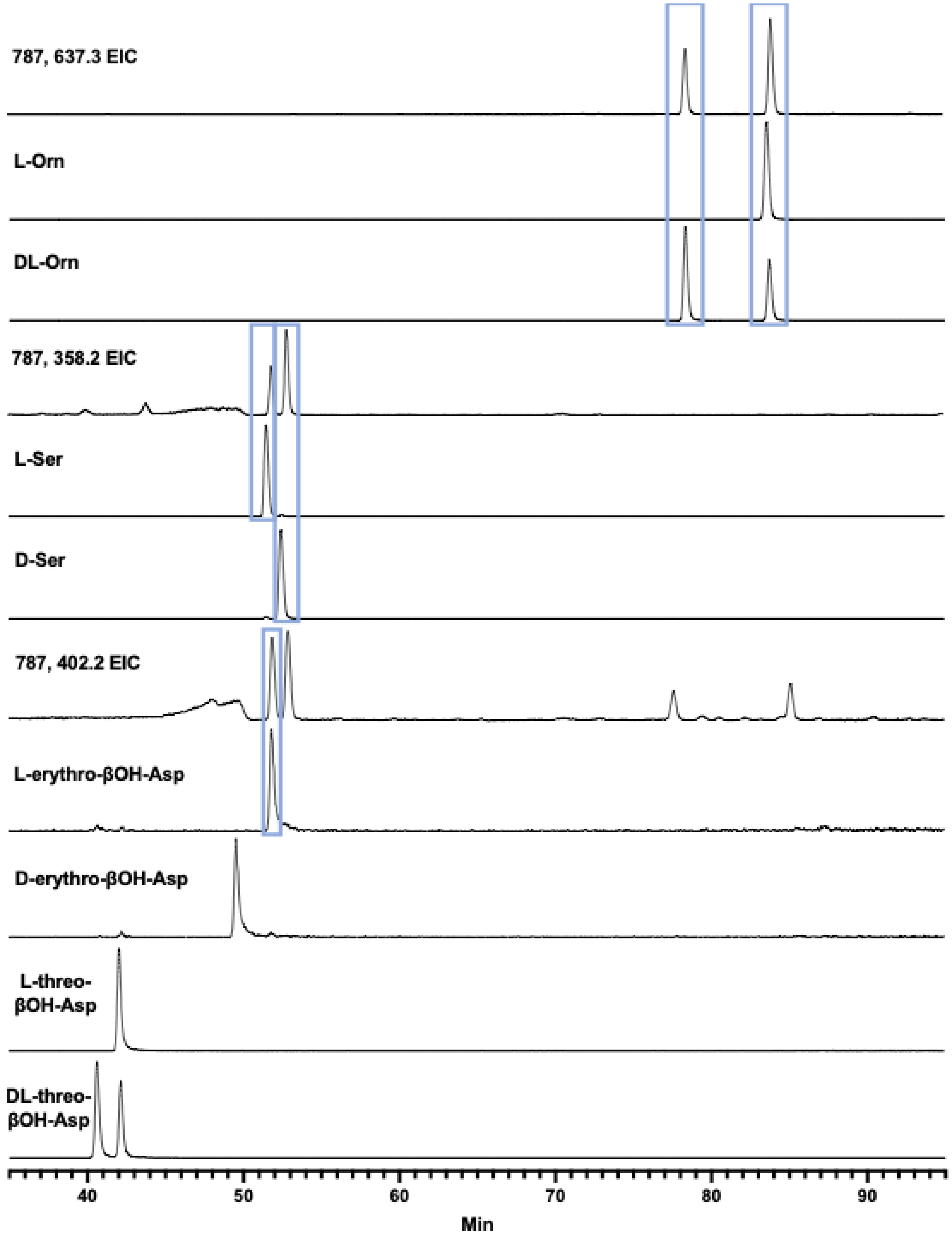

Figure S36: Marfey's analysis of bokeelamide B (**2**) indicating the presence of both L- and D-Ornithine, both L- and D-Serine, and L-*erythro*-βOH-aspartic acid

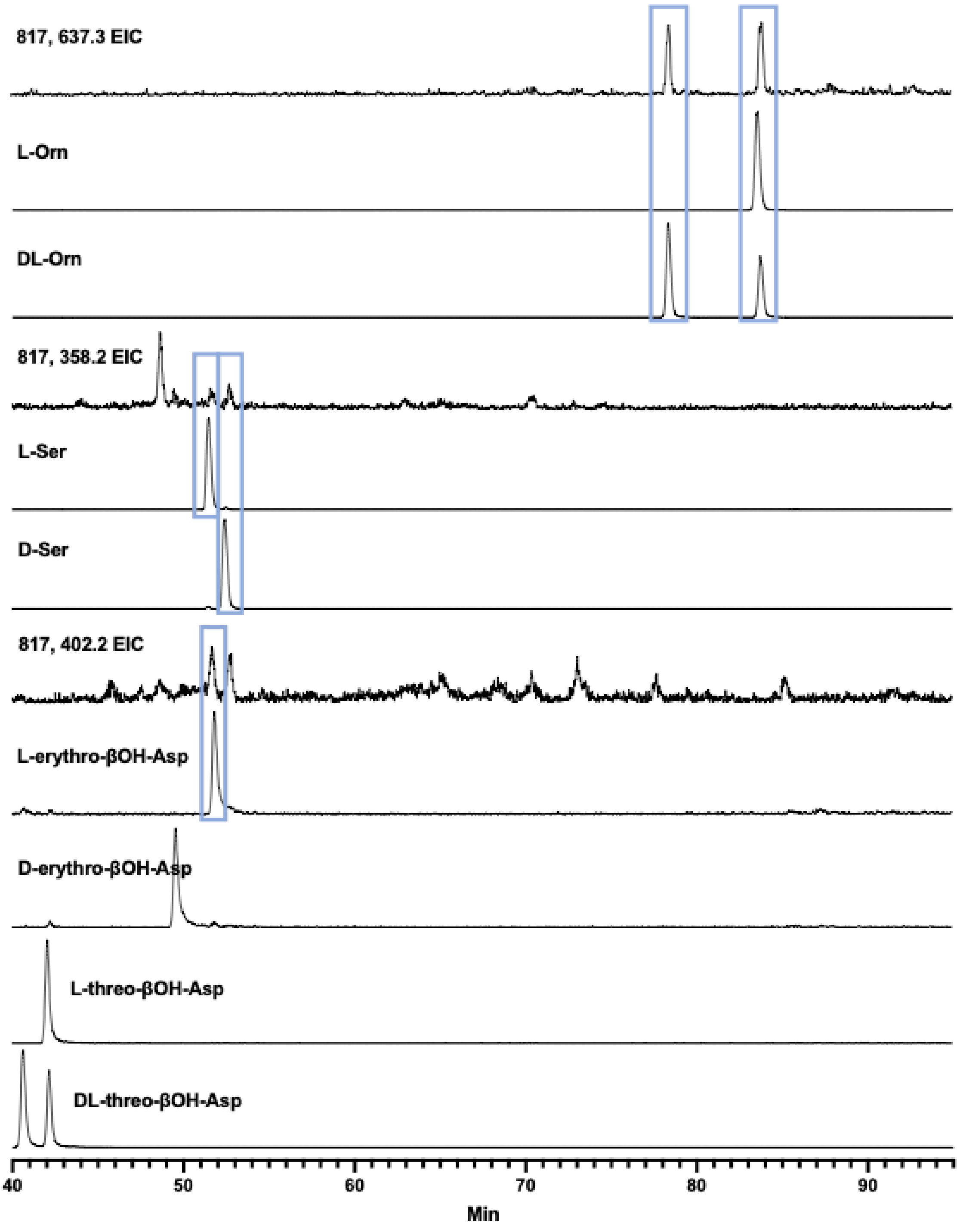

Figure S37: Marfey's analysis of bokeelamide C (**3**) indicating the presence of both L- and D-Ornithine, both L- and D-Serine, and L-*erythro*-βOH-aspartic acid

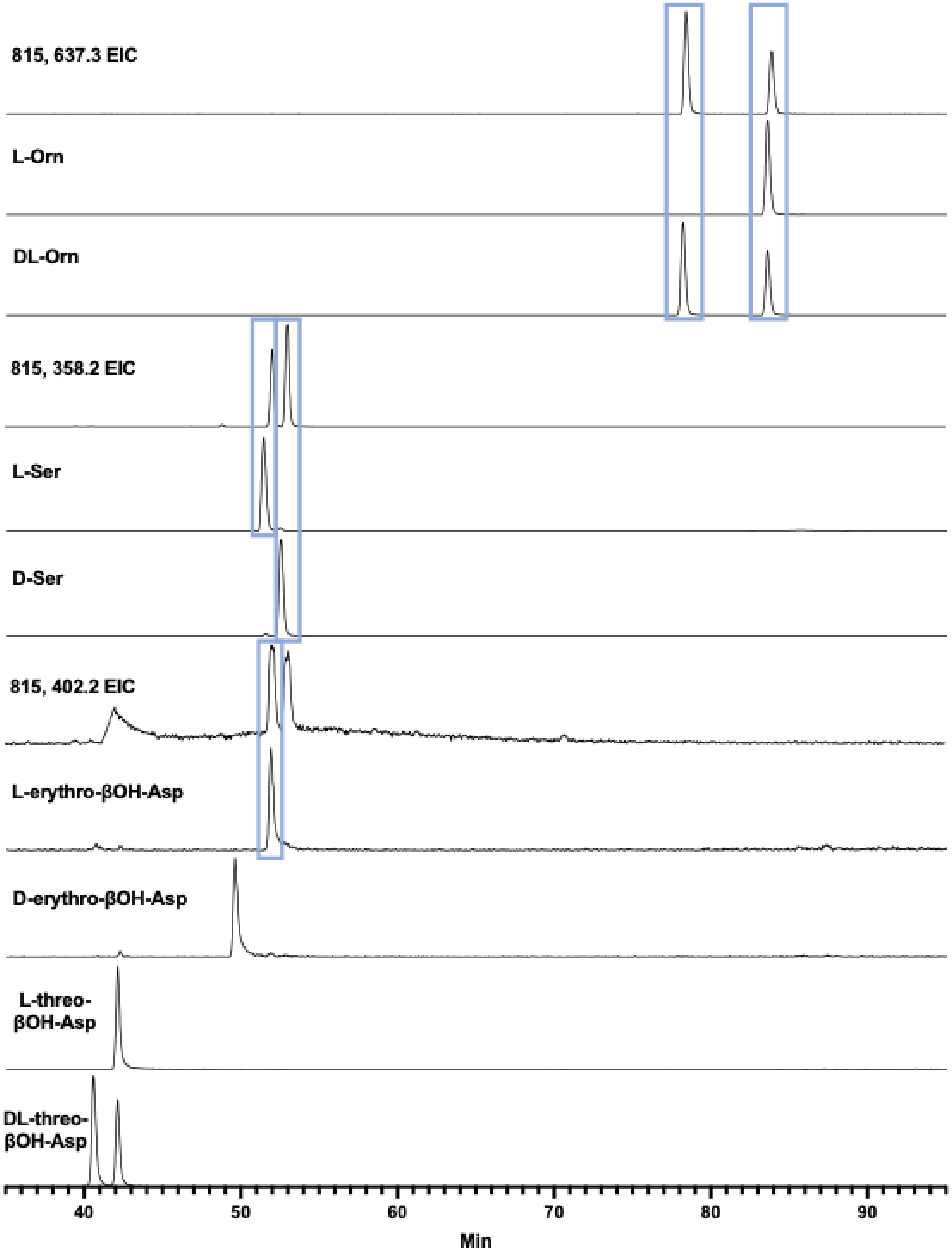

Figure S38: Marfey's analysis of bokeelamide D (**4**) indicating the presence of both L- and D-Ornithine, both L- and D-Serine, and L-*erythro*-βOH-aspartic acid

GENOMIC STUDIES OF *ECTOPSEUDOMONAS KHAZARICA* EM133

Table S6: Tools used by SeqCenter for genome assembly and annotation

Tool	Version	Parameters
porechop	0.2.4	Default parameters
flye	2.9.2	--asm-coverage 50 --genome-size 6000000 --nano-hq
circulator	1.5.5	All; 6-hour timeout
bakta	1.8.1	Default parameters; db version 5.0
quast	5.2.0	Default parameters

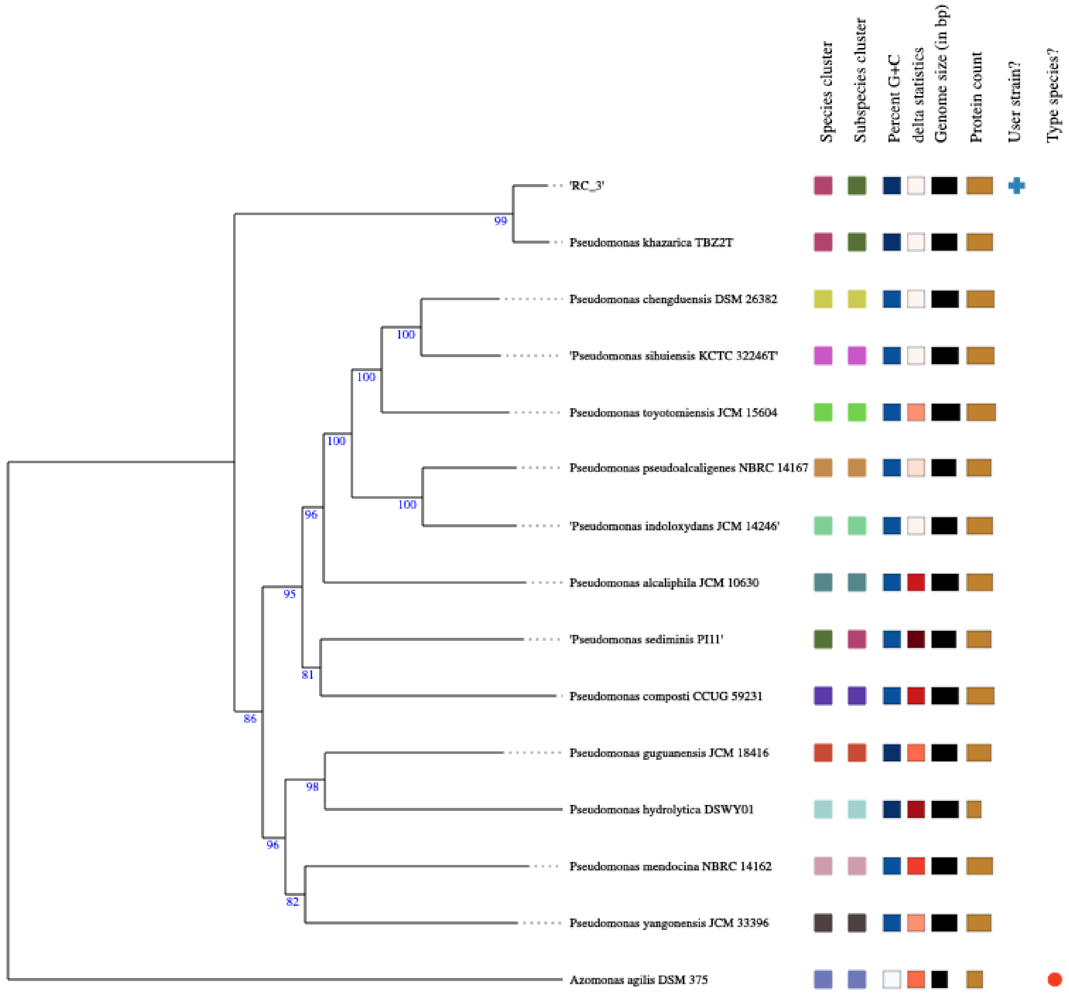

Figure S39: Genomic phylogram of *E. khazarica* EM 133 generated by the Type Genome Server. "RC_3" is the query genome sequence.[10]

Table S7: Genes within the *bka* biosynthetic gene cluster and their predicted product

Name	Length	Strand	Predicted Protein Function
bkaA	656	-	Transcriptional regulator, AcrR family
bkaB	917	-	Transcriptional activator MetR
bkaC	2297	+	5-methyltetrahydropteroyltriglutamate--homocysteine methyltransferase (EC 2.1.1.14)
bkaD	1007	+	Abhydrolase_6, alpha/beta hydrolase fold protein
bkaE	260	-	FIG00961379: hypothetical protein
bkaF	968	+	Transporter, EamA family
bkaG	467	-	Transcriptional regulator, AsnC family
bkaH	3467	+	Indolepyruvate ferredoxin oxidoreductase, α and β subunits
bkaI	1112	+	Branched-chain amino acid dehydrogenase [deaminating] (EC 1.4.1.9)(EC 1.4.1.23)
bkaJ	506	-	Sigma factor PvdS, controlling pyoverdin biosynthesis
bkaK	218	+	MbtH-like NRPS chaperone
bkaL	734	+	Abhydrolase_6; probable thioesterase involved in NRPS, PA2411 homolog
bkaM	971	+	Tyramine beta hydroxylase_Asp, dioxygenase TauD
bkaN	12785	+	Polyketide synthase modules and related proteins
bkaO	9092	+	Polyketide synthase modules and related proteins
bkaP	968	+	COG0657: Esterase/lipase
bkaQ	1994	-	Ferric hydroxamate ABC transporter (TC 3.A.1.14.3), permease component FhuB
bkaR	866	-	Iron compound ABC transporter, periplasmic
bkaS	2423	-	Ferrichrome-iron receptor, TonB-dependent siderophore receptor
bkaT	1691	-	PvdE, pyoverdine ABC export system, fused ATPase and permease components
bkaU	998	-	hypothetical protein
bkaV	386	+	FIG006045: Sigma factor, ECF subfamily
bkaW	1322	+	L-ornithine 5-monooxygenase (EC 1.13.12.-), PvdA of pyoverdin biosynthesis
bkaX	980	+	GNAT family N-acetyltransferase
bkaY	749	+	hypothetical protein
bkaZ	767	-	Ferric hydroxamate ABC transporter (TC 3.A.1.14.3), ATP-binding protein FhuC

BIOLOGICAL STUDIES

Table S8: Media Recipes

YEME	R2A	A-media	M63	LB	YPD
4 g yeast extract	0.5 g acicase	20 g starch	2 g $(NH_4)SO_4$	10 g tryptone	10 g yeast extract
10 g malt extract	0.5 g yeast extract	5 g peptone	13.6 g KH_2PO_4	5 g yeast extract	20 g peptone
4 g dextrose	0.5 g proteose peptone	10 g dextrose	0.5 mg $FeSO_4$ •$7H_2O$	10 g NaCl	20 g dextrose
1 L seawater	0.5 g dextrose	5 g yeast	1 mL 1 M $MgSO_4$•$7H_2O$	1 L DI water or seawater	1 L DI water
	0.5 g starch, soluble	5 g $CaCO_3$	10 mL 20% dextrose		
	0.3 g K_2HPO_4	1 L seawater	0.1 mL 0.5% thiamine		
	0.024 g $MgSO_4$		5 mL 20% casamino acids		
	0.3 g sodium pyruvate		1 L DI water		
	1 L seawater				

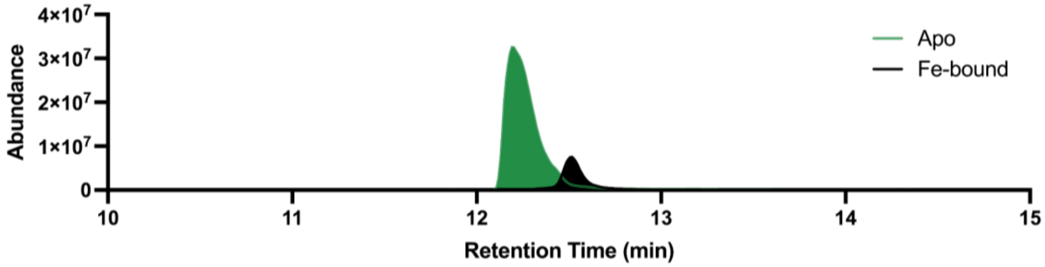

Figure S40: Overlaid EICs of apo-bokeelamide A (**1**, green, 790.5 *m/z*) and iron-bound form (black, 843.5 *m/z*)

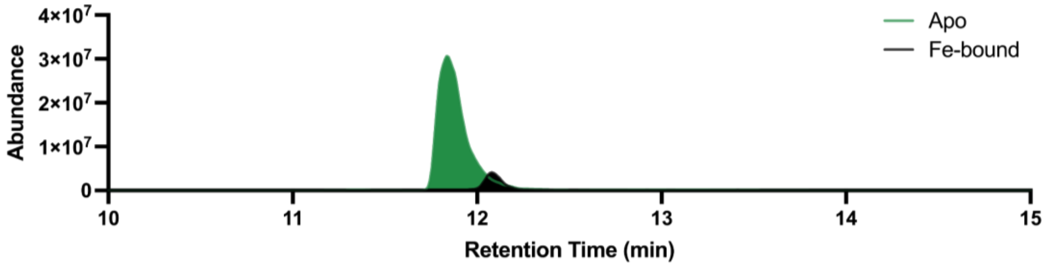

Figure S41: Overlaid EICs of apo-bokeelamide B (**2**, green, 788.5 *m/z*) and iron-bound form (black, 841.5 *m/z*)

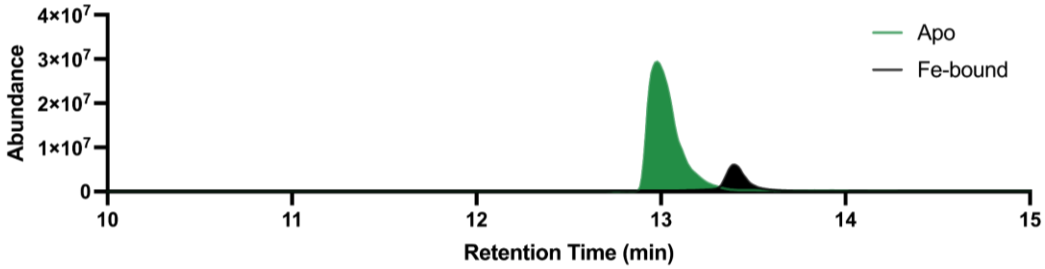

Figure S42: Overlaid EICs of apo-bokeelamide C (**3**, green, 818.5 *m/z*) and iron-bound form (black, 871.5 *m/z*)

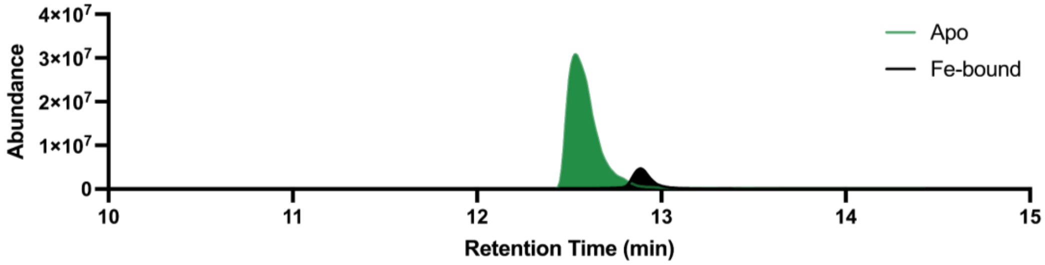

Figure S43: Overlaid EICs of apo-bokeelamide D (**4**, green, 816.5 *m/z*) and iron-bound form (black, 869.5 *m/z*)

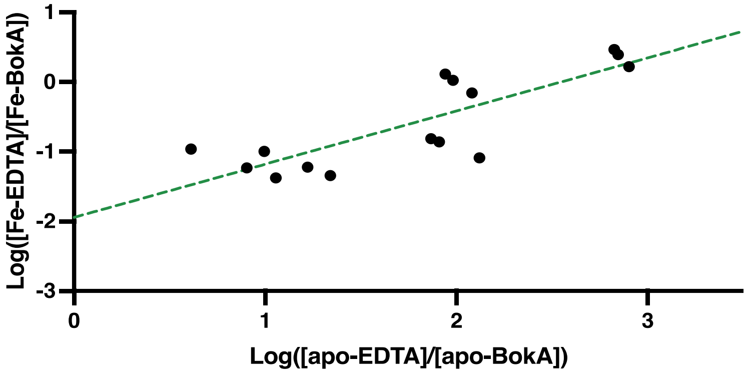

Figure S44: Superimposed replicates of apo- vs iron-bound concentration log ratios in a competition titration of bokeelamide A (**1**) against EDTA at 0.1 mM Fe-**1** and 0.05-50 mM EDTA (average x intercept = ΔpFe^{3+} = +2.5 ±0.2).

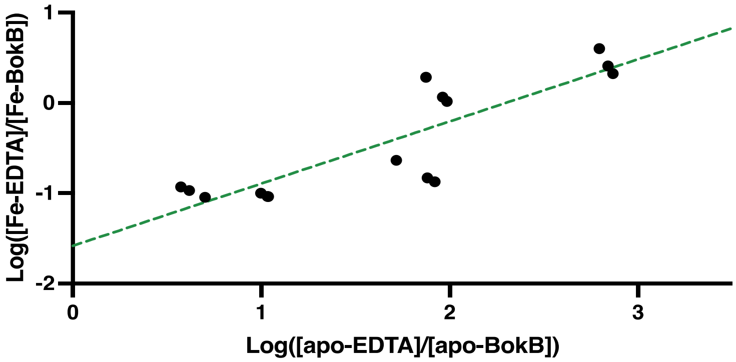

Figure S45: Superimposed replicates of apo- vs iron-bound concentration log ratios in a competition titration of bokeelamide B (**2**) against EDTA at 0.1 mM Fe-**2** and 0.05-50 mM EDTA (average x intercept = ΔpFe^{3+} = +2.3 ± 0.2).

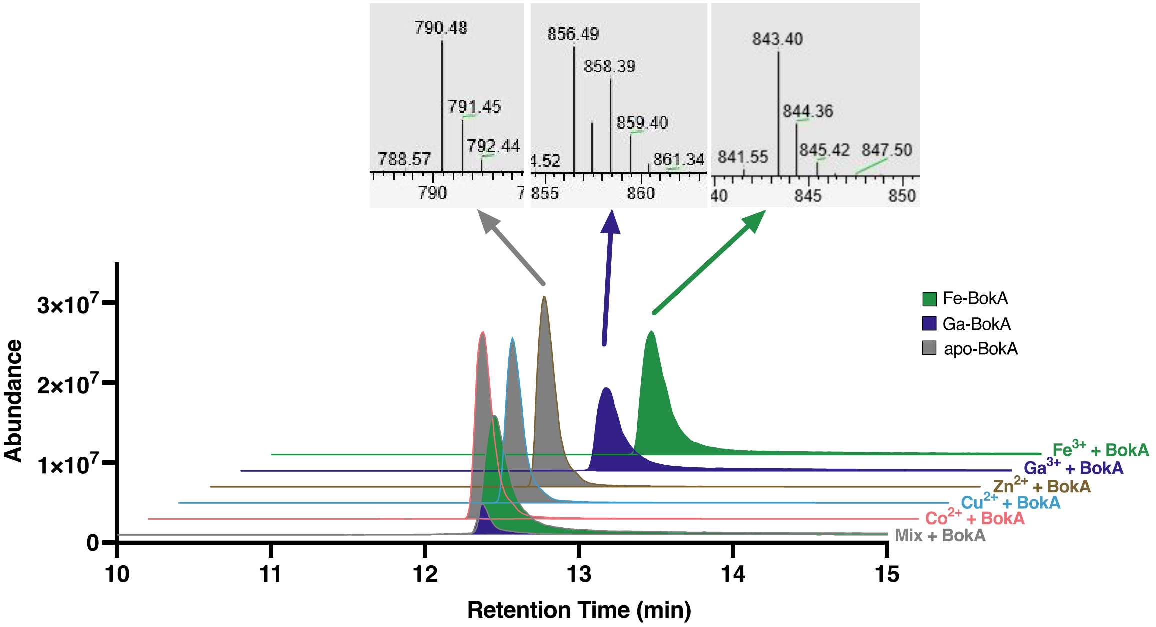

Figure S46: LCMS traces of five single-metal (color traces) and one mixed-metal (gray trace) samples in a binding selectivity assay against bokeelamide A (**1**), showing EICs of apo-**1** (790.5 *m/z*, gray fill), Co^{2+}-**1** (847.4 *m/z*, pink), Cu^{2+}-**1** (851.4 *m/z*, blue), Zn^{2+}-**1** (852.4 *m/z*, brown), Ga^{3+}-**1** (856.4 *m/z*, purple fill), and Fe^{3+}-**1** (843.4 *m/z*, green fill), showing selective chelation of Fe^{3+} and Ga^{3+} and preference for Fe^{3+}.

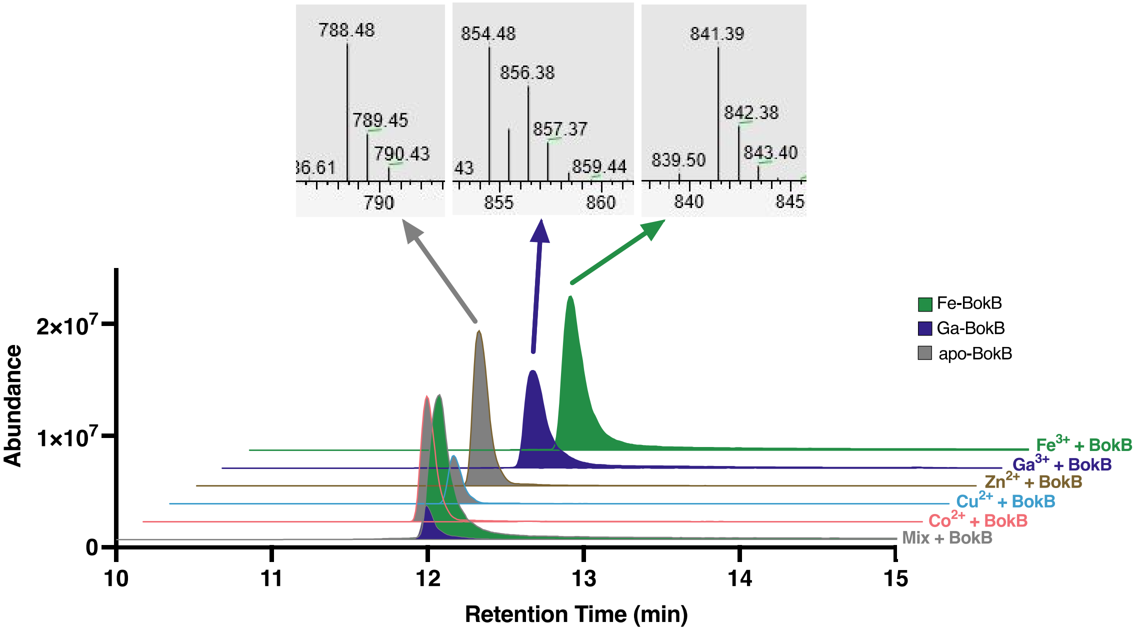

Figure S47: LCMS traces of five single-metal (color traces) and one mixed-metal (gray trace) samples in a binding selectivity assay against bokeelamide B (**2**), showing EICs of apo-**2** (788.5 *m/z*, gray fill), Co^{2+}-**2** (845.4 *m/z*, pink), Cu^{2+}-**2** (849.4 *m/z*, blue), Zn^{2+}-**2** (850.4 *m/z*, brown), Ga^{3+}-**2** (854.4 *m/z*, purple fill), and Fe^{3+}-**2** (841.4 *m/z*, green fill), showing selective chelation of Fe^{3+} and Ga^{3+} and preference for Fe^{3+}.

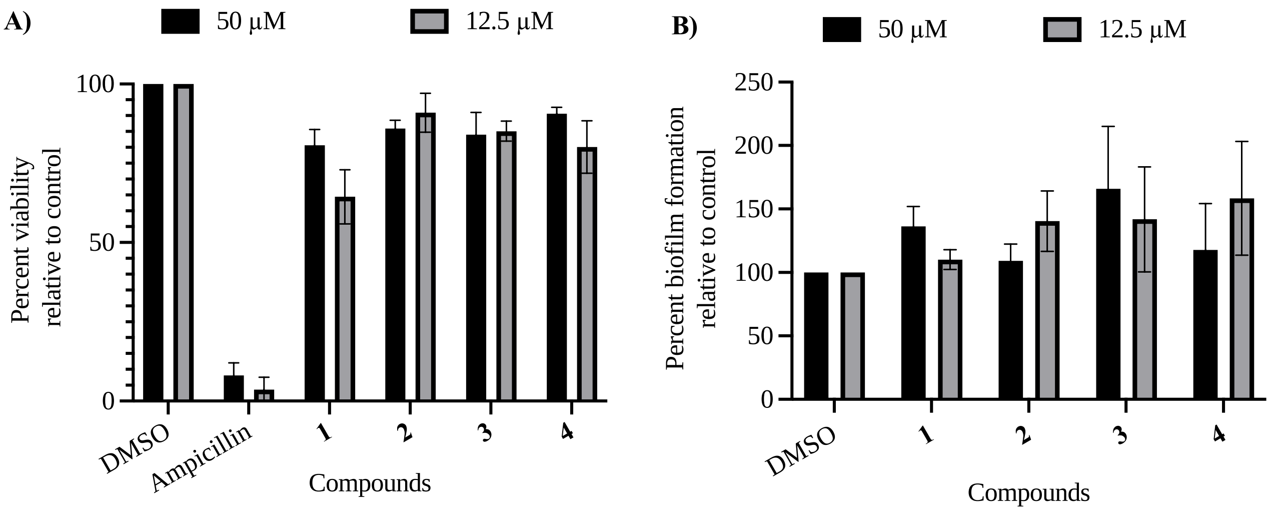

Figure S48: Bokeelamides A - D (**1** - **4**) **A)** antimicrobial activity and **B)** biofilm inhibition activity against *S. aureus*.

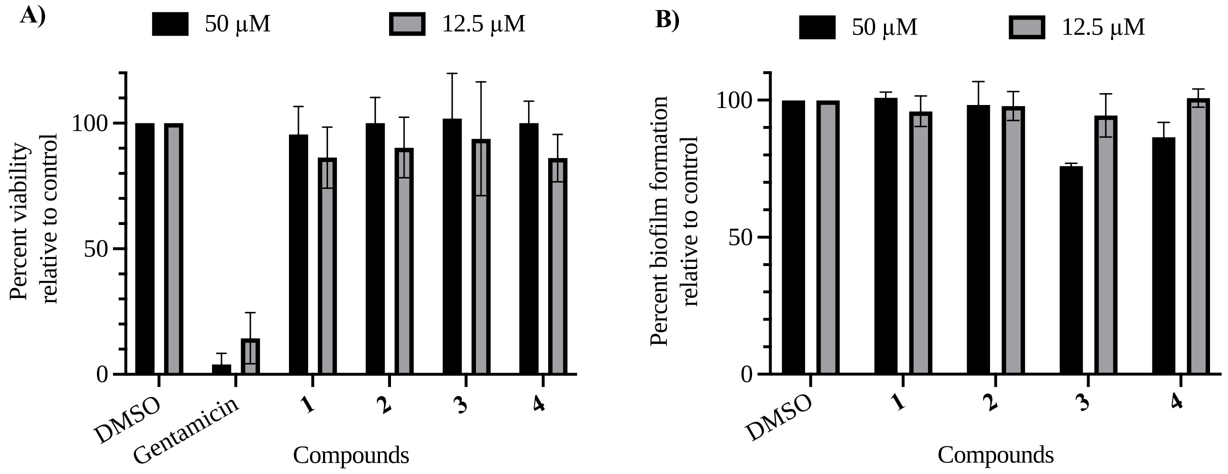

Figure S49: Bokeelamides A - D (**1** - **4**) **A)** antimicrobial activity and **B)** biofilm inhibition activity against *P. aeruginosa*.

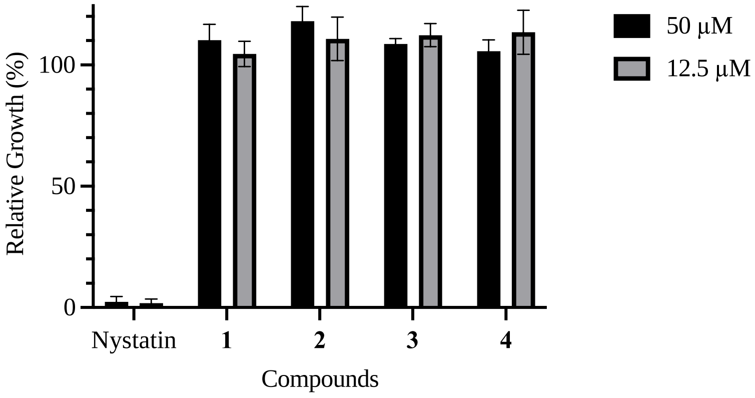

Figure S50: Bokeelamides A - D (**1** - **4**) against *C. albicans*

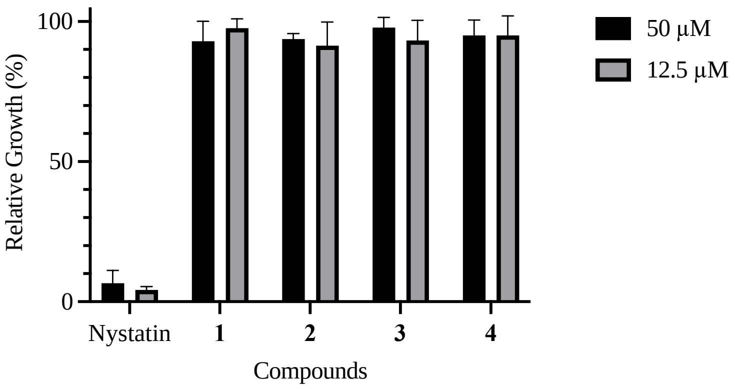

Figure S51: Bokeelamides A - D (**1** - **4**) against *A. flavus*

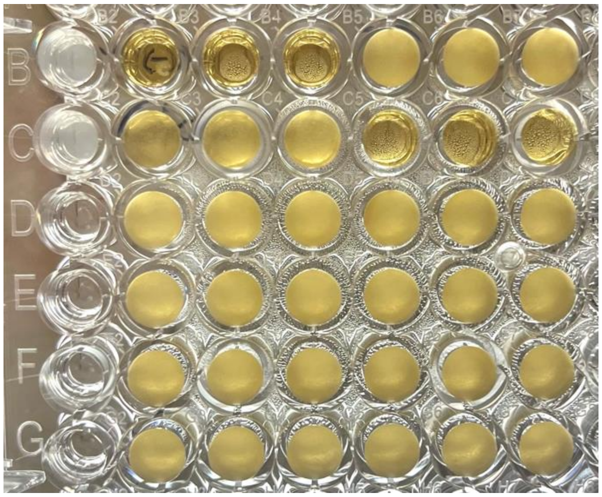

Figure S52: Bokeelamides A - D (**1** - **4**) against *Fusarium keritoplasticum* [**1**: D2-4 (12.5 µM), D5-7 (50 µM); **2**: E2-4 (12.5 µM), E5-7 (50 µM); **3**: F2-4 (12.5 µM), F5-7 (50 µM); **4**: G2-4 (12.5 µM), G5-7 (50 µM); Cycloheximide: C2-4 (12.5 µM), C5-7 (50 µM); DMSO: B5-7 sterile media: B2-4;]. Absorbance was not taken due to clumping of mycelia.

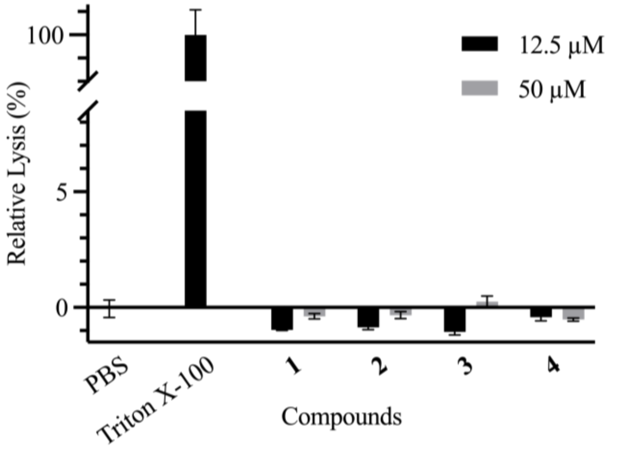

Figure S53: Relative percent hemolysis of bokeelamides A - D (**1** - **4**) at 12.5 µM (black) and 50 µM (gray). PBS was used as a negative control and Triton X-100 as a positive control.

REFERENCES

(1) Kyei, L.; Piedl, K.; Menegatti, C.; Miller, E. M.; Mevers, E. Discovery of Biofilm Inhibitors from the Microbiota of Marine Egg Masses. *J. Nat. Prod.* **2024**, *87* (6), 1635–1642.

(2) Wang, M.; Carver, J. J.; Phelan, V. V.; Sanchez, L. M.; Garg, N.; Peng, Y.; Nguyen, D. D.; Watrous, J.; Kapono, C. A.; Luzzatto-Knaan, T.; Porto, C.; Bouslimani, A.; Melnik, A. V.; Meehan, M. J.; Liu, W.-T.; Crüsemann, M.; Boudreau, P. D.; Esquenazi, E.; Sandoval-Calderón, M.; Kersten, R. D.; Pace, L. A.; Quinn, R. A.; Duncan, K. R.; Hsu, C.-C.; Floros, D. J.; Gavilan, R. G.; Kleigrewe, K.; Northen, T.; Dutton, R. J.; Parrot, D.; Carlson, E. E.; Aigle, B.; Michelsen, C. F.; Jelsbak, L.; Sohlenkamp, C.; Pevzner, P.; Edlund, A.; McLean, J.; Piel, J.; Murphy, B. T.; Gerwick, L.; Liaw, C.-C.; Yang, Y.-L.; Humpf, H.-U.; Maansson, M.; Keyzers, R. A.; Sims, A. C.; Johnson, A. R.; Sidebottom, A. M.; Sedio, B. E.; Klitgaard, A.; Larson, C. B.; Boya P, C. A.; Torres-Mendoza, D.; Gonzalez, D. J.; Silva, D. B.; Marques, L. M.; Demarque, D. P.; Pociute, E.; O'Neill, E. C.; Briand, E.; Helfrich, E. J. N.; Granatosky, E. A.; Glukhov, E.; Ryffel, F.; Houson, H.; Mohimani, H.; Kharbush, J. J.; Zeng, Y.; Vorholt, J. A.; Kurita, K. L.; Charusanti, P.; McPhail, K. L.; Nielsen, K. F.; Vuong, L.; Elfeki, M.; Traxler, M. F.; Engene, N.; Koyama, N.; Vining, O. B.; Baric, R.; Silva, R. R.; Mascuch, S. J.; Tomasi, S.; Jenkins, S.; Macherla, V.; Hoffman, T.; Agarwal, V.; Williams, P. G.; Dai, J.; Neupane, R.; Gurr, J.; Rodríguez, A. M. C.; Lamsa, A.; Zhang, C.; Dorrestein, K.; Duggan, B. M.; Almaliti, J.; Allard, P.-M.; Phapale, P.; Nothias, L.-F.; Alexandrov, T.; Litaudon, M.; Wolfender, J.-L.; Kyle, J. E.; Metz, T. O.; Peryea, T.; Nguyen, D.-T.; VanLeer, D.; Shinn, P.; Jadhav, A.; Müller, R.; Waters, K. M.; Shi, W.; Liu, X.; Zhang, L.; Knight, R.; Jensen, P. R.; Palsson, B. Ø.; Pogliano, K.; Linington, R. G.; Gutiérrez, M.; Lopes, N. P.; Gerwick, W. H.; Moore, B. S.; Dorrestein, P. C.; Bandeira, N. Sharing and Community Curation of Mass Spectrometry Data with Global Natural Products Social Molecular Networking. *Nat. Biotechnol.* **2016**, *34* (8), 828–837.

(3) Liu, L.; Wang, B.; Bi, C.; He, G.; Chen, G. Efficient Preparation of β-Hydroxy Aspartic Acid and Its Derivatives. *Chin. Chem. Lett.* **2018**, *29* (7), 1113–1115.

(4) Salvador, L. A.; Biggs, J. S.; Paul, V. J.; Luesch, H. Veraguamides A-G, Cyclic Hexadepsipeptides from a Dolastatin 16-Producing Cyanobacterium Symploca Cf. Hydnoides from Guam. *J. Nat. Prod.* **2011**, *74* (5), 917–927.

(5) Bonenfant, Q.; Noé, L.; Touzet, H. Porechop_ABI: Discovering Unknown Adapters in Oxford Nanopore Technology Sequencing Reads for Downstream Trimming. *Bioinform. Adv.* **2023**, *3* (1), vbac085.

(6) Kolmogorov, M.; Yuan, J.; Lin, Y.; Pevzner, P. A. Assembly of Long, Error-Prone Reads Using Repeat Graphs. *Nat. Biotechnol.* **2019**, *37* (5), 540–546.

(7) Hunt, M.; Silva, N. D.; Otto, T. D.; Parkhill, J.; Keane, J. A.; Harris, S. R. Circlator: Automated Circularization of Genome Assemblies Using Long Sequencing Reads. *Genome Biol.* **2015**, *16* (1), 294.

(8) Schwengers, O.; Jelonek, L.; Dieckmann, M. A.; Beyvers, S.; Blom, J.; Goesmann, A. Bakta: Rapid and Standardized Annotation of Bacterial Genomes via Alignment-Free Sequence Identification. *Microb. Genom.* **2021**, *7* (11). https://doi.org/10.1099/mgen.0.000685.

(9) Gurevich, A.; Saveliev, V.; Vyahhi, N.; Tesler, G. QUAST: Quality Assessment Tool for Genome Assemblies. *Bioinformatics* **2013**, *29* (8), 1072–1075.

(10) Meier-Kolthoff, J. P.; Göker, M. TYGS Is an Automated High-Throughput Platform for State-of-the-Art Genome-Based Taxonomy. *Nat. Commun.* **2019**, *10* (1), 2182.

(11) Blin, K.; Shaw, S.; Augustijn, H. E.; Reitz, Z. L.; Biermann, F.; Alanjary, M.; Fetter, A.; Terlouw, B. R.; Metcalf, W. W.; Helfrich, E. J. N.; van Wezel, G. P.; Medema, M. H.; Weber, T. AntiSMASH 7.0: New and Improved Predictions for Detection, Regulation, Chemical Structures and Visualisation. *Nucleic Acids Res.* **2023**, *51* (W1), W46–W50.

(12) Brettin, T.; Davis, J. J.; Disz, T.; Edwards, R. A.; Gerdes, S.; Olsen, G. J.; Olson, R.; Overbeek, R.; Parrello, B.; Pusch, G. D.; Shukla, M.; Thomason, J. A., 3rd; Stevens, R.; Vonstein, V.; Wattam, A. R.; Xia, F. RASTtk: A Modular and Extensible Implementation of the RAST Algorithm for Building Custom Annotation Pipelines and Annotating Batches of Genomes. *Sci. Rep.* **2015**, *5* (1), 8365.

(13) Bachmann, B. O.; Ravel, J. Chapter 8. Methods for in Silico Prediction of Microbial Polyketide and Nonribosomal Peptide Biosynthetic Pathways from DNA Sequence Data. *Methods Enzymol.* **2009**, *458*, 181–217.

(14) Fukuda, T. T. H.; Helfrich, E. J. N.; Mevers, E.; Melo, W. G. P.; Van Arnam, E. B.; Andes, D. R.; Currie, C. R.; Pupo, M. T.; Clardy, J. Specialized Metabolites Reveal Evolutionary History and Geographic Dispersion of a Multilateral Symbiosis. *ACS Cent. Sci.* **2021**, *7* (2), 292–299.

(15) Abergel, R. J.; Zawadzka, A. M.; Raymond, K. N. Petrobactin-Mediated Iron Transport in Pathogenic Bacteria: Coordination Chemistry of an Unusual 3,4-Catecholate/Citrate Siderophore. *J. Am. Chem. Soc.* **2008**, *130* (7), 2124–2125.

(16) Kerwin, A. H.; Gromek, S. M.; Suria, A. M.; Samples, R. M.; Deoss, D. J.; O'Donnell, K.; Frasca, S., Jr; Sutton, D. A.; Wiederhold, N. P.; Balunas, M. J.; Nyholm, S. V. Shielding the next Generation: Symbiotic Bacteria from a Reproductive Organ Protect Bobtail Squid Eggs from Fungal Fouling. *MBio* **2019**, *10* (5). https://doi.org/10.1128/mBio.02376-19.

(17) Evans, B. C.; Nelson, C. E.; Yu, S. S.; Beavers, K. R.; Kim, A. J.; Li, H.; Nelson, H. M.; Giorgio, T. D.; Duvall, C. L. Ex Vivo Red Blood Cell Hemolysis Assay for the Evaluation of PH-Responsive Endosomolytic Agents for Cytosolic Delivery of Biomacromolecular Drugs. *J. Vis. Exp.* **2013**, No. 73, e50166.
